# Supplementary material for: Multi-walled carbon nanotubes produced after forest fires improve germination and development of Eysenhardtia polystachya
Source: PeerJ. 2020 Apr 23;8:e8634. doi: 10.7717/peerj.8634 (PMC7183754; doi:10.7717/peerj.8634)
Supplement: Supplemental Information 1 [file peerj-08-8634-s001.zip › programa.pdf]

PROGRAMA DE MANEJO

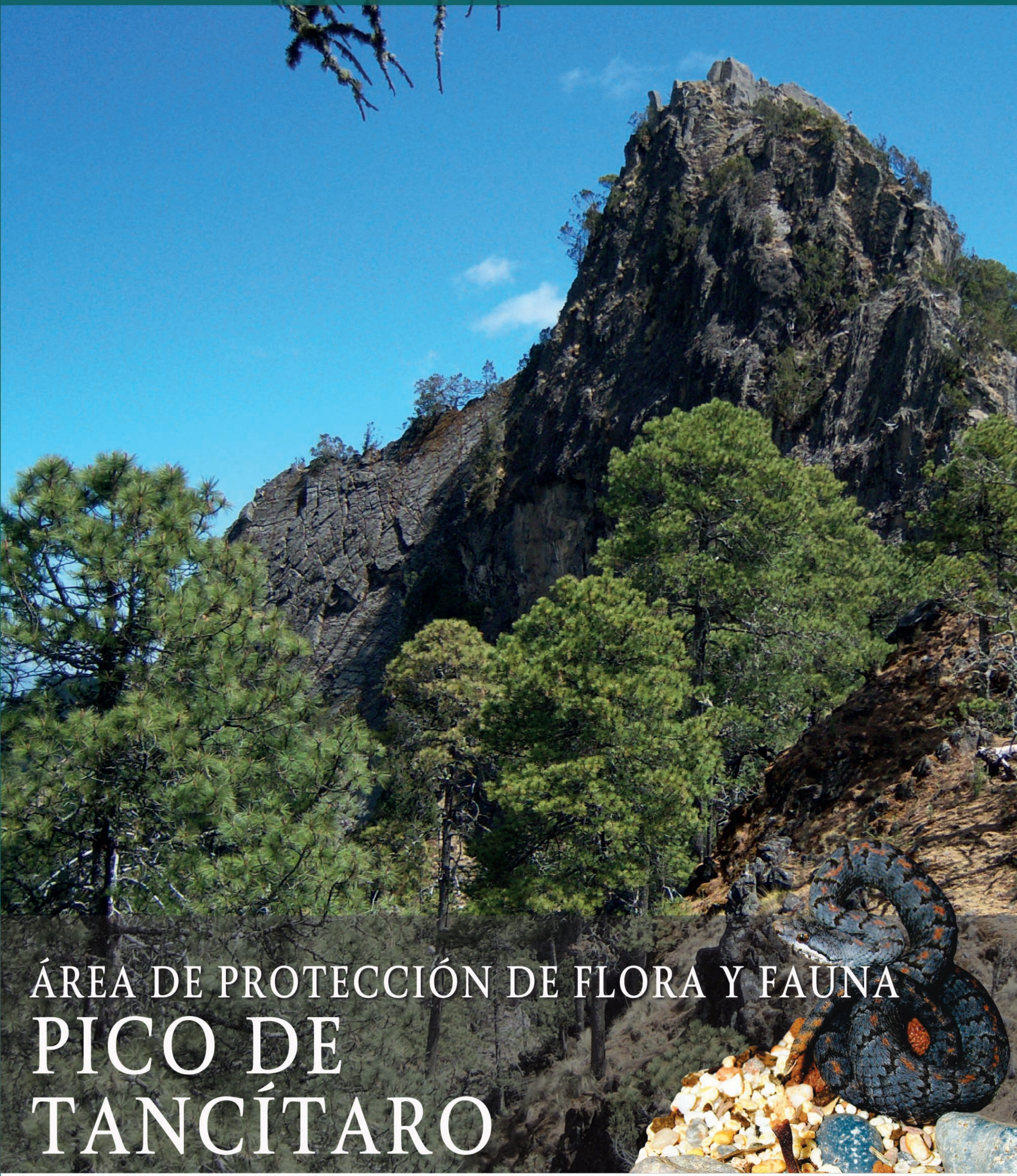

ÁREA DE PROTECCIÓN DE FLORA Y FAUNA

PICO DE TANCÍTARO

MÉXICO
GOBIERNO DE LA REPÚBLICA

SEMARNAT
SECRETARÍA DE MEDIO AMBIENTE Y RECURSOS NATURALES

CONANP
COMISIÓN NACIONAL DE ÁREAS NATURALES PROTEGIDAS

Pico de Tancítaro
Área de Protección de Flora y Fauna

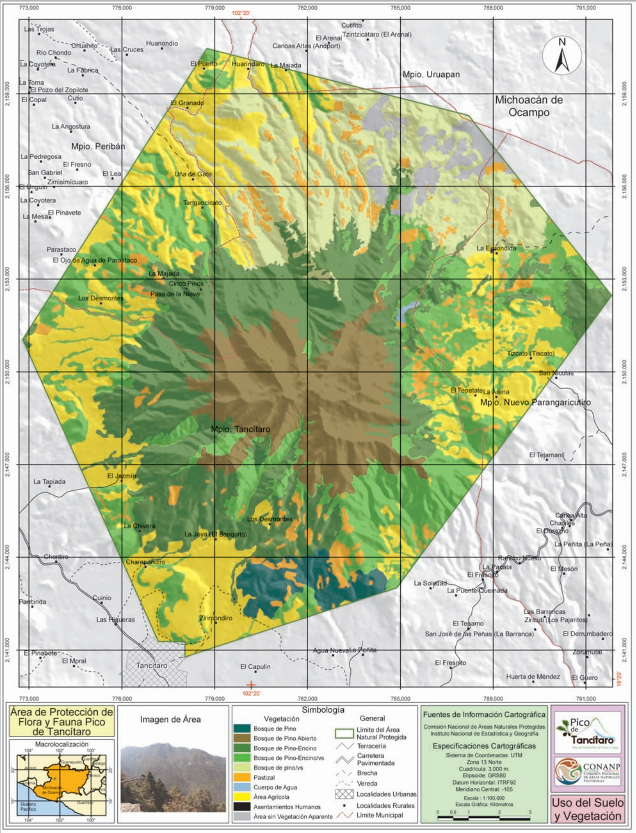
Mpio. Uruapan
Michoacán de Ocampo
Mpio. Peribán
Mpio. Tancítaro
Mpio. Nuevo Parangaricutiro
Área de Protección de Flora y Fauna Pico de Tancítaro
Macrolocalización
Imagen de Área
Simbología
Vegetación
Bosque de Pino
Bosque de Pino Abierto
Bosque de Pino-Encino
Bosque de Pino-Encino/vs
Bosque de pino/vs
Pastizal
Cuerpo de Agua
Área Agrícola
Asentamientos Humanos
Área sin Vegetación Aparente
General
Límite del Área Natural Protegida
Terracería
Carretera Pavimentada
Brecha
Vereda
Localidades Urbanas
Localidades Rurales
Límite Municipal
Fuentes de Información Cartográfica
Comisión Nacional de Áreas Naturales Protegidas
Instituto Nacional de Estadística y Geografía
Especificaciones Cartográficas
Sistema de Coordenadas: UTM
Zona 13 Norte
Cuadrícula: 3,000 m.
Elipsoide: GRS80
Datum Horizontal: ITRF92
Meridiano Central: -105
Escala : 1:100,000
Escala Gráfica: Kilómetros
Pico de Tancítaro
CONANP
Uso del Suelo y Vegetación

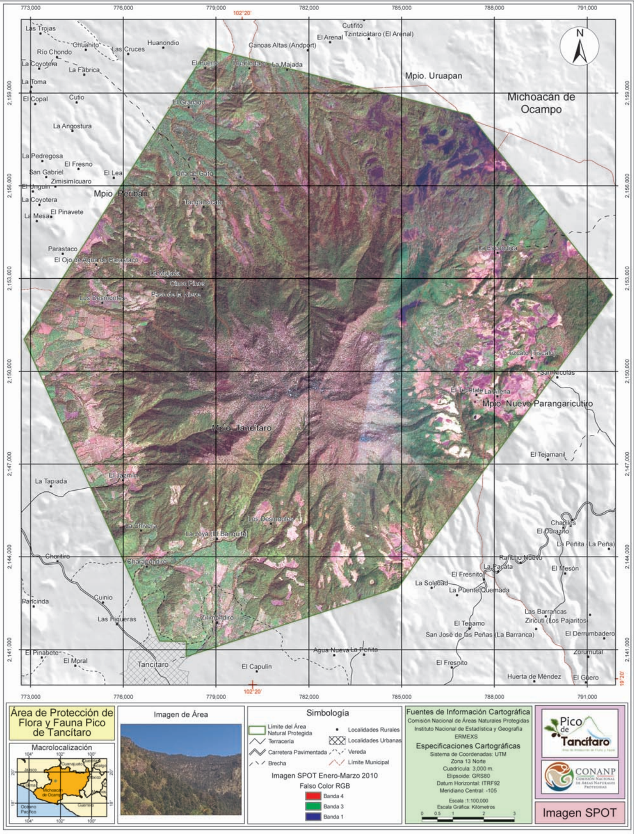
Mpio. Uruapan
Michoacán de Ocampo
Mpio. Peribán
Mpio. Tancítaro
Mpio. Nuevo Parangaricutiro
Área de Protección de Flora y Fauna Pico de Tancítaro
Macrolocalización
Imagen de Área
Simbología
Límite del Área Natural Protegida
Terracería
Carretera Pavimentada
Brecha
Localidades Rurales
Localidades Urbanas
Vereda
Límite Municipal
Imagen SPOT Enero-Marzo 2010
Falso Color RGB
Banda 4
Banda 3
Banda 1
Fuentes de Información Cartográfica
Comisión Nacional de Áreas Naturales Protegidas
Instituto Nacional de Estadística y Geografía
ERMEXS
Especificaciones Cartográficas
Sistema de Coordenadas: UTM
Zona 13 Norte
Cuadrícula: 3,000 m.
Elipsoide: GRS80
Datum Horizontal: ITRF92
Meridiano Central: -105
Escala : 1:100,000
Escala Gráfica: Kilómetros
Pico de Tancítaro
CONANP
Imagen SPOT

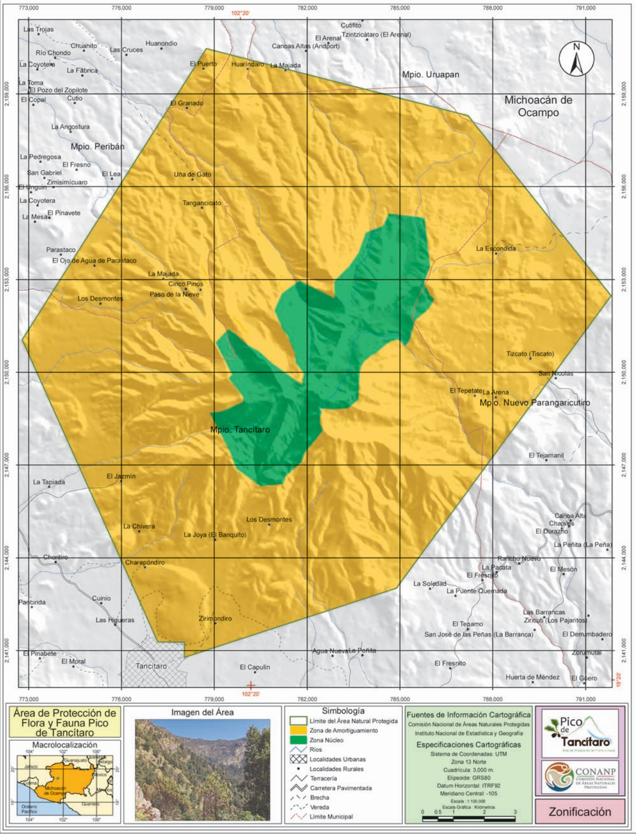
Mpio. Uruapan
Michoacán de Ocampo
Mpio. Peribán
Mpio. Tancítaro
Mpio. Nuevo Parangaricutiro
Área de Protección de Flora y Fauna Pico de Tancítaro
Macrolocalización
Imagen del Área
Simbología
Límite del Área Natural Protegida
Zona de Amortiguamiento
Zona Núcleo
Ríos
Localidades Urbanas
Localidades Rurales
Terracería
Carretera Pavimentada
Brecha
Vereda
Límite Municipal
Fuentes de Información Cartográfica
Comisión Nacional de Áreas Naturales Protegidas
Instituto Nacional de Estadística y Geografía
Especificaciones Cartográficas
Sistema de Coordenadas: UTM
Zona 13 Norte
Cuadrícula: 3,000 m.
Elipsoide: GRS80
Datum Horizontal: ITRF92
Meridiano Central: -105
Escala : 1:100,000
Escala Gráfica: Kilómetros
Pico de Tancítaro
CONANP
Zonificación

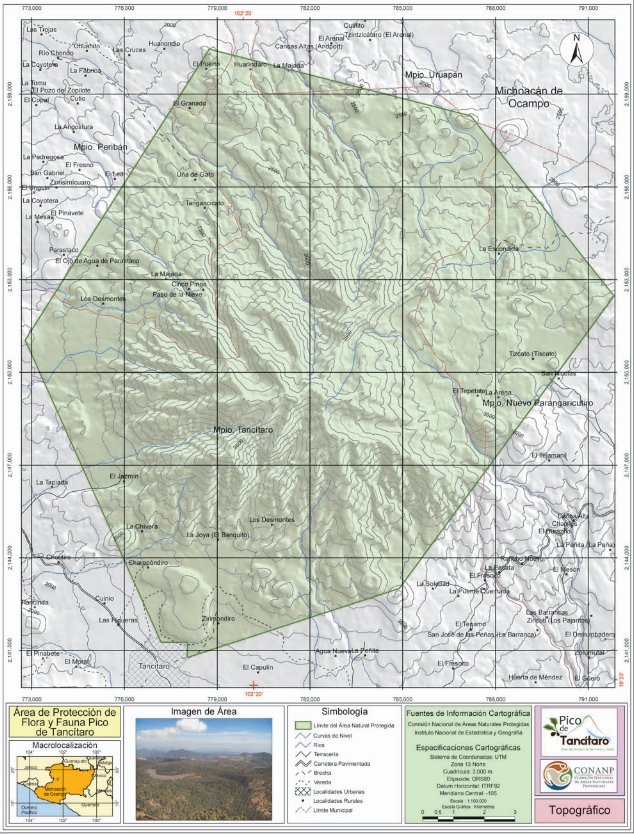
Mpio. Uruapan
Michoacán de Ocampo
Mpio. Peribán
Mpio. Tancítaro
Mpio. Nuevo Parangaricutiro
Área de Protección de Flora y Fauna Pico de Tancítaro
Macrolocalización
Imagen de Área
Simbología
Límite del Área Natural Protegida
Curvas de Nivel
Ríos
Terracería
Carretera Pavimentada
Brecha
Vereda
Localidades Urbanas
Localidades Rurales
Límite Municipal
Fuentes de Información Cartográfica
Comisión Nacional de Áreas Naturales Protegidas
Instituto Nacional de Estadística y Geografía
Especificaciones Cartográficas
Sistema de Coordenadas: UTM
Zona 13 Norte
Cuadrícula: 3,000 m.
Elipsoide: GRS80
Datum Horizontal: ITRF92
Meridiano Central: -105
Escala : 1:100,000
Escala Gráfica: Kilómetros
Pico de Tancítaro
CONANP
Topográfico

Área de Protección de Flora y Fauna Pico de Tancítaro

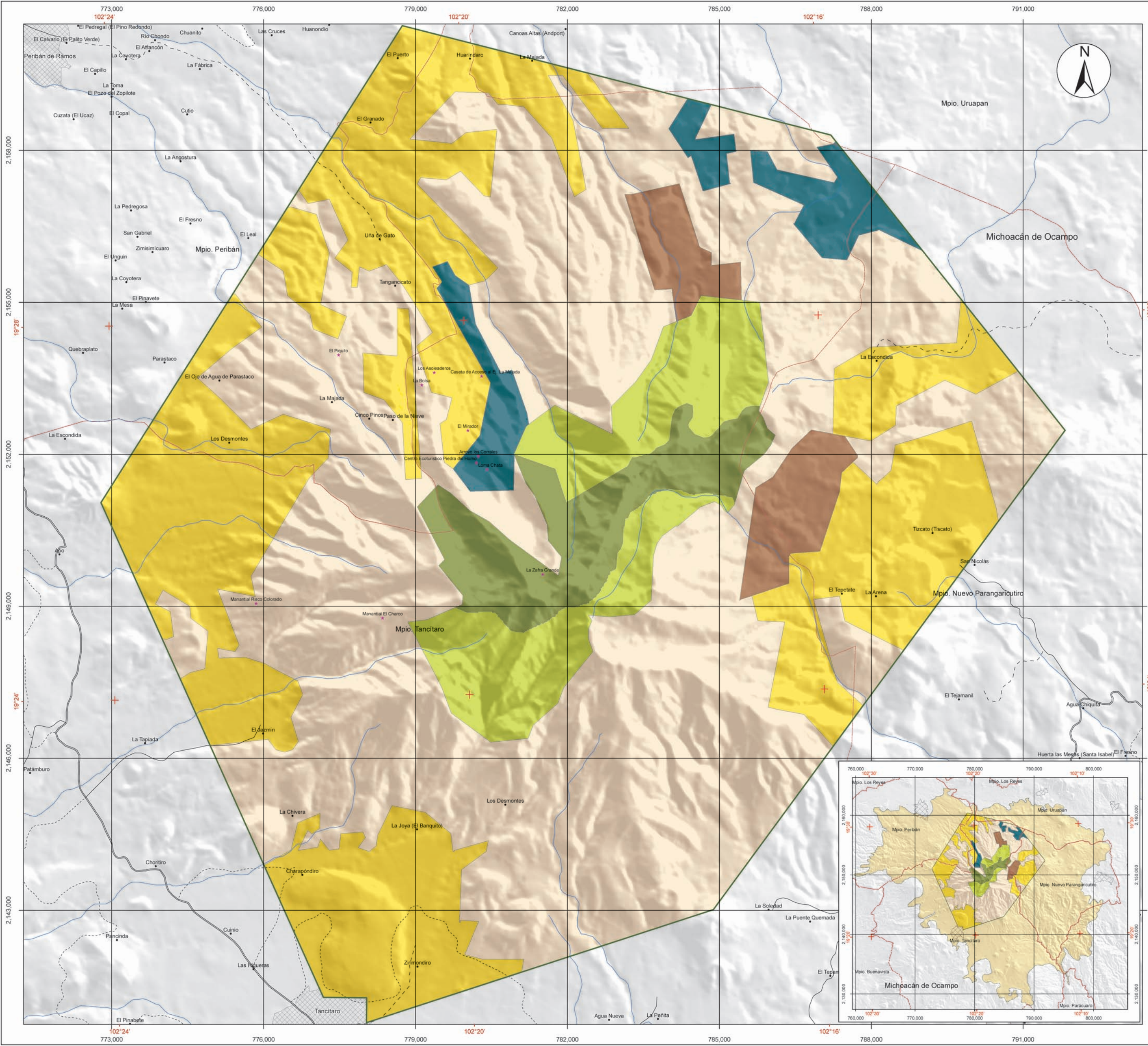

Macrolocalización

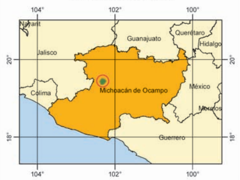

Imagen del Área

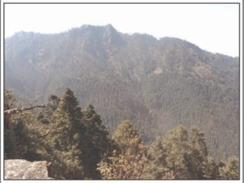

Simbología

Límite del Área Natural Protegida

Subzonificación

Zona Núcleo

Subzona de:

- Protección Pico de Tancítaro-Piedra del Horno
- Uso Restringido Cerro Tancítaro-La Culebra y La Zafra-Cerro San Pedro

Zona de Amortiguamiento

Subzona de:

- Aprovechamiento Sustentable de los Recursos Naturales Laderas Medias Circundantes del Tancítaro
- Aprovechamiento Sustentable de los Ecosistemas Laderas Bajas del Tancítaro.
- Uso Público Cerro Grande - Cenizas Volcánicas
- Recuperación Ladera Norte y Ladera Oriente

General

- Localidades Rurales
- Ríos
- Terracería
- Carretera Pavimentada
- Zona de Influencia
- Brecha
- Vereda
- Límite Municipal
- Localidades Urbanas
- Sitios Representativos

Fuentes de Información Cartográfica

Comisión Nacional de Áreas Naturales Protegidas
Instituto Nacional de Estadística y Geografía

Especificaciones Cartográficas

Sistema de Coordenadas: UTM
Zona 13 Norte
Cuadrícula: 3,000 metros.
Elipsoide: GRS80
Datum Horizontal: ITRF92
Meridiano Central: -105

Escala 1:50,000
Escala Gráfica: Kilómetros
0 0.5 1 2 3

CONANP
Comisión Nacional de Áreas Naturales Protegidas

Pico de Tancítaro
Área de Protección de Flora y Fauna

Subzonificación

PROGRAMA DE MANEJO

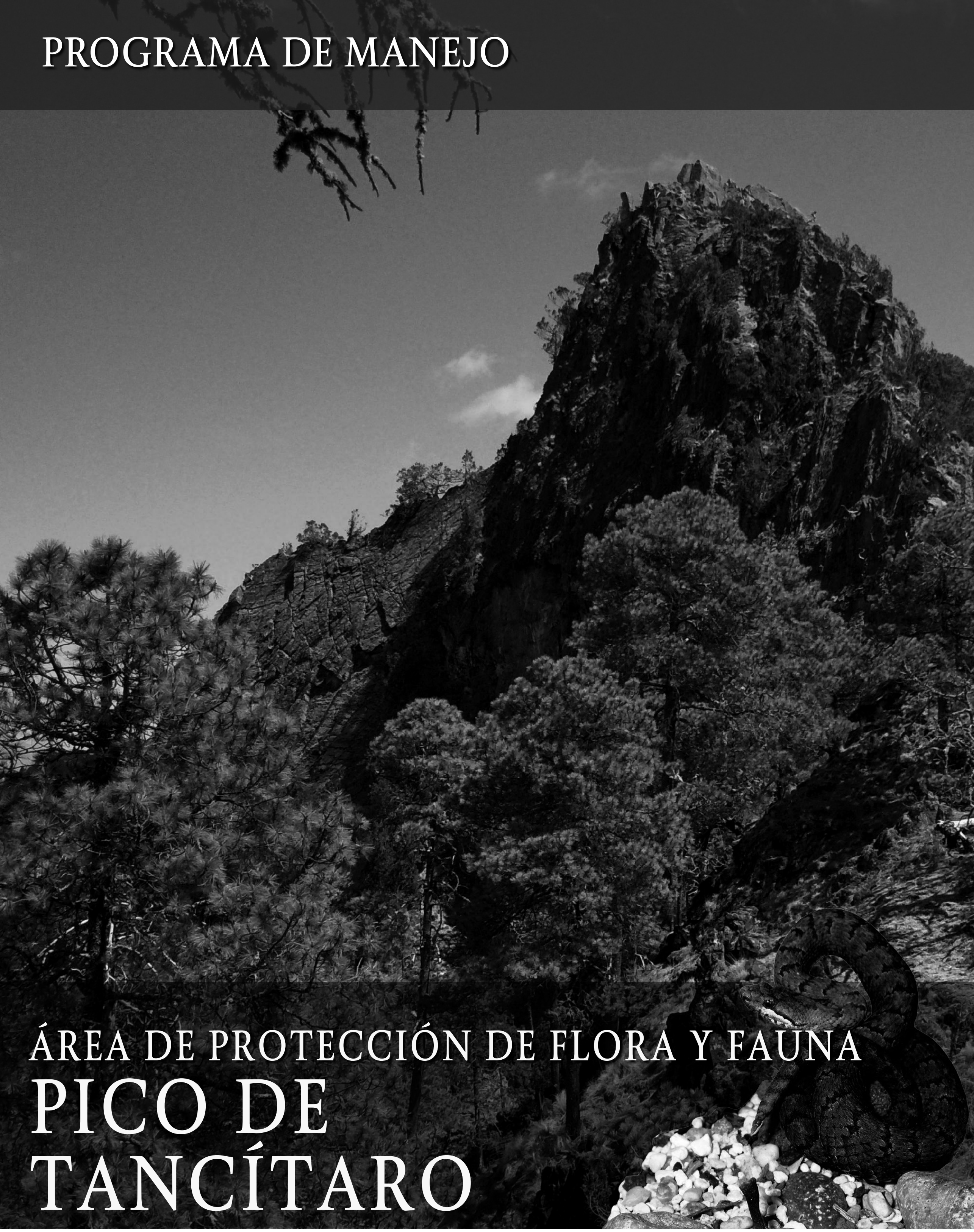

ÁREA DE PROTECCIÓN DE FLORA Y FAUNA
PICO DE TANCÍTARO

MÉXICO
GOBIERNO DE LA REPÚBLICA

ESTADOS UNIDOS MEXICANOS

SEMARNAT
SECRETARÍA DE
MEDIO AMBIENTE
Y RECURSOS NATURALES

CONANP
COMISIÓN NACIONAL
DE ÁREAS NATURALES
PROTEGIDAS

Pico de Tancítaro
Área de Protección de Flora y Fauna

Programa de Manejo Área de Protección de Flora y Fauna
Pico de Tancítaro

D. R. © **Secretaría de Medio Ambiente y Recursos Naturales**
Blvd. Adolfo Ruiz Cortines No. 4209, Col. Jardines en la Montaña, Tlalpan
C.P. 14210, México, D.F.
www.semarnat.gob.mx

Comisión Nacional de Áreas Naturales Protegidas
Camino al Ajusco No. 200, Col. Jardines en la Montaña, Tlalpan
C.P. 14210, México, D. F.
www.conanp.gob.mx
info@conanp.gob.mx

Primera edición: octubre de 2014

Impreso y hecho en México / *Printed and bound in Mexico.*

PRESENTACIÓN

Las comunidades que vivimos en la región del Pico de Tancítaro siempre hemos estado ligadas al uso, manejo y aprovechamiento de los recursos forestales, y con ello al desarrollo de nuestra gente y tradiciones.

Con la erupción del Volcán Paricutín no solo se pierde el poblado de San Juan Parangaricutiro, sino que también se pierden bosques y áreas de cultivo, afectando entre otras cosas, la base productiva de la comunidad.

A partir de la fundación del nuevo pueblo, al que se le llamó Nuevo San Juan Parangaricutiro, la comunidad retomó sus actividades de aprovechamiento del bosque, como base para su desarrollo. La comunidad hacía el aprovechamiento de recursos como la resina, pero transitamos a un manejo integral del recurso forestal, con una base sólida de desarrollo comunitario y organizacional. Actualmente nuestra empresa forestal es reconocida a nivel nacional e internacional, por ser un ejemplo del manejo forestal comunitario, en el que la sustentabilidad y el compromiso comunitario y social nos caracterizan.

De manera paradójica, parte de nuestros terrenos se encuentran al interior del Área Natural Protegida del Pico de Tancítaro, cuyos bosques no se podían aprovechar cuando su categoría era la de Parque Nacional.

Pese a ello, llevamos a cabo acciones de rescate de tierras agrícolas, reforestamos y cuidamos los recursos naturales. Nuestra comunidad reconoció el valor del Área Natural Protegida, pero también gestionamos por un cambio en su categoría que nos permitiera ampliar nuestro modelo de buen manejo de los recursos forestales y que al mismo tiempo posibilitara la protección de los recursos naturales.

Finalmente, en años recientes se cambió la figura de Parque Nacional por

la de Área de Protección de Flora y Fauna, lo que abrió las posibilidades de hacer uso de nuestros recursos naturales, bajo una visión sustentable y en estricto apego a las leyes y reglamentos. El presente Programa de Manejo refleja el esfuerzo por conservar y proteger los bosques que la comunidad tiene al interior del Área Natural Protegida, así como la oportunidad que se presenta con el nuevo esquema de protección para su uso racional.

Por todo lo anterior, estamos convencidos de que la conservación de los recursos naturales no es un obstáculo para el desarrollo de las comunidades, sino la base para mejorar las condiciones de vida a través del uso y manejo racional del Área.

El Comisariado de Bienes Comunales de la comunidad Indígena de
Nuevo San Juan Parangaricutiro, Michoacán.

C. Ambrosio Ruiz Soto	C. Felipe Aguilar Gómez	C. José Espinoza López
Presidente	Secretario	Tesorero

CONTENIDO

PRESENTACIÓN 3

1. INTRODUCCIÓN 11
   Antecedentes del Área Natural Protegida, en el contexto nacional, regional y local 12
      Contexto nacional 12
      Contexto regional 13
      Contexto local 15

2. OBJETIVOS DEL ÁREA NATURAL PROTEGIDA 17
   Objetivo general 17
   Objetivos específicos 17

3. OBJETIVOS DEL PROGRAMA DE MANEJO 19
   Objetivo general 19
   Objetivos específicos 19

4. DESCRIPCIÓN DEL ÁREA NATURAL PROTEGIDA. 21
   Localización y límites 21
   Características físico-geográficas 23
      Geología. 23
      Geomorfología y suelos 25
      Geomorfología 26
      Clima. 27
      Hidrología. 28
      Perturbaciones 29

Características biológicas 30
Vegetación 30
Fauna 35
Servicios ambientales 42
Contextos histórico, cultural y arqueológico 42
Historia del área 42
Cultura 43
Contextos demográfico y económico 44
Contexto demográfico 44
Contexto socioeconómico 46
Contexto social 47
Vocación natural del suelo 47
Análisis de la situación de la tenencia de la tierra 49
Normas Oficiales Mexicanas aplicables a las actividades a que esté sujeta el Área Natural Protegida 50

5. DIAGNÓSTICO Y PROBLEMÁTICA DE LA SITUACIÓN AMBIENTAL 53
Ecosistémico 53
Demográfico y socioeconómico 57
Presencia y coordinación institucional 58

6. SUBPROGRAMAS DE CONSERVACIÓN 59
Subprograma de protección 60
Objetivo general 60
Estrategias 60
Componente de mantenimiento de regímenes de perturbación y procesos ecológicos a gran escala 60
Objetivo específico 61
Meta y resultado esperado 61
Componente de prevención, control y combate de incendios y contingencias ambientales 61
Objetivo específico 61
Metas y resultados esperados 61
Componente de preservación e integridad de Zonas Núcleo y áreas frágiles y sensibles 62
Objetivo específico 62
Meta y resultado esperado 63
Componente de protección contra especies exóticas invasoras y control de especies y poblaciones que se tornen perjudiciales 63
Objetivos específicos 63
Metas y resultados esperados 63
Componente de mitigación y adaptación al cambio climático 65
Objetivo específico 65
Metas y resultados esperados 65

Componente de inspección y vigilancia 67
Objetivos específicos. 67
Metas y resultados esperados 67
Subprograma de manejo 68
Objetivo general. 68
Estrategias. 69
Componente de actividades productivas alternativas y tradicionales 69
Objetivo específico 69
Metas y resultados esperados 69
Componente de manejo y uso sustentable de agroecosistemas y ganadería 70
Objetivo específico 70
Metas y resultados esperados 70
Componente de manejo y uso sustentable de ecosistemas terrestres y recursos forestales 71
Objetivo específico 71
Meta y resultado esperado 71
Componente de manejo y uso sustentable de vida silvestre. 72
Objetivo específico 72
Metas y resultados esperados 72
Componente de mantenimiento de servicios ambientales 73
Objetivo específico 74
Metas y resultados esperados 74
Componente de turismo, uso público y recreación al aire libre. 74
Objetivo específico 75
Meta y resultado esperado 75
Subprograma de restauración 75
Objetivo general. 76
Estrategias. 76
Componente de conectividad y ecología del paisaje. 76
Objetivo específico 76
Meta y resultado esperado 76
Componente de recuperación de especies en riesgo 77
Objetivo específico 77
Meta y resultado esperado 77
Componente de conservación del agua y el suelo 78
Objetivo específico 78
Meta y resultado esperado 78
Componente de restauración de ecosistemas 79
Objetivo específico 79
Meta y resultado esperado 79
Subprograma de conocimiento 80
Objetivo general. 80
Estrategias. 80

Componente de fomento a la investigación 81
Objetivo específico 81
Meta y resultado esperado 81
Componente de inventarios y monitoreo ambiental y socioeconómico 82
Objetivos específicos. 82
Metas y resultados esperados 82
Componente de sistemas de información 83
Objetivo específico 83
Meta y resultado esperado 83
Subprograma de cultura. 83
Objetivo general. 84
Estrategias. 84
Componente de fomento a la educación y cultura para la conservación 84
Objetivo específico 84
Meta y resultado esperado 84
Componente de capacitación para el desarrollo sostenible. 85
Objetivo específico 85
Meta y resultado esperado 85
Componente de comunicación, difusión e interpretación ambiental 86
Objetivo específico 86
Meta y resultado esperado 86
Subprograma de gestión 87
Objetivo general 87
Estrategias 87
Componente de administración y operación. 88
Objetivo específico 88
Meta y resultado esperado 88
Componente de protección civil y mitigación de riesgos 88
Objetivo específico 89
Meta y resultado esperado 89
Componente de infraestructura, señalización y obra pública 89
Objetivo específico 89
Meta y resultado esperado 89
Componente de recursos humanos y profesionalización 90
Objetivo específico 90
Metas y resultados esperados 90

7. ORDENAMIENTO ECOLÓGICO Y ZONIFICACIÓN 91
Ordenamiento ecológico 91
Zonificación y subzonificación 91
Criterios de subzonificación. 91
Metodología 92
Subzonas y políticas de manejo 92
Subzona de Protección Pico de Tancítaro-Piedra del Horno. 93

Subzona de Uso Restringido Cerro Tancítaro-La Culebra y La Zafra-Cerro San Pedro . . . 97
Subzona de Aprovechamiento Sustentable de los Recursos Naturales Laderas Medias Circundantes del Tancítaro . . . 99
Subzona de Aprovechamiento Sustentable de los Ecosistemas Laderas Bajas del Tancítaro . . . 103
Subzona de Uso Público Cerro Grande-Cenizas Volcánicas . . . 105
Subzona de Recuperación Ladera Norte y Ladera Oriente . . . 107
Zona de Influencia . . . 110

8. REGLAS ADMINISTRATIVAS . . . 113
Capítulo I. Disposiciones generales . . . 113
Capítulo II. De las autorizaciones, concesiones y avisos . . . 116
Capítulo III. De los prestadores de servicios turísticos . . . 118
Capítulo IV. De los visitantes . . . 119
Capítulo V. De la investigación científica . . . 119
Capítulo VI. De los usos y aprovechamientos . . . 120
Capítulo VII. De la subzonificación . . . 121
Capítulo VIII. De las actividades prohibidas . . . 122
Capítulo IX. De la inspección y vigilancia . . . 122
Capítulo X. De las sanciones y recursos . . . 123

9. PROGRAMA OPERATIVO ANUAL . . . 125
Metodología . . . 125
Características del POA . . . 126
Proceso de definición y calendarización . . . 126
Seguimiento y evaluación del Programa Operativo Anual . . . 127

10. EVALUACIÓN DE LA EFECTIVIDAD DEL MANEJO . . . 129
Proceso de evaluación . . . 129

11. BIBLIOGRAFÍA . . . 131

11. ANEXOS . . . 141

PARTICIPACIÓN . . . 189

1. INTRODUCCIÓN

El Área de Protección de Flora y Fauna Pico de Tancítaro (APFFPT) ubicada en los municipios de Tancítaro, Peribán de Ramos, Nuevo Parangaricutiro y Uruapan, en el estado de Michoacán, consta de una superficie total de 23 mil 405-92-09.55 hectáreas, establecida mediante Decreto publicado en el *Diario Oficial de la Federación* el 19 de agosto de 2009.

En ella se encuentran especies endémicas y en alguna categoría de riesgo según la Norma Oficial Mexicana Nom-059-Semarnat-2010, Protección ambiental-Especies nativas de México de flora y fauna silvestres-Categorías de riesgo y especificaciones para su inclusión, exclusión o cambio-Lista de especies en riesgo. Presenta una gran variabilidad altitudinal que va de los dos mil metros a casi cuatro mil metros; el Pico de Tancítaro como sistema hidrológico cuenta con 16 cuencas, las cuales constituyen la base del desarrollo de al menos 39 mil 783 habitantes en 81 poblaciones y comunidades que se dedican al cultivo de aguacate, durazno, manzana y pera, principalmente, así como una variabilidad florística y faunística, por lo que este Programa de Manejo busca conducir de manera eficiente la administración y operación del Área Natural Protegida (Anp), orientándola hacia la conservación de los ecosistemas, su biodiversidad y el aprovechamiento sustentable de sus recursos naturales.

El Programa de Manejo del Área de Protección de Flora y Fauna Pico de Tancítaro se constituye como un instrumento de planeación y regulación, mediante el cual se exponen los objetivos del Área Natural Protegida y se describe su situación general desde los puntos de vista geográfico, físico y biológico, de su contexto arqueológico, histórico, cultural y paisajístico; así como de su entorno demográfico, económico y social, de uso y administrativo. Con base en todo ello, el Programa de Manejo presenta un

diagnóstico general de la problemática y potencialidades del Área de Protección de Flora y Fauna Pico de Tancítaro desde la perspectiva ambiental, económica y social, teniendo como finalidad la planificación y programación de acciones para la conservación y aprovechamiento sustentable de los recursos naturales por parte de sus habitantes y los de la Zona de Influencia, visitantes y usuarios (as) potenciales del Área Natural Protegida.

En los diferentes subprogramas que componen este documento se plantea abordar la problemática de manera global, bajo las siguientes seis líneas estratégicas: Protección, Manejo, Restauración, Conocimiento, Cultura y Gestión, estableciéndose los objetivos y estrategias de manejo para cada uno. A su vez, los Subprogramas tienen Componentes que plantean objetivos específicos, así como actividades y acciones a desarrollar por parte de la Dirección del Área, a fin de cumplir los objetivos de cada componente en los plazos programados.

En el capítulo de Ordenamiento Ecológico y Zonificación, el Programa de Manejo ubica unidades geográficas, que por sus características de uso y conservación están sujetas a políticas de manejo distintas, denominadas subzonas de conformidad con la Ley General del Equilibrio Ecológico y la Protección al Ambiente. Se prevén las actividades permitidas y no permitidas para cada una de ellas, en concordancia con el apartado denominado Reglas Administrativas, a las que deberán sujetarse las obras y actividades que se realicen en el Área, de conformidad con las disposiciones jurídicas aplicables.

En el capítulo siguiente se ofrece una guía para la elaboración, calendarización, seguimiento y evaluación del Programa Operativo Anual (POA) del Área, que con fundamento en las actividades y acciones plasmadas en los Subprogramas y Componentes deberá fungir como el instrumento de planeación a corto plazo, a través del cual se expresan los objetivos y metas a alcanzar en un año; y, en el apartado Evaluación de la Efectividad se establece el proceso de evaluación del presente Programa de Manejo, a fin de que éste sea revisado en cinco años.

Además, contiene varios anexos a los que el propio texto hace referencia, entre los que se encuentran los listados de flora y fauna del Área, así como la bibliografía consultada para su integración.

Antecedentes del Área Natural Protegida, en el contexto nacional, regional y local

Contexto nacional

Las Áreas Prioritarias Terrestres son territorios inscritos en el Programa Regiones Prioritarias para la Conservación de la Biodiversidad de la Comisión Nacional para el Uso y Conocimiento de la Biodiversidad (Conabio). Dicho Programa se aboca a detectar áreas, cuyas características físicas y bióticas favorecen condiciones particularmente importantes desde la perspectiva de su biodiversidad.

Para ello se determinan unidades estables desde el punto de vista ambiental que se localicen dentro del continente

del territorio nacional, en las que exista una riqueza ecosistémica y específica comparativamente mayor que en el resto del país, además de una integridad ecológica funcional significativa que permita a dichas unidades la posibilidad real de ser conservadas.

Se considera una Región Prioritaria para la Conservación, ya que incluye especies endémicas; un ejemplo de un género pancrónico endémico (fósil viviente) de esta región es *Zygogeomys trichopus,* una tuza muy poco conocida aún y en peligro de extinción. Los vertebrados en general son peculiares. En las partes altas del Anp existen bosques de pino de altura, oyameles, encinos y pinos de diversas especies, así como pastizales y agricultura de temporal en las zonas.

La región en la que se encuentra el Pico de Tancítaro ha sido reconocida a través de diferentes clasificaciones y esquemas que indican su importancia como parte de la biodiversidad mexicana. La Conabio la señaló como una Región Terrestre Prioritaria (RTP-114-Tancítaro) con una extensión de 407 mil 151 kilómetros cuadrados, por ser la única zona bien conservada entre Colima y la Sierra Chincua, en Michoacán, por su alto valor para la conservación por la presencia de endemismos de reptiles, aves y mamíferos, y como centro de origen y diversificación de especies (Arriaga *et al*., 2002; Conanp, 2008). También corresponde un área de importancia para la conservación de las aves (AICA C-5) (Villalón *et al.,* 2000, en Arizmendi y Márquez Valdelamar, 2000).

Finalmente podemos decir que el estado de Michoacán destaca por el cultivo de aguacate de la variedad Hass, tanto por su extensión de 96 mil 764 hectáreas como por su producción anual de un millón 3 mil 450 toneladas (SIAP-SAGARPA, 2006), que representa el 29 por ciento del total de la superficie plantada a nivel nacional y el 36.5 por ciento de la producción mundial, ubicándolo como el principal productor.

Contexto regional

Debido a la importancia paisajística e hidrológica del área conocida como Pico de Tancítaro, en el estado de Michoacán, el Presidente de la República, Lázaro Cárdenas del Río, publicó en el *Diario Oficial de la Federación* el 27 de julio de 1940 un Decreto en el que declara como Parque Nacional Pico de Tancítaro una amplia área de terreno en torno a la montaña más alta del estado de Michoacán.

Una nueva iniciativa se realizó en 2004 y 2005, integrando a la mayoría de las instancias del sector ambiental en el estado de Michoacán, tanto del sector público como del privado. El 19 de agosto de 2009, en un proceso abierto y participativo, se publicó en el *Diario Oficial de la Federación* el Decreto mediante el cual se declara Área Natural Protegida, con el carácter de Área de Protección de Flora y Fauna la región conocida como Pico de Tancítaro, la cual se estableció por la necesidad de proteger la mencionada región bajo esquemas que garanticen la preservación de los elementos naturales que la componen.

Con el carácter de Área de Protección de Flora y Fauna (APFF), es la primera área en ser decretada en esta categoría para el estado de Michoacán y es considerada de gran importancia para la conservación por ser asiento de ecosistemas con características especiales en cuanto a la biodiversidad que soporta en su superficie (Conabio, 1999; Ordoñez y Flores Villela, 1995; Flores y Gerez, 1994) y por su importancia hidrológica (Bocco *et al.*, 1999; Fuentes, 2000).

El APFF Pico de Tancítaro cumple con las condiciones necesarias para considerar a un territorio como de alta importancia biogeográfica a nivel regional:

- Constituye una extensa superficie de captación de agua, conformada por 16 cuencas hidrológicas, lo que confiere una gran importancia dentro del ciclo de captación de agua y recarga de acuíferos.

- La región del Pico de Tancítaro es un corredor biológico montañoso, en el que existen distintos tipos de vegetación, como los bosques templados de *Pinus, Abies* y *Quercus;* asimismo, pastizales y vegetación secundaria, que contienen áreas en buen estado de conservación, los cuales constituyen el hábitat de diversas especies de flora y fauna endémicas.

- Las comunidades vegetales que se distribuyen de manera natural en el área constituyen el hábitat de numerosas especies silvestres y proveen alimento, refugio y sitios de reproducción a las especies de fauna silvestre.

- En el área se encuentran especies enlistadas en la Norma Oficial Mexicana Nom-059-Semarnat-2010, Protección ambiental-Especies nativas de México de flora y fauna silvestres-Categorías de riesgo y especificaciones para su inclusión, exclusión o cambio-Lista de especies en riesgo.

- Por sus características hidrológicas, topográficas, edáficas, climáticas y biológicas, el área Pico de Tancítaro constituye un sitio con alto valor biológico al ser el centro de origen y diversificación de especies con presencia de endemismos de reptiles, aves y mamíferos.

- La riqueza biológica y ecológica del área le confiere características y elementos que se traducen en un conjunto de bienes y servicios ambientales para las poblaciones aledañas y provee de agua a las comunidades dedicadas al cultivo de aguacate, principalmente.

Además, si se toma en cuenta la superficie boscosa que aún posee, entonces se puede afirmar que el Pico de Tancítaro y sus alrededores conforman un sistema de cobertura importante, por ejemplo, para el secuestro de carbono y la regulación del clima regional, permitiendo así la estabilidad de los diferentes procesos biogeoquímicos del paisaje (Fuentes, 2000).

Finalmente, es importante mencionar que el Área Natural Protegida y sus alrededores constituyen una de las áreas más importantes del país en la producción de aguacate de exportación,

incluyéndose en la zona conocida como "el corredor aguacatero" o franja aguacatera de Michoacán (Aguilera Montañez y Salazar García, 1991; Torres y Bocco, 1999; Gutiérrez Contreras *et al.*, 2007; Echánove, 2008).

Contexto local

En el Área de Protección de Flora y Fauna algunos de los proyectos y estudios que se han desarrollado y que han ayudado a la conservación de los ecosistemas y su biodiversidad están relacionados con la captura de carbono, el ordenamiento ecológico local, los recursos hídricos, el apoyo para la reforestación, el manejo sustentable de ecosistemas, la legislación ambiental, el paisaje, la geomorfología y la geología, los Sistemas de Información Geográfica y los inventarios taxonómicos de aves, reptiles, anfibios y mamíferos.

Estos proyectos y estudios han sido realizados principalmente por investigadores de instituciones académicas, como el Centro de Investigaciones en Ecosistemas (CIEco) y el Centro de Investigaciones en Geografía Ambiental (CIGA), ambos de la Universidad Nacional Autónoma de México (UNAM), la Universidad Michoacana de San Nicolás de Hidalgo (UMSNH) y dependencias federales, como la Conabio, así como del gobierno estatal, quienes han contribuido de manera sustancial para enriquecer la información plasmada en el presente Programa de Manejo.

2. OBJETIVOS DEL ÁREA NATURAL PROTEGIDA

Objetivo general

Adoptar esquemas de conservación que armonicen las necesidades económicas y sociales de los pobladores con las necesidades de conservación de los elementos naturales, con la finalidad de salvaguardar la diversidad genética y biológica de las especies silvestres de las que depende la continuidad evolutiva, así como asegurar la preservación y el aprovechamiento sustentable de la biodiversidad que alberga el Área Natural Protegida.

Objetivos específicos

- Preservar los ambientes naturales del área y de los ecosistemas más frágiles para asegurar el equilibrio y la continuidad de los procesos evolutivos y ecológicos, por brindar bienes y servicios ambientales, como corredor biológico montañoso y por albergar especies de flora y fauna silvestres de importancia biológica.

- Conservar las especies de flora y fauna silvestres, las endémicas, las raras, las que se encuentran sujetas a alguna categoría de riesgo y las de importancia económica actual y potencial.

- Asegurar el aprovechamiento sustentable de los ecosistemas presentes en el Anp y sus elementos.

- Impulsar el desarrollo de las actividades de investigación científica y monitoreo ambiental y de la biodiversidad en el Anp.

- Proteger las zonas boscosas de las cuencas hidrológicas de la que forma parte y demás elementos naturales circundantes con los que se relaciona el ecosistema.

- Generar, rescatar y divulgar conocimientos, prácticas y tecnologías tradicionales o nuevas que permitan la preservación y el aprovechamiento sustentable y racional de la biodiversidad y los recursos naturales del Anp.

3. OBJETIVOS DEL PROGRAMA DE MANEJO

Objetivo general

Constituir el instrumento rector de planeación y regulación, el cual establece las actividades, acciones y lineamientos básicos para el manejo y la administración del Área Natural Protegida con el carácter de Área de Protección de Flora y Fauna Pico de Tancítaro.

Objetivos específicos

Protección: Favorecer la permanencia y conservación de la diversidad biológica del Área de Protección de Flora y Fauna Pico de Tancítaro, a través del establecimiento y promoción de un conjunto de políticas y medidas para mejorar el ambiente y controlar el deterioro de los ecosistemas.

Manejo: Establecer políticas, estrategias y programas, con el fin de determinar actividades y acciones orientadas al cumplimiento de los objetivos de conservación, protección, restauración, capacitación, educación, aprovechamiento sustentable de los recursos naturales y recreación del Área de Protección de Flora y Fauna Pico de Tancítaro, a través de proyectos alternativos y la promoción de actividades de desarrollo sustentable.

Restauración: Recuperar y restablecer las condiciones ecológicas previas a las modificaciones causadas por las actividades humanas o fenómenos naturales, permitiendo la continuidad de los procesos naturales en los ecosistemas del Área de Protección de Flora y Fauna Pico de Tancítaro.

Conocimiento: Generar, rescatar y divulgar conocimientos, prácticas y tecnologías tradicionales o nuevas que permitan la preservación, la toma de decisiones y el aprovechamiento sustentable de la biodiversidad del Área de Protección de Flora y Fauna Pico de Tancítaro.

Cultura: Difundir acciones de conservación del Área de Protección de Flora y Fauna Pico de Tancítaro,

propiciando la valoración de los servicios ambientales, mediante la difusión y educación para la conservación de la biodiversidad que contiene.

Gestión: Establecer las formas en que se organizará la administración del Área de Protección de Flora y Fauna Pico de Tancítaro y los mecanismos de participación de los tres órdenes de gobierno, de los individuos y comunidades aledañas a la misma, así como de todas aquellas personas, instituciones, grupos y organizaciones sociales interesadas en su conservación y aprovechamiento sustentable.

4. DESCRIPCIÓN DEL ÁREA NATURAL PROTEGIDA

Localización y límites

El Área de Protección de Flora y Fauna Pico de Tancítaro se localiza en el estado de Michoacán de Ocampo y se ubica en los municipios de Tancítaro, Peribán de Ramos, Uruapan y Nuevo Parangaricutiro, con una superficie de 23 mil 405-92-09.55 hectáreas, según su Decreto de creación. En el Cuadro 1 se muestra la superficie específica de cada uno de los municipios que forman parte del polígono. Las coordenadas extremas en la poligonal propuesta para el APFF Pico de Tancítaro son 19°31'09.83''-19°20'30.61'' latitud Norte y 102°13'14.34''-102°24'07.42'' longitud Oeste.

Cuadro 1. Superficies correspondientes a cada municipio dentro del APFF Pico de Tancítaro

**Municipio**	**Superficie en hectáreas dentro del Anp**
Tancítaro	17,428.724768
Peribán de Ramos	2,643.247960
Uruapan	204.890479
Nuevo Parangaricutiro	3,129.057748
**Total general**	**23,405.920955**

Figura 1. Ubicación general del Área de Protección de Flora y Fauna Pico de Tancítaro

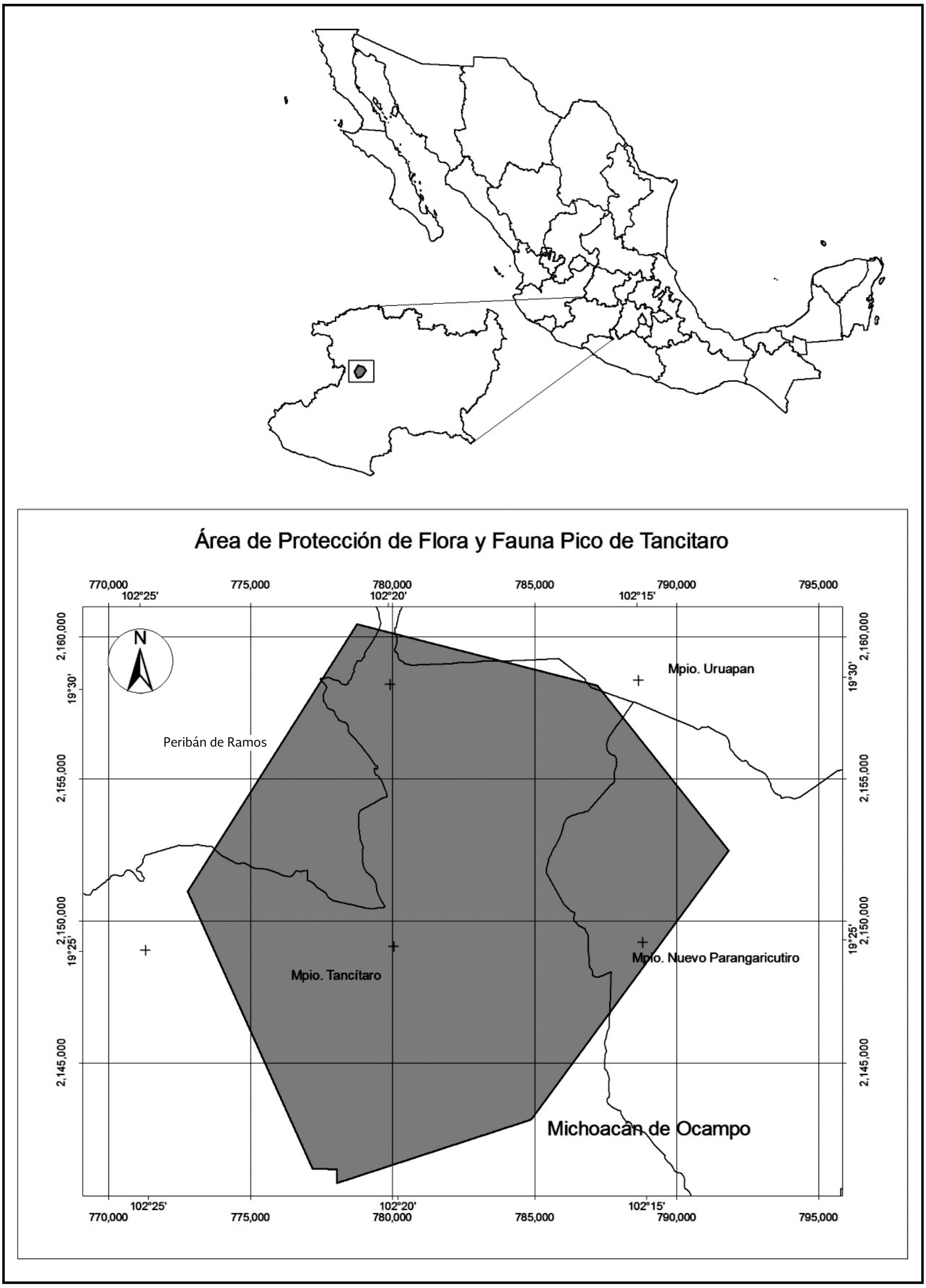

Características físico-geográficas

Geología

De acuerdo con Scattolin (1996) y Ban *et al.*, (1992), citado por Garduño *et al.*, (1999), el estratovolcán Tancítaro tiene una edad de alrededor de 500 mil años, lo que lo ubica como una estructura cuaternaria más o menos reciente.

El Área de Protección de Flora y Fauna Pico de Tancítaro presenta una serie de sistemas litoestratigráficos agrupados en un gran supersistema, denominado La Culebra, perteneciente a su vez, al denominado Distrito Volcánico del Tancítaro (DVT). Los sistemas que lo comprenden son el de Zacándaro, el de Piedra del Horno y Nuevo Parangaricutiro.

El supersistema La Culebra, de acuerdo a la morfología, los cambios de actividad volcánica y la ubicación de la zona de alimentación de los productos volcánicos, es la base para describir la geología general del Tancítaro (Scattolin, 1996).

Sistema Zacándaro. Es el sistema más antiguo de La Culebra; está delimitado por una superficie erosionada que la divide estratigráficamente de la formación Zumpimito, mientras que el límite superior estratigráfico es con el sistema Piedra del Horno, establecido a partir de cambios en la actividad volcánica. Gran parte de los productos y edificios volcánicos se localizan en el extremo noroccidental del Tancítaro, hallándose menos evidencias hacia el extremo suroriental.

Sistema Piedra del Horno. Este sistema aflora en la zona central del DVT y constituye el límite superior del Sistema Zacándaro, evidenciado por un cambio de actividad que significó la formación y desarrollo del estratovolcán del Tancítaro y sus correspondientes productos. El límite superior se identifica con la interrupción de dicha actividad y el nacimiento y desarrollo de volcanes de lava y conos monogenéticos.

Sistema Nuevo Parangaricutiro. La aparición de este sistema constituye el límite superior del sistema anterior y se relaciona con el cese de actividad del Tancítaro y el inicio y desarrollo de una serie de volcanes de lava y conos cineríticos; se subdivide en las unidades inferiores: Unidad Lomas La Tinaja, litosoma Tumbiscatillo, la Unidad Grande, litosoma La Uva, La Unidad Pancinda, litosoma Los Amoles, Unidad La Chimenea, litosoma Parastaco, litosoma Zirimóndiro, litosoma La Escondida y litosoma Paricutín, el cual constituye el edificio volcánico más joven y comprende a la Unidad Zapicho, la cual incluye la colada y las cenizas producto del Paricutín.

Por otra parte, en 1950 Segerstron mencionó que históricamente el Pico de Tancítaro se localiza en una porción del territorio michoacano en la que se encuentra el Paricutín; se estima que tiene aproximadamente dos millones de años y data de la época geológica del Pleistoceno al Plioceno.

La zona se distingue por una intensa actividad volcánica, por lo menos durante los últimos 500 mil años. Debido a su edad, el cerro Pico de Tancítaro se ha ido desgastando y su típica forma volcánica se perdió, por lo que ahora tiene la

forma de un pico rugoso con extensas y empinadas laderas de norte a sur.

A simple vista se aprecia un solo bloque montañoso en el área del Pico de Tancítaro, pero en realidad es una serie de cerros o picos que dificultan su acceso y visibilidad a la cumbre. La altura más baja se localiza en el Pueblo de Tancítaro, a 2 mil 100 metros sobre el nivel del mar (García, 1998). Las principales elevaciones del área se presentan en el Cuadro 2.

Cuadro 2. Elevaciones en el APFF Pico de Tancítaro

Nombre	Altitud (metros sobre el nivel del mar)
Cerro Pico de Tancítaro	3,860
Cerro Piedra del Horno	3,640
Cerro El Arco	3,640
Cerro La Zafra	3,560
Cerro La Cruz	3,550
Cerro Tangarico	3,520
Cerro San Pedro	3,360
Cerro La Chimenea	3,160
Cerro El Cebo	3,080
Cerro Prieto	3,040
Cerro El Tepetate	2,880
Cerro Llacuaro	2,860
Cerro La Alberca	2,800
Cerro La Soledad	2,700
Cerro del Estudiante	2,520
Cerro La Cantera	2,520
Cerro La Cruz (Apo)	2,380

El área está clasificada dentro del denominado Eje Neovolcánico Transversal, el cual constituye una franja volcánica del cenozoico superior, que se extiende transversalmente a través de México, desde el Golfo de México hasta la Costa del Pacífico.

Como se mencionó anteriormente, el área del Pico de Tancítaro presenta diversas elevaciones, lo cual es relevante porque debido a esto se presentan varios tipos de vegetación, clima y, en consecuencia, un variado número de especies de flora y fauna, algunas de ellas endémicas.

Cuadro 3. Geología del Distrito Volcánico de Tancítaro, según Scattolin (1996, tomado de Fuentes, 2000)

Unidad sistemática		Unidad litosomática	Rocas predominantes	Edad (millones de años)
**Supersistema**	**Sistema**			
La Culebra	Nuevo Parangaricutiro	Paricutín<br>La Escondida<br>Zirimóndiro<br>Parastaco<br>Los Amoles<br>La Uva<br>Tumbiscatío	Volcanes de lava y conos cineríticos.<br>Lavas y bloques andesítico-basálticas, andesíticas y productos volcánicos de tipo basáltico ricos en olivino	1943 d.C.
	Piedra del Horno	Tancítaro	Andesita porfírica y dacita escoriácea	
	Zacándaro	La Sidra	Productos Andesítico-basálticos	0.55 + 0.06

Geomorfología y suelos

Tipos de suelo

Aunque hay una gran variabilidad de tipos y unidades de suelo, predominan aquellos que provienen de sustratos volcánicos, por lo que los andosoles úmbricos son los suelos más conspicuos y ocupan el 95 por ciento de la superficie del APFF Pico de Tancítaro; el porcentaje restante (cinco por ciento) lo ocupan el resto de los tipos y unidades de suelo.

Andosol úmbrico (ANu): según la clasificación de la FAO-UNESCO en 1989, el andosol es un suelo que presenta una porción relativamente alta de hierro y aluminio en la fracción de tierra fina y una alta retención de fosfatos hasta una profundidad de 35 centímetros, como mínimo. El subtipo úmbrico tiene una consistencia untuosa y una textura franco limosa o muy fina y no se satura de agua en ninguna época del año; posee un horizonte A no muy duro cuando se seca, con un grado de saturación menor de 50 por ciento y con relativamente alto nivel de contenido de carbono orgánico y un horizonte B de alteración, color claro y bajo contenido de materia orgánica (Arriaga *et al.,* 2000).

Después de los andosoles se encuentran representados los regosoles; su distribución, igual que la del leptosol, está vinculada al establecimiento de cenizas volcánicas y efusiones recientes de material volcánico. Son suelos someros con alta rocosidad y muy susceptibles a la erosión.

El tercer tipo de suelo en importancia es el luvisol; se localiza hacia el sureste del Anp y tiene relación con zonas de mayor desarrollo, afectadas probablemente por acontecimientos geológicos paroxismales que afectaron al Tancítaro hace miles de años (Víctor Garduño, *com. pers.)* y que provocaron avalanchas de material hacia la barranca La Culebra, donde fue depositando el material parental de

este tipo de suelo. Por su antigüedad, la pendiente y la composición del material de esta zona están afectadas por procesos de erosión hídrica y gravitacional.

El leptosol se asocia con andosoles y regosoles, correspondiendo a las zonas de 35 lavas recientes o de malpaís y hacia el suroeste de Tancítaro. Los suelos son pedregosos, con alta rocosidad y muy someros.

Por último, el cambisol es el suelo menos abundante y se presenta en la zona de Peribán de Ramos y cerca del Cerro La Chimenea. Son suelos de buen desarrollo, dedicados generalmente a la agricultura, específicamente, en los límites del Área Natural Protegida. Predominan las asociaciones de suelo de andosol con leptosol, sobre todo en el macizo del estratovolcán, evidenciando suelos típicamente forestales y susceptibles a la erosión en condiciones de deforestación (Bocco y Mass, 2001-2003).

Asimismo, se presentan asociaciones de andosol húmico y ócrico en la zona de piedemonte. Estos suelos presentan condiciones adecuadas para el cultivo y en esta zona se presentan los cultivos permanentes de aguacate y durazno (Velázquez, 2004).

El suelo presenta procesos de compactación predominante en altitudes entre 3 mil 200 y 3 mil 600 metros sobre el nivel del mar. También se observan los efectos de la erosión laminar y una incipiente erosión concentrada (surcos y cárcavas), debido sobre todo a cambios en la estructura de la cobertura vegetal, el uso del suelo, la tala y el excesivo pastoreo. Esta situación es observable en prácticamente todo el territorio (Velázquez, 2004).

Cuadro 4. Tipos de suelo y localización dentro del APFF Pico de Tancítaro

Suelo	Localización dentro del área
Andosoles	Los más conspicuos, distribución vinculada al establecimiento de cenizas volcánicas, ocupando casi la totalidad del ANP
Regosoles	Distribución al establecimiento de cenizas volcánicas, en la porción noreste del ANP
Luvisoles	Al sureste del área
Leptosoles	Al suroeste del área
Cambisoles	En la zona de Peribán de Ramos y cerca del Cerro La Chimenea

Geomorfología

De acuerdo con Bocco *et al.*, (2001) y la regionalización geomorfológica del estado de Michoacán, los paisajes geomorfológicos característicos del área son las sierras y los piedemonte.

Los piedemonte, de acuerdo con Bocco, son "...unidades transicionales entre un relieve positivo y la planicie de nivel de base local. Presenta pendientes menores a 10° y la amplitud del relieve puede variar considerablemente, de decenas a cientos de metros. Dentro de las elevaciones se diferencian cuatro

niveles: colinas, lomeríos bajos, lomeríos altos y sierra”.

Existen tres tipos de planicies en el área: interlávica, aluvial y aluvial interlávica. Estas unidades acumulativas presentan en algunas zonas (al noroeste y al norte) evidencias claras de erosión; sobre todo en aquellas planicies formadas por cenizas, ya que el material tan débil que la forma es muy susceptible a la erosión hídrica. Por otra parte, la pendiente (menos de 8° de inclinación) favorece el establecimiento de la mayor parte de los cultivos agrícolas, lo que puede ocasionar el agotamiento de algunos suelos (Fuentes, 2000).

El Sistema Tancítaro incluye todas las laderas y cimas del estratovolcán, que también comprende las unidades lávicas de tipo andesítico dominantes. Debido a la configuración geomorfológica de éstas, el sistema está dominado por pendientes de alto grado, que van desde 15° de inclinación hasta más de 30°, presentándose con frecuencia escarpes rocosos ubicados generalmente por encima de los 3 mil 500 metros sobre el nivel del mar (Fuentes, 2000).

Las estructuras volcánicas monogenéticas están constituidas por conos volcánicos de escoria y cenizas, y domos lávicos. Su principal característica es la pendiente que presentan (mayor a 20°), aunque una gran cantidad presentan pendientes muy superiores debido a su juventud y origen. Algunas estructuras han desarrollado valles en “V” que forman barrancas, debido sobre todo a condiciones estructurales, pero acentuadas por la deforestación y el cambio de uso del suelo (Fuentes, 2000).

Estas estructuras son importantes porque marcan el rompimiento del paisaje, conformando un rosario de elevaciones que rodean el estratovolcán y que seguramente son la clave que explica la actividad volcánica posterior al Tancítaro (Fuentes, 2000).

En cuanto a los derrames lávicos, las laderas por lo general son muy inclinadas y en ocasiones hasta abruptas (más de 30° de pendiente), con superficies cumbrales amplias y de escasa pendiente (menos de 8° de inclinación). La importancia de estas unidades estriba en que algunos elementos son muy recientes, dando como resultado zonas de captación de agua (áreas de malpaís). Las laderas de derrames lávicos poseen pendientes mucho más bajas en lo general, incluso forman lavas escalonadas donde se practica la agricultura de temporal y permanente.

Clima

De acuerdo con la carta de climas elaborada por la Conabio (1998), con base en la clasificación climática de Köeppen, modificado por García E. (1984), los climas en la zona de estudio siguen un patrón altitudinal influidos en su humedad por la depresión del Balsas hacia las laderas este, suroeste y sur del Tancítaro y por el fenómeno de continentalidad en la ladera norte.

El clima predominante en el área es del tipo cálido subhúmedo con abundantes lluvias en verano; de acuerdo con la clasificación de Köppen modificada por García, E. (1984), es del tipo C (m)(w) (Fuentes, 2000; Zamora, 2006). Presenta una temperatura media anual que varía

de 8 a 18 °C y los extremos oscilan entre los 6.5 y los 22 °C. La precipitación pluvial va de 800 a mil 200 milímetros cúbicos, con presencia de fuertes lluvias en los meses de verano.

Para el Área de Pico de Tancítaro se observa una gradación climática que a continuación se menciona y que comprende un total de ocho tipos climáticos.

A altitudes menores a mil metros sobre el nivel del mar y fuera del ANP, aunque limitando con ella, en una pequeña franja se presentan climas Aw0 y Aw1. Son climas cálido subhúmedos con lluvias en verano. Su característica es la presencia de precipitaciones por encima de los 800 milímetros anuales que se presentan en verano y una variación de la temperatura poco significativa; las comunidades vegetales características son selvas bajas y medianas subcaducifolias. El clima Aw1 es ligeramente más húmedo que el Aw0.

A partir de los mil metros sobre el nivel del mar, aproximadamente, y coincidiendo con cambios en el relieve, el clima se vuelve menos cálido, presentándose climas del tipo (A)C(w1) y (A)C(w2); es decir, semicálidos subhúmedos con lluvias en verano.

Al consultar los valores mensuales y anuales de las estaciones correspondientes, se puede observar que estos climas se caracterizan por una escasa oscilación térmica durante el día, donde las precipitaciones que se presentan pueden ser de hasta mil 500 milímetros anuales.

Posteriormente, en altitudes superiores a los dos mil metros sobre el nivel del mar se observa la presencia de dos tipos climáticos del grupo de los templados: el C (w2) y el Cm o templado subhúmedo y templado húmedo con lluvias en verano, respectivamente. Los rangos de temperatura se ubican entre 12 °C y 18 °C y la precipitación puede llegar a los mil 500 milímetros anuales. El rango altitudinal de estos climas termina aproximadamente a los tres mil metros sobre el nivel del mar, donde las temperaturas son mucho más bajas. Las comunidades vegetales más conspicuas son los bosques mixtos, sobre todo de encino-pino, los de coníferas *(Abies* y *Pinus)* y el bosque mesófilo.

A partir de los tres mil metros sobre el nivel del mar, aproximadamente, los climas cambian al tipo Cb'(m), denominando el semifrío húmedo con verano fresco largo. Este clima domina las partes más altas del Pico de Tancítaro.

Las temperaturas anuales promedio van desde los 5 °C hasta los 12 °C en las cimas del Tancítaro. Las precipitaciones promedio se ubican entre 900 y mil milímetros anuales. En estos climas predominan las coníferas, pero también se presentan de manera localizada (en cañadas angostas) bosques de *Abies*. Cabe mencionar que en el caso del Tancítaro, la especie de pino predominante es el *Pinus hartwegii* para este tipo climático. El último tipo climático es el Cb'(w2), semifrío subhúmedo con verano fresco largo, diferenciándose del anterior por una menor precipitación, pero manteniendo las demás características. Este clima se localiza al noreste del Pico de Tancítaro y corona las cimas de los

cerros de Angahuan, enlazados con el Tancítaro por la cuenca que los comunica (Fuentes y Alvarado, 2006).

Hidrología

El Área de Protección de Flora y Fauna Pico de Tancítaro forma parte de la cuenca del Río Balsas a través de las subcuencas de los ríos Tepalcatepec y Cupatitzio.

En el Área Natural Protegida existen un sinnúmero de ríos intermitentes, tales como: al oeste Apo y Choritiro, y al sur y poniente Piedra azul, Las Tinajas, Tancítaro, La Gringa, El Fresnito, Rancho Nuevo, Tiscato, Las Amapolas, La Culebra y Charapóndiro, entre otros.

La región de Tancítaro, donde se encuentra el Anp, no está considerada como propicia para la presencia de manantiales y solamente se localizan dos de ellos de características no termales: Barranca Charapóndiro (del Agua), que suministra agua potable a las poblaciones de Zirimóndiro y Tancítaro, y La Zafra, que suministra agua potable al pueblo de Apo (García *et al.*, 1998).

Según los estudios realizados por Palacio (1986) y Lugo (1988), se estableció la red de drenaje y se obtuvieron 16 cuencas que corresponden al Pico de Tancítaro: la cuenca del Río Huandiestacato drena la mayor parte de los escurrimientos tanto del Volcán Paricutín como del sector noreste del Tancítaro, además de las cuencas de Zirimóndiro y El Chivo, localizadas hacia la porción suroccidental. Estas cuencas prácticamente no poseen escurrimientos permanentes debido al substrato geológico en el que se localizan, por lo que la red es poco densa pero de gran valor ambiental (Fuentes y Alvarado, 2006).

Otra zona de importancia hídrica corresponde a los escurrimientos que drenan hacia la porción occidental del Área Natural Protegida. Esta área involucra a las cuencas de Chuanito, Chondo, Cutio, Hoyicazuela, Apo, Rodada y Tancítaro (Fuentes y Alvarado, 2006).

Por las características de los suelos y de la misma vegetación, es una zona de mucha importancia en la recarga de acuíferos; esto se puede constatar al poniente del Área de Protección de Flora y Fauna Pico de Tancítaro, en las zonas más bajas en los Chorros del Varal y al oriente en las cercanías de Uruapan, en la barranca del río Cupatitzio.

En un estudio de Fuentes y Bocco (2003), sobre la dinámica y análisis regional del agua en la Región del Pico de Tancítaro, mencionan que no se tiene registro de poblaciones con problemas graves de escasez de agua; sin embargo, sí muestran problemas evidentes, como en la localidad La Escondida, ubicada dentro del Anp, así como varias localidades de la Zona de Influencia y pertenecientes al municipio de Peribán de Ramos. En la mayoría de los casos, los problemas provienen de una pésima o nula administración del recurso y del incipiente reflejo del deterioro ambiental a que se ha visto sometida la región.

Perturbaciones

Los fenómenos naturales ocurridos en el área son principalmente incendios forestales. En el Cuadro 5 se describen el número de incendios forestales y la

superficie afectada entre 2006 y 2010 en los municipios que abarcan el Área Natural Protegida y su Zona de Influencia, donde el municipio de Uruapan es el que ha tenido el mayor número y superficie afectada, sobre todo en 2006, que es cuando hubo una mayor afectación.

Dentro del Área Natural Protegida algunos de los incendios ocurridos son por causas naturales, principalmente en época de sequía, presentándose mayormente en la pequeña porción que abarca el municipio de Uruapan dentro del polígono del Área y en el municipio de Nuevo Parangaricutiro. En los municipios de Peribán de Ramos y Tancítaro los incendios forestales son provocados principalmente para el pastoreo del ganado.

Cuadro 5 . Superficie afectada por incendios forestales desde 2006 hasta 2010 dentro del Anp y su Zona de Influencia

Municipio	2006		2007		2008		2009		2010	
	No. incendios	Sup. afectada	No. incendios	Sup. afectada	No. incendios	Sup. afectada	No. incendios	Sup. afectada	No. incendios	Sup. afectada
Nuevo Parangaricutiro	1	3	4	34	3	23	7	35	3	29
Peribán de Ramos	14	124.5	7	159	8	89.5	1	5	2	38
Tancítaro	16	263	23	163.5	32	387	34	240	6	274
Uruapan	80	909.5	54	806	125	571.5	110	771.5	49	430.5

Base de datos COFOM, 2010.

Características biológicas

Desde el punto de vista biológico, el Pico de Tancítaro es una de las áreas consideradas como prioritarias para la conservación (Conabio, 1999; Ordóñez y Flores Villela, 1995; Flores y Gerez, 1994); es la primera área con categoría de Área de Protección de Flora y Fauna para el estado de Michoacán y constituye la tercera Área Natural Protegida más grande del estado de Michoacán, después de las Reservas de la Biosfera Mariposa Monarca y Zicuirán-Infiernillo.

Vegetación

De acuerdo con la clasificación de Rzedowski (1978) y basado en la cobertura de vegetación de las cartas del INEGI, el área de estudio presenta los siguientes tipos principales de vegetación:

Cuadro 6. Tipos de cobertura vegetal en el APFF Pico de Tancítaro

Tipo de cobertura vegetal	Superficie (hectáreas)	Superficie (porcentaje)
Bosque de pino-encino	10,397.600627	44.43
Bosque de pino	3,764.657107	16.07
Bosque de pino abierto	2,624.124292	11.22
Pastizal	1,080.388521	4.62
Vegetación secundaria	504.981125	2.16
**Total de cobertura vegetal**	**18,371.751672**	**78.50**
**Otros**		
Área agrícola	5,013.529661	21.41
Asentamientos humanos	2.189298	0.01
Cuerpos de agua	18.450324	0.08
**Total otros**	**5,034.169283**	**21.50**
**Total**	**23,405.920955**	**100**

Bosque de pino-encino

Este tipo de vegetación se encuentra en laderas de los cerros Cuiritzarán, Zacán, Curupicho, Cutzato y Capatzin, en altitudes que van desde 2 mil 300 hasta 2 mil 750 metros sobre el nivel del mar. Comparte los mismos requerimientos ecológicos con el bosque de pino. Las especies más frecuentes son los pinos *(Pinus pseudostrobus, Pinus leiophylla, Pinus douglasiana* y *Pinus montezumae),* acompañados de encinos *(Quercus rugosa, Quercus martinezii, Quercus laurina, Quercus crassifolia, Quercus obtusata* y, en menor proporción, *Quercus candicans* y *Quercus crassipes).*

Otros elementos arbóreos comunes son el aile *(Alnus jorullensis* subsp. *lutea),* el aceituno *(Cleyera integrifolia),* la tila roja *(Ternstroemia lineata)* y el tejocote *(Crataegus pubescens).* Los arbustos que se presentan son de los géneros *Baccharis, Eupatorium, Senecio, Desmodium, Arctostaphylos, Coriaria, Cestrum, Lupinus, Ceanothus, Salvia, Satureja* y *Trisetum.* El estrato herbáceo es rico en especies de compuestas, gramíneas y leguminosas, así como de numerosos helechos. Este tipo de vegetación presenta asociaciones con vegetación secundaria.

Bosque de pino

Se distribuye en los cerros Capatzin, Choritiro, Cutzato, Juritzicuaro, Prieto y Pario, entre otros lugares, en donde por lo general tiene un manejo silvícola. El bosque es siempre verde y presenta troncos rectos de 20 a 30 metros o mayor altura cuando es más o menos cerrado. Las especies de pino *(Pinus*

pseudostrobus y *Pinus hartwegii)* son las más distribuidas; se encuentran desde los mil 900 hasta los 3 mil 100 metros sobre el nivel del mar y se asocian frecuentemente con pino *(Pinus leiophylla)* y en menor proporción con *Pinus montezumae* y *Pinus douglasiana*. Otros árboles asociados son el tejocote *(Crataegus pubescens)*, los encinos *(Quercus rugosa*, *Quercus laurina)*, el aceituno *(Cleyera integrifolia)*, la tila roja *(Ternstroemia lneata)*, el aile *(Alnus jorullensis* subsp. *Lutea)*, la mano de león *(Oreopanax xalapensis)* y el sauce *(Salix paradoxa)*. El sotobosque está compuesto por *Baccharis heterophylla*, senecio (*Senecio* spp.) y *Eupatorium* spp.

En las partes donde está perturbado y es abierto se consideró como vegetación secundaria, como en las laderas del Cerro Pario, estableciendo asociaciones densas de lupino *(Lupinus* spp.) y senecio (*Senecio* spp.) o nurite *(Satureja macrostema)*. En cambio, en el Cerro Capatzin se encuentra helecho de tierra *(Coriaria ruscifolia* subsp. *microphylla)* en el estrato arbustivo.

Bosque de pino abierto

Son formaciones con una distribución discontinua de árboles, pero con una cobertura de copa de al menos 10 por ciento y menos de 40 por ciento. Generalmente hay una cubierta continua de pasto, lo que permite el pastoreo y la propagación de incendios; este tipo de vegetación se encuentra en las partes más altas del área y se pueden observar especies como los pinos *(Pinus hartwegii, Pinus pseudostrobus, Pinus leiophylla, Pinus montezumae)*, el zacate *(Muhlenbergia macroura)* y el pericón *(Tagetes filifolia)*, así como enebro azul *(Juniperus monticola)* y cedro blanco *(Cupressus lusitanica)*, estas últimas sujetas a protección especial conforme a la Norma Oficial Mexicana Nom-059-Semarnat-2010, Protección ambiental-Especies nativas de México de flora y fauna silvestres-Categorías de riesgo y especificaciones para su inclusión, exclusión o cambio-Lista de especies en riesgo, que indica nueve especies enlistadas en la categoría de protección especial.

Pastizal

Predominan las gramíneas amacolladas en las partes más altas del Anp, como en los llanos intermontanos, como zacate *(Muhlenbergia macroura)* y pasto *(Festuca amplissima)*, alternando con pata de león *(Alchemilla procumbens)* y *Eupatorium pazcuarense*. En los llanos abundan zacate *(Muhlenbergia macroura)*, zacate barbón *(Aegopogon cenchroides)* y abrojo *(Acaena elongata)*.

Vegetación secundaria

Tanto la vegetación de pino como la de pino-encino tienen asociaciones de vegetación secundaria.

Se incluyen en esta categoría las comunidades de plantas que se establecen como consecuencia de disturbios, como las diversas arvenses, ruderales, matorral secundario, pastizal inducido y áreas reforestadas.

Existe la vegetación que se establece en áreas cubiertas por lava, como es el caso de la erupción del Volcán Paricutín. En este ambiente se observa un estrato

arbóreo muy disperso y de poca altura, con pinos *(Pinus montezumae, Pinus leiophylla),* tepozán *(Buddleia cordata)* y jaboncillo *(Clethra mexicana).* Entre los arbustos se encuentra *Baccharis heterophylla,* senecio *(Senecio salignus),* helecho de tierra *(Coriaria ruscifolia* subsp. *microphylla), Gaultheria cordata* subsp. *lancifolia* y madroño borracho *(Comarostaphylis discolor).* Las herbáceas más frecuentes son *Eupatorium pazcuarense, Eupatorium glabratum,* zacate barbón *(Aegopogon cenchroides)* y una gran cantidad de helechos de los géneros *Asplenium*, *Cheilanthes*, *Pleopeltis*, *Phlebodium*, *Polypodium*, *Pellaea* y *Elaphoglossum*, entre otros.

Según el listado de flora (Anexo I) realizado por (García Ruiz, 1999) y de acuerdo a la Norma Oficial Mexicana Nom-059-Semarnat-2010, Protección ambiental-Especies nativas de México de flora y fauna silvestres-Categorías de riesgo y especificaciones para su inclusión, exclusión o cambio-Lista de especies en riesgo, nueve especies están enlistadas en la categoría de protección especial (Pr) y una se encuentra como amenazada (A) (Cuadro 7).

Cuadro 7. Especies de flora registradas en la Norma Oficial Mexicana Nom-059-Semarnat-2010

Nombre científico	Nombre común	Categoría de riesgo
Cupressus lusitanica	cedro blanco	Pr
Juniperus monticola	enebro azul	Pr
Saurauia serrata	almasante, mameyito	Pr
Arbutus occidentalis	madroño	Pr
Phymosia rosea	vara de San Juan	Pr
Furcraea bedinghausii	palmita	A*
Gentiana spathacea	flor de hielo	Pr
Monotropa hypopitys	pipa de indio	Pr
Comarostaphylis discolor	madroño borracho	Pr
Dahlia scapigera	dalia	Pr

Pr: protección especial; A: amenazada; *(e): endémica.

Hongos

No se cuenta con información relevante sobre la variabilidad de especies de hongos en la región, pero por las mismas características de vegetación y humedad que presenta el área es de esperarse la presencia de éstos dentro del polígono. Se sabe que varios de los habitantes de las comunidades colindantes al polígono suben a la zona para obtener hongos para su uso o subsistencia.

Entre los trabajos realizados en la región está el de Fragoso (2003), quien registró algunas especies silvestres comestibles de hongos, entre los que destacan el hongo amarillo, pata de gallina y trompa de puerco, todos del género *Amanita*, los cuales crecen en

forma silvestre y están asociados a la temporada de lluvias, por lo que su abundancia es estacional.

Por su parte, Zamora (2006) realizó un inventario de hongos silvestres comestibles en una comunidad del municipio de Tancítaro y registró 16 especies de hongos silvestres comestibles, dos de ellos nuevos para el estado de Michoacán: *Amanita umbrinolutea* y amarillo *(Amanita calyptratoides)*.

Finalmente, en 2012 el Dr. Víctor Manuel García Reyes, de la Facultad de Biología de la UMSNH, realizó una revisión del inventario de hongos antes mencionado basándose en la lista de especies de macrohongos para el Tancítaro, encontrando 44 familias y 128 especies, de las cuales cinco se encuentran enlistadas en alguna categoría de riesgo de acuerdo a la Norma Oficial Mexicana Nom-059-Semarnat-2010, Protección ambiental-Especies nativas de México de flora y fauna silvestres-Categorías de riesgo y especificaciones para su inclusión, exclusión o cambio-Lista de especies en riesgo (Cuadro 8; Anexo I).

En algunos sitios, por el cambio de uso de suelo de forestal a agrícola, especialmente para cultivo de aguacate, se ha puesto en situación vulnerable la presencia y el uso de los hongos silvestres, entre ellos los comestibles, que en algunas comunidades son utilizados por un buen porcentaje de la población.

Cuadro 8. Especies de hongos con alguna categoría de riesgo de acuerdo a la Norma Oficial Mexicana Nom-059-Semarnat-2010

Nombre científico	Nombre común	Categoría de riesgo
Morchella angusticeps	colmenillas	A
Morchella esculenta	mazorca, mazorquita	A
Agaricus augustus	champiñón de bosque	A
Amanita muscaria	hongo tecomate de moscas	A
Hygrophorus russula	ririchaka (raramuri)	A

A: amenazada.

Fauna

El estado de Michoacán se encuentra en la zona de transición de las dos regiones que se localizan en México: la neártica y la neotropical. En el Eje Neovolcánico Transversal la fauna silvestre que se presenta es propia de climas templados, con algunas especies de lugares tropicales. Según Villaseñor y colaboradores (2005), la fauna del Pico de Tancítaro está conformada por un total de 370 de especies de vertebrados terrestres.

Mamíferos

En México se reportan 493 especies de mamíferos según Villa y Cervantes (2003), de las cuales 163 se indican para el estado de Michoacán (Conabio, 1998), mientras que para el Anp Pico de Tancítaro, antes de su recategorización, se habían registrado 86 especies de 18 familias, siendo las más numerosas Chiroptera y Rodentia, de acuerdo a la recopilación realizada por Villaseñor (2005) para la obra *La biodiversidad en Michoacán: estudio de estado,* basada en la literatura existente y la revisión de A. Núñez Garduño[1] (2005).

El listado de especies actualizado que se presenta aquí está basado en Villaseñor (2005) y Fuentes (2009), siguiendo el arreglo de Ceballos y Oliva (2005) con la revisión de Ma. Concepción Apátiga Castelán (*com. pers*.).[2] El total de especies registrado es de 95, que corresponden a 20 familias, siendo las más representativas el zorrillo manchado *(Conepatus leuconotus),* el conejo de monte *(Sylvilagus cunicularius),* la rata cambalachera de Tancítaro *(Nelsonia goldmani),* especie sujeta a protección especial, y la tuza michoacana *(Zygogeomys trichopus),* endémica del estado de Michoacán y en peligro de extinción de acuerdo a la norma referida; así como la ardilla *(Sciurus aureogaster),* el tlacuache *(Didelphis virginiana),* la liebre torda *(Lepus callotis),* el armadillo *(Dasypus novencinctus)* y el tejón *(Nasua narica),* entre otros (Anexo I).

21 especies de mamíferos registrados para el APFF Pico de Tancítaro son endémicas de México, o sea, el 22 por ciento de la riqueza total, lo que representa una oportunidad única para su conservación. La mitad de las especies endémicas son ratas y ratones de la familia Muridae; de tres especies de tuza, la conocida como tuza michoacana *(Zygogeomys trichopus)* es endémica y se encuentra en peligro de extinción de acuerdo con la Norma Oficial Mexicana Nom-059-Semarnat-2010, Protección ambiental-Especies nativas de México de flora y fauna silvestres-Categorías de riesgo y especificaciones para su inclusión, exclusión o cambio-Lista de especies en riesgo. También son endémicas dos especies de musarañas y dos de murciélagos (Cuadro 9).

[1] Dr. Arturo Núñez Garduño. Laboratorio de Mastozoología de la Facultad de Biología de la Universidad Michoacana de San Nicolás de Hidalgo.

[2] M. en C. Ma. Concepción Apátiga-Castelán. Laboratorio de Mastozoología de la Facultad de Biología de la Universidad Michoacana de San Nicolás de Hidalgo.

Cuadro 9. Especies de mamíferos endémicos de México registradas en el APFF Pico de Tancítaro

Familia	Nombre científico	Nombre común
Marmosidae	*Tlacuatzin canescens*	tlacuachín
Soricidae	*Cryptotis goldmani*	musaraña orejillas de Goldman
Soricidae	*Megasorex gigas*	musaraña
Phyllostomidae	*Artibeus hirsutus*	murciélago
Vespertilionidae	*Rhogeessa parvula*	murciélago
Sciuridae	*Spermophilus adocetus*	cuinique
Geomyidae	*Cratogeomys gymnurus*	tuza llanera
Geomyidae	*Cratogeomys tylorhinus*	tuza
Geomyidae	*Zygogeomys trichopus*	tuza michoacana
Muridae	*Nelsonia goldmani*	rata cambalachera de Tancítaro
Muridae	*Nelsonia neotomodon*	rata cambalachera diminuta
Muridae	*Osgoodomys banderanus*	ratón
Muridae	*Peromyscus difficilis*	ratón
Muridae	*Peromyscus hylocetes*	ratón
Muridae	*Peromyscus perfulvus*	ratón
Muridae	*Peromyscus spicilegus*	ratón
Muridae	*Reithrodontomys chrysopsis*	ratón
Muridae	*Reithrodontomys zacatecae*	ratón
Muridae	*Sigmodon alleni*	rata algodonera
Muridae	*Sigmodon mascotensis*	rata algodonera
Leporidae	*Sylvilagus cunicularius*	conejo de monte

Son 10 las especies que se encuentran en la Norma Oficial Mexicana Nom-059-Semarnat-2010, Protección ambiental-Especies nativas de México de flora y fauna silvestres-Categorías de riesgo y especificaciones para su inclusión, exclusión o cambio-Lista de especies en riesgo (Cuadro 10 Anexo I); cinco de ellas son endémicas de México. Entre las especies que enfrentan algún peligro de desaparecer o una disminución en sus poblaciones se encuentran cuatro musarañas, siendo la musaraña (*Megasorex gigas* [A*]) endémica de algunos estados del oeste mexicano y ubicada en la categoría de amenazada, debido a su rareza y a la destrucción de su hábitat (Ceballos y Miranda, 2000). Tres murciélagos de la familia Phyllostomidae también se encuentran amenazados, además de dos especies de ratas cambalacheras del género *Nelsonia*, endémicas de México que están en la categoría de protección especial (Cuadro 10).

Cuadro 10. Especies de mamíferos registrados en el APFF Pico de Tancítaro con alguna categoría de riesgo, enlistadas en la Norma Oficial Mexicana Nom-059-Semarnat-2010

Familia	Especie	Nombre común	Categoría de riesgo
Soricidae	*Cryptotis goldmani*	musaraña orejillas de Goldman	Pr*
Soricidae	*Cryptotis parva soricina*	musaraña orejillas mínima	Pr
Soricidae	*Megasorex gigas*	musaraña	A*
Soricidae	*Notiosorex crawfordi*	musaraña de Crawford	A
Phyllostomidae	*Choeronycteris mexicana*	murciélago trompudo	A
Phyllostomidae	*Leptonycteris curasoae*	murciélago hocicudo	A
Phyllostomidae	*Leptnonycteris nivalis*	murciélago hocicudo mayor	A
Geomyidae	*Zygogeomys trichopus*	tuza michoacana	P*
Muridae	*Nelsonia goldmani*	rata cambalachera de Tancítaro	Pr*
Muridae	*Nelsonia neotomodon*	rata cambalachera diminuta	Pr*

P: peligro de extinción; A: amenazada; Pr: protección especial; *(e): endémica; 1 endémica de Michoacán.

Aves

Michoacán es uno de los estados con mayor diversidad en cuanto a aves se refiere, entre las 10 entidades con mayor riqueza en el país; según L. E. Villaseñor Gómez (2011, *com. pers.*), se cuenta con un registro de 556 especies de aves.

En la Región del Pico de Tancítaro y en el Anp se ubica un área de importancia para la conservación de las aves (AICA C-5) (Villalón *et al.*, 2000, en Arizmendi y Márquez Valdelamar, 2000). En la Figura 2 se muestra la superposición de las tres áreas de conservación y los límites del municipio de Tancítaro.

Por otra parte, los estudios sobre la avifauna de la región se iniciaron con la investigación de E. W. Nelson y E. A. Goldman, quienes en 1903 registraron 89 especies de aves (Goldman, 1951). Para las regiones del Pico de Tancítaro y la cuenca del Río Balsas-Tepalcatepec, Blake y Hanson (1942) reportaron 140 especies a lo largo de un transecto altitudinal en los veranos de 1940 y 1941; enlistaron 47 especies características de las zonas templadas y húmedas tropical superior, arriba de los mil 800 metros sobre el nivel del mar. Mientras que para la Sierra Purépecha, Salas (1986) reportó 150 especies entre 1982 y 1985.

Como parte de las actividades realizadas por personal del Laboratorio de Ornitología de la Universidad Michoacana, a partir de la década de los setenta en la región de Tancítaro, se cuenta con un inventario derivado de diversas visitas de campo (*e. g.* Méndez *et al.*, 1987), principalmente entre 1990 y 1991, reportándose 190 especies de aves (Villaseñor y Villaseñor, 1992) para el Área. Otro estudio desarrollado en la región es el de Villalón (1990), quien hizo un análisis del transecto Tancítaro-Parácuaro, obteniendo un listado de 269 especies de aves. En la obra *La*

biodiversidad de Michoacán: estudio de estado, Villaseñor (2005) enlistó 195 especies para el Área Natural Protegida antes de su recategorización.

En la región del APFF Pico de Tancítaro coinciden la Región Terrestre Prioritaria RTP114-Tancítaro (Arriaga *et al.,* 2002; Conanp, 2008) y el área de importancia para la conservación de las aves (AICA C-5), la cual cubre una superficie de 216 mil 790 hectáreas y en donde se registraron 256 especies de aves. Es importante como zona de estancia y alimentación, así como sitio de migraciones altitudinales y es sitio de una alta diversidad de especies endémicas, especialmente en los bosques de pino-encino y encino (Villalón *et al.,* 2000, en Arizmendi y Márquez Valdelamar, 2000). En una revisión actualizada de la riqueza del AICA Pico de Tancítaro en 2006 por L. E. Villaseñor se reportaron 305 especies de aves; el listado, producto de dicha revisión, se encuentra en la página de la Conabio,[2] tanto para la región del Parque Nacional Pico de Tancítaro, como para el AICA 5-Tancítaro, aun cuando los polígonos de ambas áreas son de diferente superficie.[3]

Se presenta un listado de 241 especies de aves que corresponden a 48 familias de 17 órdenes (Anexo I) para el APFF Pico de Tancítaro, siguiendo el arreglo taxonómico y la nomenclatura de la *American Ornithologist's Union* (AOU, 1998, más suplementos 42-52: AOU, 2000; Banks *et al.,* 2002, 2003, 2004, 2005, 2006, 2007 y 2008; Chesser *et al.,* 2009, 2010 y 2011) y los nombres comunes de Escalante *et al.*, (1996). Para la elaboración del listado se revisó la base de datos y la literatura citada, además de Hernández Zamudio (2011), y las especies registradas en la página de la Conabio mencionada en el párrafo anterior; se eliminaron las especies que no corresponden a las regiones templadas y frías y a las de ambientes acuáticos, salvo las registradas puntualmente dentro del Anp.

22 especies se encuentran en la Norma Oficial Mexicana Nom-059-Semarnat-2010, Protección ambiental-Especies nativas de México de flora y fauna silvestres-Categorías de riesgo y especificaciones para su inclusión, exclusión o cambio-Lista de especies en riesgo, 17 se encuentran en la categoría de protección especial (Pr), cuatro están amenazadas (A) y una en peligro de extinción (P) (Cuadro 10). También se señalan las tres categorías de endemismo que se reconocen para la avifauna mexicana en el listado del Anexo I: (E)= endémica exclusiva de México; CE= cuasiendémica; SE= semiendémica (González García y Gómez de Silva 2003), las cuales suman un total de 54 especies (Villaseñor Gómez, L. E., *com. pers.*).

[2] http://avesmx.conabio.gob.mx/lista_region

[3] M. en C. Laura E. Villaseñor Gómez, curadora de la Colección de Aves de la Facultad de Biología.

Figura 2. Polígonos de las tres áreas de conservación

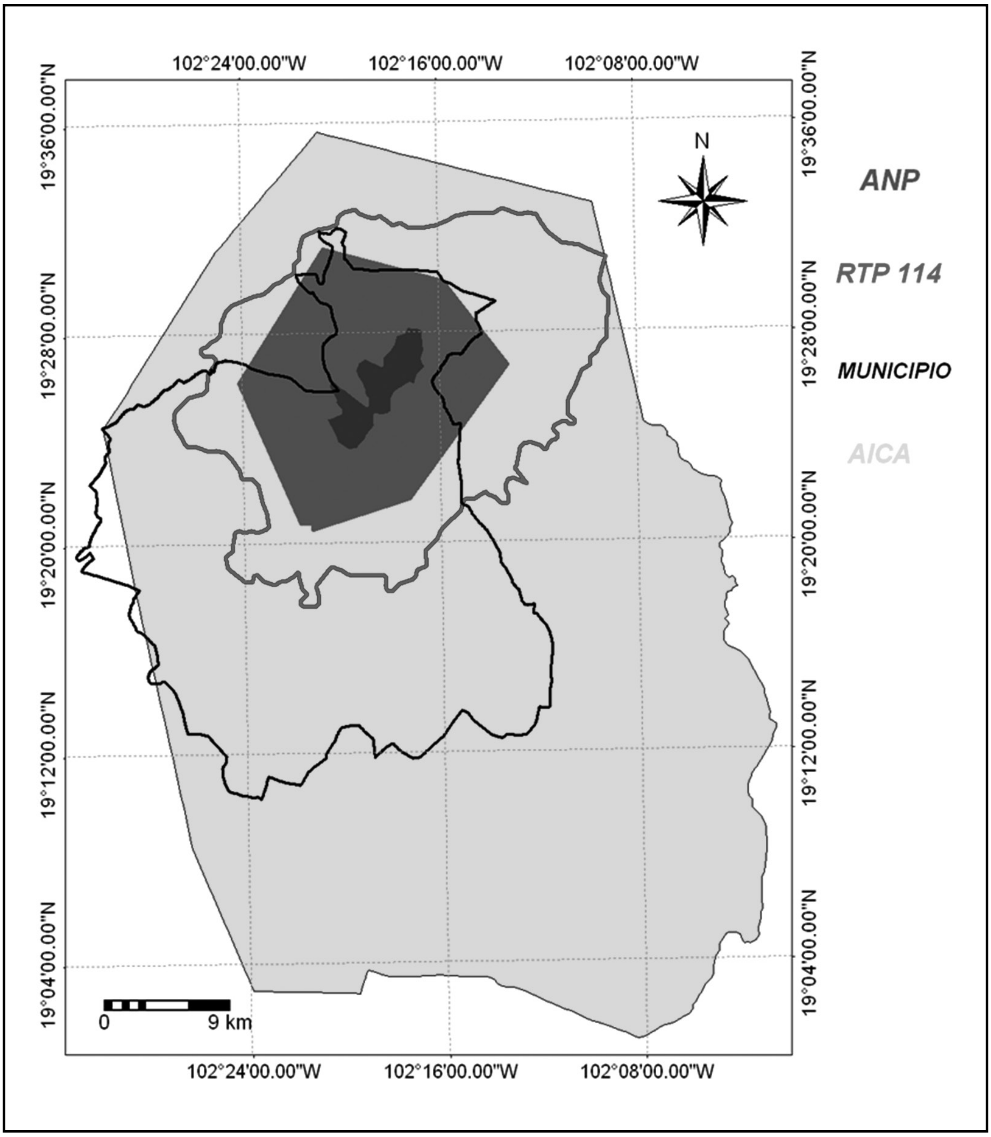

ANP= Área de Protección de Flora y Fauna Pico de Tancítaro; AICA= área de importancia para la conservación de las aves 05-Tancítaro; RTP= Región Terrestre Prioritaria 118-Tancítaro y el municipio de Tancítaro (tomado de Hernández Zamudio, 2011).

Cuadro 11. Especies de aves en alguna categoría de riesgo de acuerdo a la Norma Oficial Mexicana Nom-059-Semarnat-2010

Nombre científico	Nombre común	Categoría de riesgo
Rhynchopsitta pachyrhyncha	cotorra-serrana occidental	P*
Dendrortyx macroura	codorniz-coluda neovolcánica	A*
Penelope purpurascens	pava cojolita	A
Euptilotis neoxenus	trogón orejón	A*
Catharus frantzii	zorzal de Frantzius	A
Streptoprocne semicollaris	vencejo nuca blanca	Pr*
Vireo nelsoni	víreo enano	Pr*
Ridgwayia pinicola	mirlo pinto	Pr*
Cyrtonyx montezumae	codorniz Moctezuma	Pr
Accipiter striatus	gavilán pecho rufo	Pr
Accipiter cooperi	gavilán de Cooper	Pr
Parabuteo unicinctus	aguililla rojinegra	Pr
Buteo lineatus	aguililla pecho rojo	Pr
Buteo platypterus	aguililla ala ancha	Pr
Buteo swainsoni	aguililla de Swainson	Pr
Buteo albicaudatus	aguililla cola blanca	Pr
Buteo albonotatus	aguililla aura	Pr
Strix varia	búho listado	Pr
Cinclus mexicanus	mirlo-acuático norteamericano	Pr
Myadestes occidentalis	clarín jilguero	Pr
Vermivora crissalis	chipe crisal	Pr
Passerina ciris	colorín sietecolores	Pr

Pr: peligro de extinción; P: protección especial; A: amenazada. *(e): endémica.

Anfibios y reptiles

Con base en los estudios realizados sobre la herpetofauna del municipio de Tancítaro se tiene un registro potencial de 56 especies de anfibios y reptiles. Las 14 especies de anfibios pertenecen a seis familias y 11 géneros, siendo *Incilius* el género más representativo. Las 42 especies de reptiles están agrupadas en 11 familias, siendo los géneros más representativos *Sceloporus* y *Crotalus* (Estrada, 2001; Alvarado y Campbell, 2004; Villaseñor, 2005; Fuentes, 2009). Recientemente fue descrita una nueva especie de víbora de cascabel por Alvarado y Campbell (2004) en el Pico de Tancítaro *(Crotalus tancitarensis)*.

Son 24 especies de anfibios y reptiles las que se encuentran enlistadas en la Norma Oficial Mexicana Nom-059-Semarnat-2010, Protección ambiental-Especies nativas de México de flora y fauna silvestres-Categorías de riesgo y especificaciones para su inclusión, exclusión o cambio-Lista de especies en riesgo, de las cuales nueve se encuentran

en la categoría de amenazadas (A) y 15 en la categoría de sujetas a protección especial (Pr) (Cuadro 12). En los dos grupos se encuentra un alto porcentaje de endemismos (71.4 por ciento), 10 de las 14 especies de anfibios y 30 de las 41 especies de reptiles se encuentran exclusivamente en México (Anexo I).

Cuadro 12. Anfibios y reptiles en alguna categoría de riesgo de acuerdo a la Norma Oficial Mexicana Nom-059-Semarnat-2010

Nombre científico	Nombre común	Categoría de riesgo
Pseudoeurycea belli	tlaconete pinto	A*
Eleutherodacylus angustidigitorum	rana de Pátzcuaro	Pr*
Plectrohyla bistincta	rana de pliegue	Pr*
Lithobates forreri	rana	Pr
Lithobates montezumae	rana de Moctezuma	Pr*
Kinosternon integrum	tortuga pecho quebrado mexicana	Pr*
Ctenosaura pectinata	iguana espinosa mexicana	A*
Phrynosoma asio	llora sangre	Pr
Sceloporus grammicus	lagartija escamosa de mezquite	Pr
Sceloporus scalaris	lagartija de montaña	Pr
Plestiodon copei	eslizón de Cope	Pr*
Plestiodon dugesi	eslizón de Duges	Pr*
Boa constrictor	boa, alamacoa	A
Barisia imbricata	falso escorpión	Pr*
Conopsis biserialis	culebra mexicana de dos líneas	A*
Geophis tarascae	culebra minadora tarasca	Pr*
Leptoderia maculata	culebra ojo de gato del suroeste	Pr*
Lampropeltis triangulum	falsa coralilla	A
Pituophis deppei	mazacuate	A*
Salvadora bairdi	culebra chata de Baird	Pr*
Thamnophis cyrtopsis	culebra de agua nómada de cola larga	A
Thamnophis scalaris	culebra lineada de bosque	A*
Crotalus basiliscus	cascabel	Pr*
Crotalus pusillus	cascabel oscura del Tancítaro	A*

Pr: peligro de extinción; P: protección especial; A: amenazada. *(e): endémica.

Servicios ambientales

El Área Natural Protegida tiene un gran valor ambiental, en cuanto a los beneficios directos que brinda: los servicios hidrológicos (ver Hidrología), ya que cuenta con zonas de captación y escurrimientos permanentes o temporales de agua; la existencia del Área Natural Protegida apoya en la regulación de ciclos como la prevención de inundaciones, principalmente para las poblaciones existentes en la Zona de Influencia del área; ayuda a controlar la degradación de los suelos y a la regulación del clima regional; controla la desecación; y provee de agua a las comunidades dedicadas al cultivo de aguacate, manzana, pera, agricultura de temporal y ganadería extensiva. Los beneficios indirectos que el Área Natural Protegida brinda son los que se relacionan con el funcionamiento de procesos del ecosistema que generan los servicios directos antes mencionados y que ayudan al proceso de fotosíntesis, a la captura de carbono y la formación y almacenamiento de materia orgánica, el ciclo de nutrientes, la creación y asimilación del suelo, y la conservación de la gran diversidad que alberga, además de los valores estéticos y los servicios de recreación, como días de campo, excursiones y observación de paisajes para las y los habitantes de la Zona de Influencia del Área Natural Protegida principalmente.

Contextos histórico, cultural y arqueológico

Historia del área

La historia del área se remonta hasta épocas prehispánicas, entre los años 1400 y 1440 d.C., cuando en su expansión los herederos de Tariácuri, Hiripan y Tangansoan I, ocuparon el territorio que comprende los valles en las faldas del Cerro de Tancítaro, habitado entonces por los aguerridos "Tecos", de origen náhuatl.

El poblado de Tancítaro se fundó como sitio para entrega de los tributos recaudados de los centros a su cargo. De ahí que se le dio el nombre de "Tantzita" en lengua purhembe, que significa "lugar de o en que se entrega el tributo", y derivó posteriormente en el nombre de Tancítaro.

De acuerdo con Bocco y Garibay (2000), desde el siglo XVI prefiguran los rasgos generales de la apropiación efectiva del territorio del área actual. Se forman las "congregaciones de indios" de San Juan Parangaricutiro, de San Salvador Cumbutzio, de Santa Ana Zirosto, de Peribán de Ramos; de Apo y de Tancítaro; y se asiste al nacimiento de ranchos y haciendas, propiedad de criollos y españoles.

En 1620 el poblado quedó habitado solo por nativos y se inició la compraventa de terrenos para solicitar una composición de las tierras que les restaban y pagar la indemnización correspondiente. Fue hasta 1882 cuando se entregó a los comuneros el título que ampara su propiedad.

Entre 1933 y 1934 se formaron los primeros ejidos en Tancítaro, con afectaciones sobre los terratenientes. En 1960, al construirse la brecha Tancítaro-San Juan Nuevo, surgieron las primeras huertas de aguacate a iniciativa de seis comuneros, cultivo que con el tiempo alcanzaría mayor auge.

Durante las décadas previas al Decreto de 1940 del antes “Parque Nacional Pico de Tancítaro”, se presentó un escenario donde la tierra estaba segmentada en múltiples propiedades de diversos tamaños, algunas en manos de indígenas, otras de prestigiadas familias mestizas dedicadas al comercio regional, otras más de propietarios foráneos que mediante compraventa o que por simple declaratoria de propiedad adquirieron amplias superficies en la perspectiva empresarial de aprovechar los ricos bosques de la región para elaborar durmientes de ferrocarril.

En 1940 el presidente Lázaro Cárdenas publicó en el *Diario Oficial de la Federación*, un Decreto que declara como Parque Nacional la región conocida como Pico de Tancítaro. Décadas después, dicha área fue cambiada a una categoría acorde a su dinámica actual, como Área de Protección de Flora y Fauna, la cual fue publicada y declarada en el DOF, el 19 de agosto de 2009, por el presidente Felipe de Jesús Calderón Hinojosa.

Cultura

Las actividades culturales descritas a continuación forman parte de los cuatro municipios que conforman el Área Natural Protegida y su Zona de Influencia; sin embargo, con estas actividades podemos darnos cuenta de que la mayoría de los pobladores aún tienen arraigadas gran parte de sus tradiciones y costumbres.

La riqueza cultural del pueblo purépecha es un mosaico que involucra mil y una variantes expresadas en la fiesta del color, las artesanías, las formas de convivencia, la medicina y por supuesto las formas de alimentación, entre muchos aspectos que definen la razón de los purépechas.

La alimentación es parte fundamental de la identidad cultural de la región purépecha, conservándose diversas formas de aprovechamiento de los recursos naturales con que se cuentan, incluidas la plantas cultivadas y las de origen natural que se tienen en la milpa, los terrenos de descanso, el solar o el bosque y los diferentes tipos de comida de uso cotidiano, ceremonial y religioso para agradecer la bondad de la cosecha, para celebrar el día del patrono o para festejos especiales de la familia, los barrios y la comunidad en general.

Los cuatro municipios que abarcan el Área Natural Protegida cuentan con sus propias tradiciones; en la cabecera municipal de Tancítaro la música tradicional es la música de viento, como parte de su gastronomía se pueden mencionar las carnitas, las corundas, los huchepos y el guacamole, sus fiestas tradicionales son las Patronales (Señor del Perdón) y en el mes de junio es la fiesta del pueblo (Corpus Cristi).

En San Juan Nuevo Parangaricutiro se cuentan las Fiestas de Reyes con las famosas danzas de los curpites; además la celebración de las fiestas del Señor de los Milagros, en honor del Cristo que permaneció intacto al destruirse su capilla por el temblor de 1943.

En el municipio de Uruapan se inauguran las tradicionales fiestas de Domingo de Ramos, en las que los artesanos participantes desfilan por orden alfabético, según su comunidad de

origen; el desfile se acompaña con bandas musicales de las distintas comunidades y las danzas con las y los participantes. Otra ceremonia que se celebra, es la de las Aguadoras, que se realiza el Sábado de Gloria.

Desde hace 16 años en la ciudad de Uruapan, como centro del universo purépecha, se han venido presentando los platillos más representativos de la cocina regional y buena parte de la población de la ciudad, de comunidades vecinas y de otros pueblos y estados han disfrutado de la presentación gastronómica el Domingo de Ramos.

En Peribán de Ramos se celebran las fiestas tradicionales de la región Purépecha, debido a la cercanía con la zona indígena, la música tradicional es la banda de viento de la Meseta Purépecha. En cuanto a las artesanías, se realizan principalmente las peribanas, que son bateas de madera laqueadas en finos acabados en oro, características por su gran tamaño. La comida típica regional consiste en algunos platillos preparados con el principal producto de la región, el aguacate: guacamole, salsa de aguacate y nieve de aguacate, entre otros (Enciclopedia de los Municipios de México, Michoacán, 2009).

Contextos demográfico y económico

Contexto demográfico

Como se ha mencionado anteriormente, la región del Área Natural Protegida comprende los municipios de Tancítaro, Peribán de Ramos, Nuevo Parangaricutiro y Uruapan, todos ellos en el estado de Michoacán.

En estos municipios existen 837 localidades, de las cuales 439 pertenecen al municipio de Uruapan, 176 al municipio de Tancítaro, 137 al municipio de Peribán de Ramos y 85 al municipio de Nuevo Parangaricutiro; sin embargo, según los datos del INEGI 2009, dentro del Área Natural Protegida solo existen 21 localidades con un total de mil 253 habitantes;[4] la mayor parte de estos habitantes pertenecen al municipio de Tancítaro con 959 habitantes.

Una localidad pertenece al municipio de Uruapan, cuatro localidades al municipio de Nuevo Parangaricutiro, siete localidades al municipio de Tancítaro y nueve localidades al municipio de Peribán de Ramos.

La cantidad de habitantes dentro del Anp es muy baja (mil 253 habitantes);[5] es importante aclarar que el INEGI no cuenta con datos básicos para las siguientes localidades: El Puerto, perteneciente al municipio de Peribán de Ramos, y La Arena, El Tepetate y Tizcato, pertenecientes al municipio de Nuevo Parangaricutiro (Cuadro 13).

[4, 5] Este dato representa únicamente el total de la población de 16 localidades; para el resto de las localidades se carece de información.

Cuadro 13. Población total y localidades de los municipios incluidos dentro del Área Natural Protegida

Municipio	Localidades por municipio	Población total de las localidades por municipio
Nuevo Parangaricutiro	La Escondida, Tizcato, El Tepetate, La Arena	42*
Peribán de Ramos	Cinco Pinos, Uña de Gato, El Ojo de Agua de Parastaco, Tangancicato, Paso de la Nieve, Los Desmontes, La Majada, El Puerto, El Granado	237**
Tancítaro	Zirimondiro, La Joya, Los Desmontes, La Chivera, Charapóndiro, El Jazmín, Huaríndaro	959
Uruapan	La Majada	15
**TOTAL**		**1,253[1]**

Fuente: INEGI, 2010; *Información de una localidad; **Información de ocho localidades; 1: este dato representa únicamente el total de la población de 16 localidades, el resto de las localidades carece de información.

En cuanto a la población por sexo, no existe gran diferencia en el número de hombres y mujeres que habitan las localidades dentro del Área Natural Protegida, aunque el porcentaje indica que existe una mayor presencia de hombres, que representan alrededor de 51 por ciento del total poblacional (Cuadro 14).

Cuadro 14. Población total de cada uno de los municipios y cantidad de hombres-mujeres dentro de los mismos

Municipio	Hombres	Mujeres	Población total por localidad
Nuevo Parangaricutiro	23	19	42
Peribán de Ramos	130	99	237*
Tancítaro	475	452	959*
Uruapan	6	9	15
**Total**	**634**	**579**	**1,253**

INEGI, Censo de población 2010.
* Nota: En el caso de algunas comunidades de los municipios de Peribán de Ramos y Tancítaro el INEGI no reporta la población desglosada por sexo. Por esta razón, la suma de las columnas "Hombres" y "Mujeres" puede no resultar en la cifra señalada en la columna "Población total por localidad".

Con relación al grado de escolaridad promedio, la localidad de Cinco Pinos, en el municipio de Peribán de Ramos, es la que cuenta con el nivel más alto, con 4.33, mientras que la localidad de El Ojo de Agua de Parastaco cuenta con el nivel más bajo, con 1.83; esto tomando en cuenta que para el estado de Michoacán

el grado de escolaridad promedio es de 6.9; el nivel de escolaridad promedio de estas localidades es muy bajo, aunado a que Michoacán se encuentra en cuarto lugar a nivel nacional, con el menor grado de escolaridad.

En cuanto al nivel de analfabetismo en el Área Natural Protegida, del total de la población que vive dentro del polígono y tomando en cuenta la población de 15 años y más, el 13 por ciento de las personas son analfabetas, esto considerando que no se cuenta con datos de todas las localidades.

En los cuatro municipios que abarcan el Área Natural Protegida la principal lengua indígena es la purépecha (INEGI, 2000).

Contexto socioeconómico

Desde el punto de vista forestal y económico y según el Anuario Estadístico de Michoacán de INEGI (2008), la región forma parte de los 52 municipios con una producción forestal maderable significativa y los municipios en los que se encuentra la montaña prioritaria del Tancítaro, participan con 13.7 por ciento de la producción total de Michoacán.

El Pico de Tancítaro como Sistema Hidrológico, con sus 16 cuencas, constituye la base del desarrollo de al menos 39 mil 783 habitantes que residen en las localidades dentro y fuera del Área Natural Protegida y que dependen del agua de este Sistema Hidrológico; hay 81 poblaciones y comunidades que se dedican al cultivo de durazno, manzana, ciruela, pera, caña de azúcar y principalmente aguacate, por lo que el área y sus alrededores constituyen una de las áreas más importantes del país en la producción de aguacate de exportación, incluyéndose en la zona conocida como "el corredor aguacatero" o franja aguacatera de Michoacán (Aguilera Montañez y Salazar García, 1991; Torres y Bocco, 1999; Gutiérrez Contreras *et al.*, 2007, Echánove 2008), cultivos cuya característica intrínseca es el requerimiento de grandes cantidades de agua.

En general, los procesos de producción agrícola (fruticultura) son los predominantes en los municipios que forman parte del Área Natural Protegida y su Zona de Influencia, además del "oro verde", como suele llamársele al aguacate, otros cultivos de ciclo largo o perenne y con riego son principalmente el durazno, el nanche y la caña de azúcar, entre otros.

De las 21 localidades que forman parte del Área Natural Protegida, el 23 por ciento de la población es económicamente activa; de esta población el 18 por ciento se dedica al sector primario, en el que las actividades principales son la agricultura, la ganadería y la silvicultura; el tres por ciento se dedica al sector secundario, en el que las principales actividades son la construcción y la industria manufacturera principalmente; el dos por ciento se dedica al sector terciario, en el que las principales actividades son el comercio, los servicios, las comunicaciones y el transporte. Cabe señalar que no se cuenta con datos de las localidades de San Juan Nuevo Parangaricutiro ni de algunas localidades de otros municipios, según los datos económicos del INEGI (2000).

Finalmente, es necesario destacar que en el municipio de Nuevo Parangaricutiro la principal actividad productiva es la forestal, que se centra en el aprovechamiento de madera y resina en los bosques de pino, encino y en menor medida de oyamel, actividad principal desde 1982 y considerada de gran importancia para la región (OET, 2009).

Contexto social

En cuanto a los servicios médicos a los que tienen acceso los habitantes de los municipios dentro del polígono del Área Natural Protegida, específicamente seguro social, en los municipios de Uruapan y Nuevo Parangaricutiro ningún habitante cuenta con seguro social y los habitantes de los municipios de Tancítaro y Peribán de Ramos, con 87 y 72 por ciento, respectivamente, no cuentan con seguro social (INEGI, 2005).

Servicios de vivienda

La siguiente información corresponde a los servicios de vivienda a los que tienen acceso los habitantes de los municipios y las localidades que viven dentro del Anp.

En total se encuentran 230 viviendas habitadas, de las cuales en 9.6 por ciento se cocina con gas y el 83 por ciento con leña, del siete por ciento restante se desconocen los datos; el 83 por ciento de las viviendas cuentan con agua entubada, pero del 17 por ciento restante se desconocen datos; el 10 por ciento de las viviendas cuentan con drenaje y el 60 por ciento de las viviendas cuentan con energía eléctrica, del resto no se cuenta con datos.

Vocación natural del suelo

Muchos de los terrenos que en 1974 tenían una cobertura predominantemente arbustiva como resultado de la erupción del Volcán Paricutín, hoy en día se manifiestan como bosques cerrados con coberturas que oscilan entre 70 y 80 por ciento de dosel. Estas superficies se localizan al norte del Tancítaro, muy cerca de la mesa Isingo y de las lavas del Paricutín.

Otro sector que presentó cambios después de la erupción del volcán de bosque abierto a bosque cerrado se localiza en la cara noroeste del Tancítaro, ocupando parte de las laderas externas, los valles angostos y el piedemonte superior. Este sector forma una franja que pasa entre las comunidades de La Majada y Parastaco.

Lo anterior conduciría a pensar erróneamente que los bosques están en recuperación. Sin embargo, existen objeciones al respecto:

Existe un impresionante aumento de la cobertura de cultivos permanentes en los últimos 20 años, en detrimento de la superficie de cultivos anuales y pastizales principalmente. Los cultivos permanentes pasaron en 1974 de solo el 3.2 por ciento con dos mil 196 hectáreas, a 31.1 por ciento con 21 mil 186 hectáreas. Es decir, la superficie de frutales creció 10 veces en 20 años. Entre tanto, los cultivos anuales decrecieron hasta 7.1 por ciento y los pastizales hasta 1.1 por ciento, después de representar el 23.3 por ciento y el 4.2 por ciento, respectivamente, en 1974. En otras palabras, la presión que se ejerce

actualmente sobre los bosques es mayor que antes, ya que se debe agregar a los cultivos permanentes como un elemento espacial de mayor movilidad (Fuentes y Alvarado, 2006).

La dinámica fuera del área es esencialmente distinta, debido a que dentro de los límites del Tancítaro los bosques tienen una menor presión, mientras que en los terrenos ubicados fuera del Área Natural Protegida los bosques han ido perdiendo terreno. Es de esperar que la tendencia a un comportamiento centrípeto del cambio de uso del suelo se mantenga y recrudezca ante la falta de terrenos agrícolas y el aumento poblacional.

Sin embargo, esta dinámica de cambio estará limitada por la altitud, la pendiente cada vez más pronunciada hacia las cimas del Tancítaro y la inaccesibilidad a estos terrenos. Todos estos factores desempeñan un papel importante a favor de la conservación, mientras que la figura legal del Área de Protección de Flora y Fauna tendrá una influencia muy secundaria (Fuentes y Alvarado, 2006).

La situación actual que presenta el Anp pone de manifiesto un incremento en el cambio de uso de suelo 10 veces mayor en los últimos 20 años, registrando una pérdida de masa forestal de 52.7 por ciento de bosque cerrado solamente; además, se ha presentado un incremento de la población en los últimos 10 años, erosión y disminución de agua dentro de la zona.

Como puede observarse, esta región ha experimentado un alto grado de transformación y como consecuencia un proceso de empobrecimiento tanto biológico como cultural. Los grandes cambios del uso de suelo han sido inducidos por el hombre (Vitousek *et al.*, 1997; Noble y Dirzo, 1997; Cincotta *et al.*, 2000) y una mínima proporción es producto de acciones naturales como huracanes, incendios y vulcanismo, entre otros.

La dinámica de cambio de zonas agrícolas de cultivos perennes se considera el principal desencadenador del cambio de uso del suelo, como se describe en las cifras presentadas a continuación:

Michoacán se destaca por su producción frutícola en México, siendo el aguacate Hass el principal cultivo tanto por su extensión (96 mil 764 hectáreas) como por su producción (un millón 3 mil 450 toneladas anuales; SIAP-SAGARPA, 2006), lo que representa el 29 por ciento del total de la superficie sembrada y el 36.5 por ciento de la producción mundial, ubicándolo como el principal productor en el orbe; tiene además el mayor consumo per cápita anual del mundo, con cerca de 10 kilogramos por habitante, además de ser el principal exportador, con el 22 por ciento del total mundial (Anguiano *et al.*, 2006).

Es importante mencionar que entre 1980 y 2008 la superficie cultivada con aguacate tuvo un incremento de 32 por ciento (SIAP-SAGARPA, 2006), mientras que la superficie de bosque templado disminuyó 74 por ciento entre 1970 y 2008 (Bocco y Mendoza, 1999), y el 20 por ciento de las áreas ocupadas actualmente por huertas de aguacate se encontraban cubiertas por bosque en 1996 (Chávez León *et al.*, 2008).

Se puede decir que las superficies se han modificado intensamente, afectando sobre todo los cultivos anuales y permanentes, que presentan dinámicas de cambio opuestas e intensas. Las tendencias generales del cambio de uso de suelo han sido constantes hacia la deforestación.

Análisis de la situación de la tenencia de la tierra

El establecimiento de las Áreas Naturales Protegidas en México tiene su base en la Constitución Política de los Estados Unidos Mexicanos, en la Ley General del Equilibrio Ecológico y la Protección al Ambiente y en su Reglamento en Materia de Áreas Naturales Protegidas, y constituye el asentamiento de modalidades a la propiedad pública, social y privada, se fundamenta en el interés público que representa la conservación y otros usos de la diversidad biológica y del medio ambiente.

Los diversos tipos de tenencia de la tierra dentro de un Área Natural Protegida permiten reconocer los usos de suelo y la vocación de los predios, razón por la que resulta indispensable conocer el porcentaje dentro de la poligonal del área respectiva de la propiedad pública, social y privada, puesto que al regular actividades permitidas y no permitidas en relación con las subzonas del presente Programa de Manejo, resulta importante tener una idea clara de las modalidades de uso de los recursos naturales en relación al régimen de propiedad de que se trate.

En el caso del Área de Protección de Flora y Fauna Pico de Tancítaro, existen dos tipos de tenencia de la tierra: propiedad social y propiedad privada, de conformidad con la información extraída de la página electrónica del Registro Agrario Nacional (http://sig.ran.gob.mx): la propiedad social en el Área Natural Protegida abarca aproximadamente el 50 por ciento de la superficie, localizada principalmente en la parte norte, oriental y suroriental del Área Natural Protegida y hacia el oeste de la montaña Pico de Tancítaro.

Por lo que respecta a la propiedad privada, el Registro Agrario Nacional reporta que es del 20 por ciento de la superficie total del Anp y se encuentra principalmente hacia el sur, el oeste y el noroeste de la misma.

Cabe señalar que del 30 por ciento restante no se encontró información disponible en la página electrónica del Registro Agrario Nacional.

Figura 3. Mapa de la situación de la tenencia de la tierra en el Área de Protección de Flora y Fauna Pico de Tancítaro

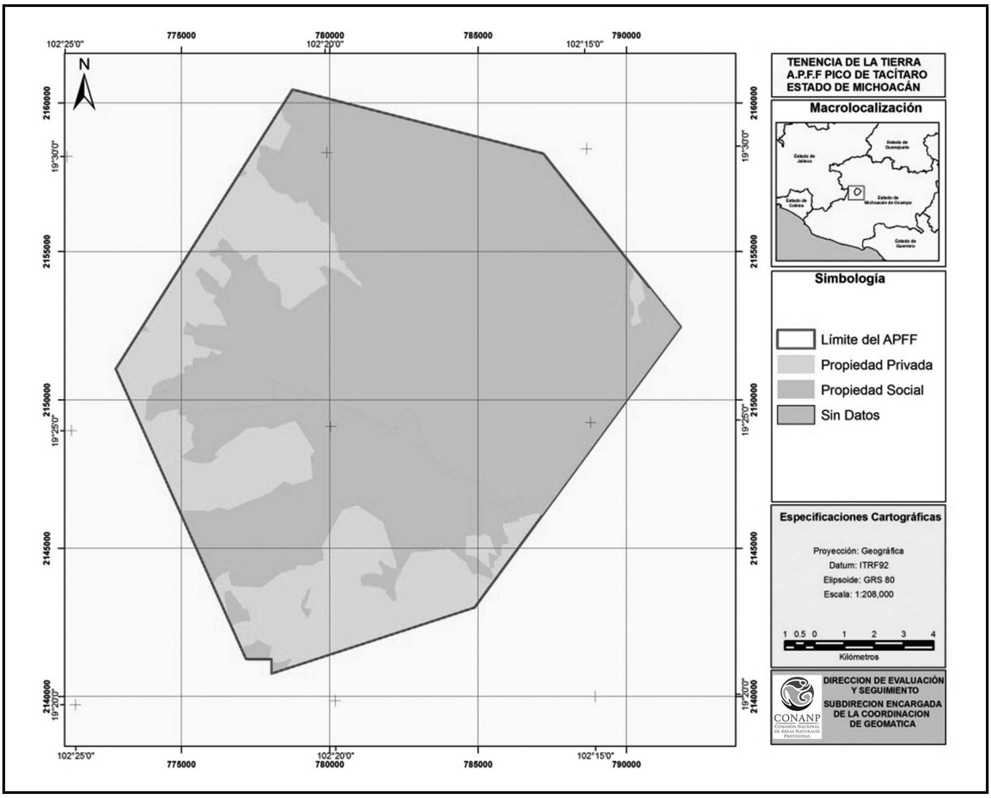

Normas Oficiales Mexicanas aplicables a las actividades a que esté sujeta el Área Natural Protegida

A continuación se enlistan las Normas Oficiales Mexicanas que en materia de recursos naturales son aplicables al Área Natural Protegida:

- Norma Oficial Mexicana Nom-005-Semarnat-1997, Que establece los procedimientos, criterios y especificaciones para realizar el aprovechamiento, transporte y almacenamiento de corteza, tallos y plantas completas de vegetación forestal.
- Norma Oficial Mexicana Nom-007-Semarnat-1997, Que establece los procedimientos, criterios y especificaciones para realizar el aprovechamiento, transporte y almacenamiento de ramas, hojas o pencas, flores, frutos y semillas.
- Norma Oficial Mexicana Nom-011-Semarnat-1996, Que establece los procedimientos, criterios y especificaciones para realizar el aprovechamiento, transporte y

almacenamiento de musgo, heno y doradilla.

- Norma Oficial Mexicana Nom-012-Semarnat-1996, Que establece los procedimientos, criterios y especificaciones para realizar el aprovechamiento, transporte y almacenamiento de la leña para uso doméstico.

- Norma Oficial Mexicana Nom-015-Semarnat/SAGARPA-2007, Que establece las especificaciones técnicas de métodos de uso del fuego en los terrenos forestales y en los terrenos de uso agropecuario.

- Norma Oficial Mexicana Nom-019-Semarnat-2006, Que establece los lineamientos técnicos para el combate y control de los insectos descortezadores.

- Norma Oficial Mexicana Nom-026-Semarnat-2005, Que establece los criterios y especificaciones técnicas para realizar el aprovechamiento comercial de resina de pino.

- Norma Oficial Mexicana Nom-027-Semarnat-1996, Que establece los procedimientos, criterios y especificaciones para realizar el aprovechamiento, transporte y almacenamiento de tierra de monte.

- Norma Oficial Mexicana Nom-059-Semarnat-2010, Protección ambiental-Especies nativas de México de Flora y fauna silvestres- Categorías de riesgo y especificaciones para su inclusión, exclusión o cambio-Lista de especies en riesgo.

- Norma Oficial Mexicana Nom-060-Semarnat-1994, Que establece las especificaciones para mitigar los efectos adversos ocasionados en los suelos y cuerpos de agua por el aprovechamiento forestal.

- Norma Oficial Mexicana Nom-061-Semarnat-1994, Que establece las especificaciones para mitigar los efectos adversos ocasionados en la flora y fauna silvestres por el aprovechamiento forestal.

- Norma Oficial Mexicana Nom-062-Semarnat-1994, Que establece las especificaciones para mitigar los efectos adversos sobre la biodiversidad ocasionados por el cambio de uso del suelo de terrenos forestales a agropecuarios.

- Norma Oficial Mexicana Nom-126-Semarnat-2000, Por la que se establecen las especificaciones para la realización de actividades de colecta científica de material biológico de especies de flora y fauna silvestres y otros recursos biológicos en el territorio nacional.

- Norma Oficial Mexicana Nom-08-TUR-2002, Que establece los elementos a que deben sujetarse los guías generales y especializados en temas o localidades específicas de carácter cultural.

- Norma Oficial Mexicana Nom-09-TUR-2002, Que establece los elementos a que deben sujetarse los guías especializados en actividades específicas.

- Norma Oficial Mexicana Nom-011-TUR-2001, Requisitos de seguridad, información y operación que deben cumplir los prestadores de servicios turísticos de Turismo de Aventura.

5. DIAGNÓSTICO Y PROBLEMÁTICA DE LA SITUACIÓN AMBIENTAL

Dada su ubicación estratégica y la función clave que desempeña por su importancia hidrológica, biológica y social (en ese orden), el Pico de Tancítaro constituye un macizo volcánico de gran interés por la complejidad que acompaña a su problemática, que se refleja en una gran diversidad social y ecológica (Fuentes, 2000).

La particularidad de esta región se debe a que conjuga tres elementos fundamentales: un Área Natural Protegida con cierto grado de deterioro debido a la deforestación, alteración de la cubierta vegetal natural y erosión, lo anterior debido al incremento de los cultivos frutícolas (aguacate principalmente) en los últimos 20 años (Garibay y Bocco, 1999), además de una fuerte presencia de comunidades indígenas acompañadas de una ambigua tenencia de la tierra (componente étnico), la región presenta un gradiente altitudinal entre 2 mil 200 y 3 mil 860 metros sobre el nivel del mar, una topografía irregular y eventos volcánicos relativamente recientes.

Ecosistémico

La historia de la región está plagada de problemas y conflictos, tanto en cuestiones relacionadas con la tenencia de la tierra, como con el manejo de los recursos naturales, en especial el bosque; en el primer caso se pueden distinguir también dos niveles: los relativos a disputas ancestrales entre comunidades indígenas, principalmente de la Meseta Purépecha y las que tienen que ver con la expansión de la propiedad privada a costa de tierras de propiedad social (Paré *et al.,* 2008).

Después de la década de 1970 se sumaron grupos locales ambientalistas que han venido dando una fuerte batalla para mantener los bosques del área. Actualmente conviven en un equilibrio delicado figuras antagónicas en cuanto al uso de los recursos naturales.

En todo el territorio, el común denominador es la falta de agua, la cacería furtiva por propios y ajenos, el comercio ilegal de aves, el cambio de uso de suelo por la tala de árboles y la siembra de árboles frutales, principalmente aguacates; dichos elementos se describen a continuación:

Agua

La escasez de agua en las comunidades, la disminución de caudales y el agotamiento de manantiales son debidos a la desviación del agua para el riego de las plantaciones de aguacate principalmente.

Las y los agricultores, en especial productores de aguacate, son usuarios de distintos distritos de riego, explotan manantiales, extraen agua de pozos y canalizan escurrimientos. A pesar de todo esto, nadie ha mostrado la intención de cubrir algún tipo de compensación; uno de los argumentos es que la mayoría ya pagan cuotas a la Comisión Nacional de Agua (Paré *et al.*, 2008).

Por otra parte, el Pico de Tancítaro posee una gran importancia como área de recarga de acuíferos. En este sentido, Fuentes (2000) observó que las cañadas y los cauces de los escurrimientos poseen condiciones aún estables.

Sin embargo, en 1990 MacNeely y col. mencionaron que se tienen problemas de agotamiento de los mantos freáticos, de manantiales y de pérdida de la calidad de agua, lo cual redunda en un problema socioeconómico. De ahí la importancia de instrumentar la Ley de Aguas que permita ordenar los usos, el aprovechamiento y la restricción de su mejor uso, así como lo que contempla con respecto a la cultura del agua, la formación de los distritos de riego, la prevención y el control de la contaminación del agua.

Como es sabido, el ciclo del agua demanda una cubierta vegetal idealmente forestal que disminuya la evaporación, que retenga suelos y que provoque la filtración y el escurrimiento generando cauces de agua tanto superficiales como subterráneos, que de otra forma no existirían. Es decir, que sin árboles no hay agua (Conanp, 2008).

Aprovechamiento ilegal de fauna silvestre

La cacería furtiva por parte de las y los propietarios y poseedores en el Área Natural Protegida se realiza principalmente para el autoconsumo; las principales especies de mamíferos de importancia económica son: conejo, ardilla, tlacuache, liebre, armadillo y tejón. Sin embargo, se realiza sin saber si se están extrayendo especies amenazadas o en peligro de extinción. En estos casos, es importante la formación de comités de vigilancia para la cacería (relacionados entre sí), que cuenten con la información y organización necesarias y la relación de todos los permisos que se otorgan, para que a su vez esta actividad sea regulada internamente por las y los propietarios y poseedores.

La extracción de especies de aves canoras y de bello plumaje (jilgueros, primaveras y mulatos) en el Área Natural Protegida se desarrolla principalmente de manera ilegal. Estas especies son vendidas en los mercados de las localidades y cabeceras municipales de

Uruapan y Apatzingán, principalmente. Es necesaria la capacitación dirigida a las y los propietarios y poseedores sobre las especies amenazadas o en peligro de extinción que no pueden ser comercializadas.

Pérdida del suelo

Fuentes realizó en 2000, un estudio sobre la evaluación del deterioro del suelo en el área, con base en la cobertura vegetal, el tipo de relieve y la pendiente del área, lo que da como resultado que la degradación es mayor en las zonas que rodean el Pico de Tancítaro y menor hacia el centro de la estructura. En general la degradación va de mayor a menor según aumenta la altitud; se rompe entre los dos mil y tres mil metros sobre el nivel del mar debido a la existencia de bosques cerrados dentro del Anp.

En cuanto a la erosión, se aprecia una mayor influencia de los procesos de escorrentía en toda el área, siendo más conspicua en zonas con altitudes superiores a los tres mil metros sobre el nivel del mar, mientras que en las zonas de menor altitud y hasta los mil 300 metros sobre el nivel del mar existen procesos erosivos más acentuados como producto de un manejo más intenso orientado a la agricultura.

Por encima de los 3 mil 400 metros sobre el nivel del mar se observan evidencias de deterioro por erosión natural y antrópica. En el primer caso es frecuente la presencia de procesos periglaciares; mientras que en el segundo caso los procesos de compactación por bloques indican deterioro causado por incendios provocados y tala selectiva.

El Pico de Tancítaro funciona como una “isla forestal” y prácticamente ha perdido contacto con otras áreas boscosas. Lo anterior implica un serio peligro para la permanencia del área (Fuentes, 2000).

Cambio de uso de suelo

El incremento del cambio de uso de suelo en los bosques de pino-encino de Michoacán se debe principalmente a la conversión del bosque natural por plantaciones de aguacate (CEF *et al.*, 2007, en Barsimantov y Navia, 2008).

Por lo anterior, el Pico de Tancítaro presenta una dinámica de cambio de uso del suelo muy intensa, sobre todo a partir de la década de 1970, cuando las huertas de aguacate de la región empezaron a proliferar. También como parte de dicha problemática se manifiesta una intensa deforestación y un uso forestal maderable y no maderable intensivos que han provocado la degradación de los componentes del paisaje llevándolos a un punto crítico (García *et al.*, 2000). La tala de árboles y la extracción de árboles maderables incluyen principalmente las especies de pino, oyamel y encino.

Los cultivos de aguacate ocupan en la actualidad alrededor del 21 por ciento del total de la superficie del Área Natural Protegida en conjunto con cultivos de árboles frutales, como pera, durazno y ciruela, entre otros.

Fuentes (2000) concluyó que no es posible determinar el nivel de deterioro que presentan las coberturas de bosque. Sin embargo, se puede afirmar con certeza que toda el área se encuentra afectada por algún nivel de deterioro;

hacia las laderas orientales del área de influencia de la comunidad San Juan Nuevo Parangaricutiro los programas de aprovechamiento forestal han permitido un menor nivel de deterioro tanto actual como potencial; hacia las laderas norte, oeste y sur el avance de las huertas aguacateras ha desempeñado un papel determinante en la pérdida de cobertura vegetal original.

Aunque el cultivo del aguacate genera grandes ingresos económicos, los suelos pierden en los primeros años de cultivo sus nutrientes, debido a las necesidades de la planta para una buena producción; se desperdician grandes cantidades de agua por las inadecuadas técnicas de riego, los agroquímicos utilizados son perjudiciales tanto para el suelo como para la salud de la gente y se ha generado una gran pérdida de bosques, siendo ésta una de las más grandes contradicciones del cultivo, porque el aguacate es incapaz de reproducir por sí mismo las condiciones ambientales que necesita para su óptimo desarrollo y para lograr frutos de calidad (OET, 2010).

Es importante mencionar que el rápido cambio de uso de suelo en la región ha sido promovido o facilitado por tres cambios en las políticas públicas implementadas desde inicios de los años noventa, con la finalidad de llevar a cabo parte de las reformas estructurales de la economía de México, principalmente en el marco del Tratado de Libre Comercio: i) la reforma del Artículo 27 de la Constitución Mexicana; ii) la Ley Forestal de 1992; y, iii) algunas negociaciones comerciales entre Estados Unidos de América y México. Por un lado, la reforma del Artículo 27 permitió la posibilidad de que los ejidatarios obtengan certificados individuales y títulos en áreas comunales no forestales, generando un mercado de tierras, muchas de las veces en forma ilegal, que en los últimos años incluso han abarcado áreas forestales en que la misma ley tiene prohibida su parcelación (Navia, 2007).

Poco se ha documentado sobre las implicaciones de dimensión política y económica del cambio de uso de suelo en esta región, e incluso existen inconsistencias teóricas sobre las tasas de cambio (Navia, 2007).

Pérdida de cubierta forestal

El modelo de degradación del Pico de Tancítaro indica una dinámica centrípeta (de afuera del área hacia adentro) de manera dominante. Lo anterior se observa porque la mayor degradación se presenta fuera del área, empezando a evidenciarse zonas de invasión aguacatera sobre terrenos forestales y no sobre cultivos, como ha venido siendo la norma en los últimos años (Fuentes, 2000).

En las comunidades de abetos y pastos de altura se aprecian efectos de quemas intencionadas en pastos, además de la presencia de plagas de muérdago y gusano descortezador, que se encuentran en la parte más alta de *Pinus hartwegii,* mientras que en las comunidades de bosque mixto se aprecia la sustitución de bosque por plantaciones y cultivos, así como la extracción clandestina de madera (Fuentes, 2000).

La pérdida de cubierta vegetal original en la región ha traído como consecuencia la desaparición o desplazamiento de

especies de flora y fauna silvestres nativas, así como la erosión y desaparición de la cubierta fértil del suelo y su productividad, la afectación a los ciclos de nutrientes, la captación de carbono, las condiciones ambientales y el deterioro de la calidad de la masa forestal por la incidencia de plagas e incendios naturales e inducidos. Quizá uno de los problemas ambientales más graves asociados con la pérdida de la cubierta forestal del Pico de Tancítaro sea la pérdida de potencial en la captación e infiltración de agua (Conanp, 2008).

Los componentes básicos del paisaje, como el relieve, el suelo, la vegetación y el agua (escurrimientos), presentan indicios de deterioro que pudieran expresar el límite de estabilidad del sistema. Se aprecian diferencias sustanciales en los niveles de deterioro entre diversos sectores del Pico de Tancítaro, de tal manera que el deterioro no es homogéneo, sino localizado en los sitios que corresponden al área oriental, desde Paso de la Nieve hasta Zirimóndiro.

El manejo de los recursos del Área Natural Protegida debe responder a diversos criterios incluyendo principalmente consideraciones de las características ambientales del Anp. En este sentido resulta un ejemplo el sitio San Juan Nuevo por su manejo ambiental eficiente; es decir, en una superficie de 90 por ciento del territorio del Anp.

Demográfico y socioeconómico

La principal problemática socioeconómica a la que se enfrentan los habitantes dentro y fuera del Área Natural Protegida es a la falta de oportunidades, de proyectos productivos y de fuentes de empleo, lo que provoca la extracción ilegal de especies, la caza furtiva, la tala ilegal, el cambio de uso de suelo por actividades económicas y la existencia de otras actividades que presentan riesgos. Estas actividades humanas atentan contra la conservación de la biodiversidad; sin embargo, esto es producto de una alta presión social y económica en el área.

El aislamiento de algunas comunidades que presentan grandes carencias, donde las oportunidades de desarrollo y conservación son nulas, es otro problema que puede influir en el manejo del Anp, por lo que se deberán buscar alternativas para solucionarlas.

Los pequeños propietarios se dedican principalmente al cultivo de aguacate. En el APFF del Pico de Tancítaro, el 21 por ciento de la superficie está cubierta bajo este uso de suelo, ocasionando que diferentes servicios ambientales se vean afectados; tal es el caso del agua, debido a la contaminación por el uso de gran cantidad de insumos químicos para mantener este cultivo, así como la disminución del recurso, ya que el cultivo demanda una gran cantidad de agua para sobrevivir.

Estos problemas han sido ocasionados por actividades incompatibles, la ignorancia o mala organización y una visión netamente económica de las actividades productivas (Fuentes y Alvarado, 2006).

Es importante mencionar que existen conflictos de límites por los terrenos entre la comunidad indígena de San Salvador Combutzio y la Comunidad Indígena de San

Juan Nuevo Parangaricutiro, sobre todo en terrenos que están inmersos dentro del Área Natural Protegida, según los datos del Registro Agrario Nacional en su página electrónica (http://sig.ran.gob.mx).

Finalmente, podemos destacar que la mayor parte de la problemática demográfica y socioeconómica radica principalmente en la Zona de Influencia del Área Natural Protegida, ya que la población que habita al interior del área es escasa.

Presencia y coordinación institucional

En el Área Natural Protegida las instancias que han tenido mayor presencia son aquellas que han apoyado los distintos Programas Productivos, los municipios y los centros de investigación. La Comisión Nacional de Áreas Naturales Protegidas (Conanp) se sumó a este esfuerzo a partir de 2006, con la aplicación de Programas de Subsidio así como la gestión para la modificación de categoría del Área Natural Protegida.

Es importante que para el logro de los objetivos del Programa de Manejo del Área Natural Protegida participen los sectores social, gubernamental y científico, entre otros, para lo cual es importante la integración de un Consejo Asesor que funja como un espacio de participación de las poblaciones, además de ser un instrumento de consulta y asesoría para el seguimiento y la evaluación de las actividades dentro del Anp.

6. SUBPROGRAMAS DE CONSERVACIÓN

La operación, el manejo y la administración del Área de Protección de Flora y Fauna están encaminados a establecer un sistema de planificación que permita alcanzar los objetivos de conservación y manejo de los ecosistemas y sus elementos que alberga, manteniendo una presencia institucional permanente y contribuyendo a solucionar su problemática con base en labores de protección, manejo, gestión, investigación y difusión; todo ello en congruencia con los lineamientos de sustentabilidad que establecen el Plan Nacional de Desarrollo 2014-2018, el Programa Sectorial de Medio Ambiente y Recursos Naturales 2014-2018 y, el Programa Nacional de Áreas Naturales Protegidas 2014-2018.

Con base en la problemática existente y las necesidades del Área Natural Protegida, los subprogramas están enfocados a estructurar y planificar en forma ordenada y priorizada las acciones hacia donde se dirigirán los recursos, esfuerzos y potencialidades con los que cuenta la Conanp, para el logro de los objetivos de conservación del Área de Protección de Flora y Fauna. Dichos subprogramas están integrados en diferentes componentes, mismos que prevén objetivos específicos, metas y resultados esperados, así como las actividades y acciones que se deberán realizar, de lo que se advierte que todos y cada uno de los componentes tienen una estrecha interacción operativa y técnica, con lo que cada acción se complementa, suple o incorpora la conservación, la protección, la restauración, el manejo, la gestión, el conocimiento y la cultura como ejes rectores de política ambiental en el Área Natural Protegida.

Por lo anterior, la instrumentación se realiza a partir de la siguiente estructura:

- Subprograma de Protección
- Subprograma de Manejo
- Subprograma de Restauración
- Subprograma de Conocimiento
- Subprograma de Cultura
- Subprograma de Gestión

Los alcances de los subprogramas del presente Programa de Manejo se han establecido en relación con los periodos

en que las acciones deberán desarrollarse. El corto plazo (C) se refiere a un periodo de entre uno y dos años, el mediano plazo (M) es un periodo de tres a cuatro años, el largo plazo (L) se refiere a un periodo mayor a cinco años y la categoría de permanente (P) se asigna a las acciones o actividades que se deberán operar por plazos indefinidos.

Subprograma de protección

Este subprograma se refiere a la protección de los recursos del Área Natural Protegida y establece acciones para la protección del ambiente para asegurar la integridad de los elementos que conforman el ecosistema. Estas acciones se enfocan a la protección contra causas naturales y antropogénicas que han generado alteraciones y desequilibrios, tal es el caso de los incendios forestales, la ganadería extensiva, los aprovechamientos forestales, la cacería furtiva, las actividades turísticas y demás causas que pudiesen dañar el patrimonio natural de esta Área Natural Protegida.

Para ello, este subprograma plantea acciones de inspección, vigilancia, prevención de ilícitos, contingencias y protección contra especies invasoras y nocivas, para asegurar la continuidad de los procesos evolutivos en el Área de Protección de Flora y Fauna Pico de Tancítaro.

Objetivo general

Favorecer la permanencia y conservación de la diversidad biológica del Área de Protección de Flora y Fauna Pico de Tancítaro, a través del establecimiento y promoción de un conjunto de políticas y medidas para mejorar el ambiente y controlar el deterioro de los ecosistemas.

Estrategias

- Instrumentar acciones encaminadas a la conservación del ecosistema y sus elementos.
- Implementar medidas y políticas para mejorar el ambiente y controlar el deterioro de los ecosistemas a través de la prevención y vigilancia, en coordinación con las distintas dependencias federales y bajo la aplicación de la normatividad vigente y las Reglas Administrativas del presente instrumento.
- Promover la participación social en labores de protección y vigilancia.

Componente de mantenimiento de regímenes de perturbación y procesos ecológicos a gran escala

La ocurrencia de perturbaciones en el Área Natural Protegida no se ha identificado con exactitud; sin embargo, la mayoría de éstas suceden por fenómenos naturales extraordinarios o pueden ser provocadas por las actividades humanas.

En el APFFPT uno de los principales fenómenos que suceden de forma imprevista son los incendios forestales, pero resulta fundamental la identificación de éste y otros factores, así como su frecuencia natural, incluyendo su rango de variabilidad.

Objetivo específico

- Disminuir los efectos por perturbaciones naturales o antrópicas.

Meta y resultado esperado

- Contar en tres años con una estrategia para mitigar los efectos de las perturbaciones en el Anp.

Actividades* y acciones	Plazo
Mitigar los efectos de las perturbaciones	
Elaborar un diagnóstico y una base de datos georreferenciada de identificación y caracterización de sitios de perturbación, así como de los procesos que los originaron	M
Promover acciones que ordenen el cambio de uso de suelo en la Zona de Influencia del Área Natural Protegida	L
Establecer una estrategia para mitigar los efectos de las perturbaciones en el Área Natural Protegida, con base en los resultados del diagnóstico	M

*Las actividades se presentan en letra cursiva.

Componente de prevención, control y combate de incendios y contingencias ambientales

El origen de los incendios en el Área de Protección de Flora y Fauna Pico de Tancítaro es básicamente antropogénico, pues los disturbios naturales son escasos. El uso del fuego en las actividades agropecuarias y silvícolas son las causas más comunes de los incendios forestales, ya sea por negligencia o accidente, y la mayor parte de ellos pueden ser evitados, por lo que reviste una especial importancia el establecimiento de acciones adecuadas de prevención.

Los tipos de vegetación presentes en el Área Natural Protegida, tales como bosques de pino y asociaciones de pino-encino, son altamente susceptibles a ser afectados por los incendios, debido a que presentan condiciones altas de inflamabilidad propiciadas por los ambientes bajos de humedad durante la época de sequía, por lo que deberá valorarse la magnitud del incremento de materiales combustibles y tomar decisiones de manejo respecto a este material, así como establecer un sistema de seguimiento de la frecuencia y la intensidad de los incendios, con el fin de determinar los sitios con mayor probabilidad de siniestros y planear actividades de manejo de los mismos.

Objetivo específico

- Identificar, prevenir, reducir y mitigar el impacto de los incendios forestales y otras contingencias ambientales que impactan a los ecosistemas, derivados de fenómenos naturales y actividades humanas.

Metas y resultados esperados

- Contar con un programa anual de prevención, detección y control de incendios.

- Establecer anualmente al menos una brigada comunitaria para la prevención y combate de incendios.

Actividades* y acciones	Plazo
Elaborar un Programa de Manejo de fuego en el Área Natural Protegida	
Elaborar un diagnóstico y un mapa de los incendios forestales en el ANP, evidenciando los sitios de mayor riesgo	C
Elaborar una base de datos con información sobre incendios forestales del Área de Protección de Flora y Fauna Pico de Tancítaro	C
Formular un calendario de acción para la detección y atención de incendios en coordinación con la CONAFOR	C
Utilizar el sistema de monitoreo de puntos de calor de CONABIO para monitorear e identificar los sitios más susceptibles de incendios dentro del ANP y su Zona de Influencia	P
Conformar una brigada con voluntarios para la prevención y control de incendios	C
Identificar y dar mantenimiento a caminos y brechas cortafuego	C
Elaborar material de difusión para la prevención y el control de incendios	C
Realizar informes de contingencias ambientales atendidas	P
Colaborar con las instancias competentes en la atención de contingencias ambientales	
Participar en la estructura del sistema de protección civil para la atención de contingencias ambientales	P

*Las actividades se presentan en letra cursiva.

Componente de preservación e integridad de Zonas Núcleo y áreas frágiles y sensibles

La fragilidad ecológica está íntimamente relacionada con las características intrínsecas de cada ecosistema (e.i., riqueza, biodiversidad, resiliencia, endemismo y carácter relicto, entre otros) y se define como la susceptibilidad de los ecosistemas ante el impacto ocasionado por procesos naturales o acciones antropogénicas a que están expuestos (Chiappy, 1996).

El Área de Protección de Flora y Fauna Pico de Tancítaro contiene remanentes del bosque mesófilo de montaña, que dada su fragilidad ecológica han sido identificados como un objeto de conservación prioritario que requieren medidas reforzadas y especiales para su conservación y protección. Estos remanentes se componen de bosques de pino, encino, abies y asociaciones de pino-encino.

Objetivo específico

- Procurar la protección y recuperación de las áreas frágiles y sensibles presentes en el Área Natural Protegida.

Meta y resultado esperado

- Identificar al 100 por ciento las áreas frágiles o sensibles en el Área de Protección de Flora y Fauna Pico de Tancítaro.

Actividades* y acciones	Plazo
Conservar el bosque mesófilo de montaña (BMM)	
Identificar las áreas frágiles y sensibles presentes en el Área Natural Protegida y establecer criterios para darles prioridad de atención	C
Realizar delimitación de áreas en las que sea necesaria la recuperación del BMM	L
Establecer e implementar un programa para la protección y recuperación de las áreas frágiles y sensibles en el Anp	M
Controlar los impactos ambientales de las actividades humanas	
Impulsar un estudio de los impactos, la magnitud y las características de los impactos generados por actividades humanas en sitios frágiles y sensibles	M
Evitar que las actividades humanas generen disturbios en el BMM, con el fin de impedir estrés en las poblaciones silvestres	P

*Las actividades se presentan en letra cursiva.

Componente de protección contra especies exóticas invasoras y control de especies y poblaciones que se tornen perjudiciales

Los sistemas extensivos, tradicionales o convencionales de producción de ganado bovino constituyen una actividad económica que se realiza dentro del Área de Protección de Flora y Fauna Pico de Tancítaro; esta actividad, aunque se realiza a baja escala, afecta el renuevo de especies forestales, como el pino y el oyamel. Por otro lado, las masas forestales del Anp requieren vigilancia permanente, ya que son bosques susceptibles de presentar plagas debido al aprovechamiento forestal intensivo de abetos, por lo que hay presencia de plagas de muérdago y gusano descortezador, que si no se controlan pueden extenderse y afectar áreas de tamaño considerable.

Objetivos específicos

- Prevenir la introducción de especies exóticas invasoras mediante la ejecución de actividades de control y vigilancia a las y los usuarios.

- Identificar y evaluar, y en su caso erradicar, las poblaciones de las especies o ejemplares que se tornen perjudiciales mediante la participación de instituciones de investigación.

Metas y resultados esperados

- Contar a cinco años con una base de datos sobre la distribución que

guardan las especies invasoras y perjudiciales presentes en el ANP.

- Contar a cinco años con una estrategia de control y erradicación de especies invasoras y perjudiciales.
- Contar a cinco años con una estrategia de prevención de introducción de especies exóticas e invasoras.

Actividades* y acciones	Plazo
Aplicar un programa preventivo de introducción de especies exóticas invasoras y de control de especies que se tornen perjudiciales	
Solicitar la opinión de la Dirección General de Vida Silvestre, a fin de elaborar un estudio para obtener el diagnóstico de las especies exóticas invasoras y de especies o poblaciones que se tornen perjudiciales, su grado de afectación, las soluciones para su control y sus consecuencias a nivel socioeconómico y ecológico, en coordinación con las autoridades competentes	M
Promover la evaluación del impacto en la biodiversidad de las especies exóticas invasoras dentro del polígono del ANP	M
Solicitar a la Dirección General de Vida Silvestre el apoyo para realizar acciones específicas de control de las especies exóticas invasoras identificadas en el ANP	M
Formular un programa preventivo de introducción de especies exóticas invasoras, que incluya talleres informativos dirigidos a las comunidades aledañas y a las y los usuarios del ANP, en coordinación con las autoridades competentes	M
Elaborar y ejecutar un plan de acción para el control y erradicación de especies exóticas invasoras	M
Elaborar y ejecutar un plan de acción para el control y erradicación de especies que se tornen perjudiciales	M
Establecer mecanismos de coordinación con las autoridades competentes para la prevención de la introducción de especies exóticas y el control de especies que se tornen perjudiciales	P

*Las actividades se presentan en letra cursiva.

Componente de mitigación y adaptación al cambio climático

El cambio climático representa una amenaza creciente para el capital natural y humano del país. La escala y velocidad de las variaciones del clima nos obliga a tener un entendimiento de cómo estos cambios impactarán en las comunidades humanas, los ecosistemas y su biodiversidad.

En marzo de 2010, la Comisión Nacional de Áreas Naturales Protegidas presentó la Estrategia de Cambio Climático para Áreas Naturales Protegidas (ECCAP), la cual busca ser un instrumento dinámico que oriente las acciones y la toma de decisiones de la Conanp a niveles local, regional y nacional, posibilitando la concurrencia de recursos y apoyos de otras instituciones gubernamentales y académicas, así como de organizaciones civiles y sociales. Esta estrategia facilitará la articulación de los objetivos y metas del Programa Especial de Cambio Climático del Gobierno Federal, con el Programa Nacional de Áreas Naturales Protegidas y los Programas de Manejo de cada Anp.

El cambio climático genera fenómenos naturales, tales como huracanes, lluvias torrenciales, heladas, ondas de calor, inundaciones, entre otros, los cuales cuasan impactos adversos al ambiente, como sequía, humedad de suelos, desplazamiento de especies o la extinción de las mismas.

El Área de Protección de Flora y Fauna tiene la capacidad de contribuir a la mitigación del cambio climático a través de la conservación y el mantenimiento de los bienes y servicios que provee, por lo que es necesario definir acciones que mitiguen su efecto.

Objetivo específico

- Difundir información clara y precisa sobre la problemática y las estrategias para hacerle frente al cambio climático.

Metas y resultados esperados

- Contar a cinco años con las líneas base para identificar los efectos del cambio climático en los ecosistemas del Anp.

**Actividades* y acciones**	**Plazo**
Identificar los efectos del cambio climático en el ANP	
Promover estudios que permitan identificar especies indicadoras y vulnerables al cambio climático en el Área Natural Protegida	M
Procurar la conservación de la cobertura forestal a fin de contribuir a la mitigación de emisiones de gases efecto invernadero	P
Promover estudios de procesos ecológicos y dinámica de poblaciones del ANP a fin de identificar acciones que reduzcan su vulnerabilidad e incrementen su resiliencia ante el cambio climático	M
Realizar un diagnóstico para identificar las tierras degradadas y con bajo potencial productivo, e identificar los mecanismos para su reconversión	M
Promover la reconversión de tierras degradadas y con bajo potencial productivo en base a los resultados del diagnóstico	M
Contar con un programa de acción para enfrentar el cambio climático	
Impulsar un proyecto de apertura de mercados locales de bonos de carbono	L
Promover estudios para determinar la vulnerabilidad de los asentamientos humanos que se localizan dentro del Área Natural Protegida y su Zona de Influencia susceptibles a altos niveles de riesgo por la ocurrencia de eventos extraordinarios ligados al cambio climático	L
Definir medidas que puedan elevar la capacidad de respuesta natural ante eventos extraordinarios, como es en sitios específicos que requieran reforestarse, entre otros	C
Establecer acciones directas para apoyar el manejo forestal sustentable	M
Contribuir a la reducción de las emisiones de CO_2 derivado de quemas agrícolas, mediante la aplicación de un Programa de Manejo de Fuego	L
Fomentar la información a distintos actores	
Promover la educación e información a las comunidades y ejidos por medio de eventos informativos	P
Promover la participación local en las acciones de cambio climático, a fin de que se involucren activamente las y los usuarios del Área Natural Protegida en las actividades definidas en el presente componente	P

*Las actividades se presentan en letra cursiva.

Componente de inspección y vigilancia

La población de las comunidades que rodean el Área Natural Protegida ha aprovechado históricamente de manera constante los recursos naturales que la rodean, lo que ha contribuido a la disminución de los recursos forestales maderables y no maderables, la flora, la fauna y el volumen de agua. Ante esto, es imprescindible realizar acciones preventivas y correctivas específicas para la inspección, la vigilancia y el registro de ilícitos que permitan conservar el ANP como refugio de flora y fauna, y mantener la captación de agua y los hábitat específicos que alberga.

La inspección y la vigilancia son mecanismos necesarios para la conservación de los recursos naturales del ANP y para asegurar la continuidad de las actividades de forma sustentable. En este componente se establecen las actividades y acciones que se implementarán para hacer cumplir las regulaciones sobre el uso del Área Natural Protegida, así como las medidas necesarias para procurar el bienestar de las y los usuarios.

Es importante instrumentar operativos de vigilancia de las diferentes actividades que se desarrollen dentro del ANP. Para que este mecanismo sea efectivo es necesario que se ejecuten acciones coordinadas con otras instituciones involucradas en las labores de inspección y vigilancia del Área Natural Protegida.

Objetivos específicos

- Proteger y conservar los ecosistemas del Área de Protección de Flora y Fauna Pico de Tancítaro, su biodiversidad y sus recursos mediante la actuación oportuna ante los ilícitos ambientales a través de la aplicación de la normatividad ambiental vigente, la vigilancia participativa comunitaria y la coordinación institucional, conforme a las disposiciones legales aplicables.

- Coadyuvar en la conservación de los recursos naturales a través de la planificación, instrumentación y ejecución de medidas de inspección y vigilancia, complementadas con trabajos de difusión para promover la participación ciudadana.

Metas y resultados esperados

- Contar en tres años con un Programa Anual de Inspección y Vigilancia.

- Contar con al menos dos comités de vigilancia participativa de las comunidades del Área Natural Protegida.

Actividades* y acciones	Plazo
Elaborar un programa anual de inspección y vigilancia con las instancias de competencia en el tema, como Profepa y otras	
Realizar un diagnóstico sobre los ilícitos ambientales	C
Proponer acciones concretas para la disminución de los ilícitos ambientales en conjunto con las autoridades competentes	C
Generar un calendario de inspección y vigilancia con las autoridades competentes, con base en los resultados del diagnóstico	C
Coadyuvar en la integración de comités de vigilancia participativa	
Realizar reuniones de trabajo con las comunidades sobre irregularidades ambientales al interior del Área de Protección de Flora y Fauna Pico de Tancítaro y sobre la importancia de conformar comités voluntarios de vigilancia	C
Gestionar ante la Procuraduría Federal de Protección al Ambiente la capacitación y acreditación de comités voluntarios de vigilancia	C
Consolidar el Programa de Inspección y Vigilancia	
Dar seguimiento al Programa de Inspección y Vigilancia en conjunto con la Profepa, así como gestionar su renovación anual	P
Reportar a la Procuraduría Federal de Protección al Ambiente y a las autoridades competentes los posibles ilícitos ambientales detectados durante los recorridos regulares del personal del Área de Protección de Flora y Fauna Pico de Tancítaro	P
Dar seguimiento a los procedimientos establecidos por la Profepa	P

*Las actividades se presentan en letra cursiva.

Subprograma de manejo

El manejo identifica e integra las acciones y actividades que garantizarán la permanencia a largo plazo de los procesos ecológicos esenciales, los ecosistemas, los hábitat y las especies de flora y fauna silvestres que el Área de Protección de Flora y Fauna Pico de Tancítaro alberga.

Diversas actividades antropogénicas y fenómenos naturales han impactado los ecosistemas y la biodiversidad del Anp, por lo que se busca que con el conjunto de estrategias, programas y regulaciones que establece este subprograma se contribuya a la efectividad de las acciones de conservación, protección, desarrollo sustentable, investigación y recreación en el Área Natural Protegida.

Objetivo general

Establecer políticas, estrategias y programas con el fin de determinar actividades y acciones orientadas al cumplimiento de los objetivos de conservación, protección, restauración, capacitación, educación, aprovechamiento sustentable de los recursos naturales y recreación del Área de Protección de Flora y Fauna Pico de Tancítaro, a través de proyectos alternativos y la promoción de actividades de desarrollo sustentable.

Estrategias

- Impulsar el uso sustentable de los recursos naturales del Área de Protección de Flora y Fauna Pico de Tancítaro.
- Promover la regulación de las actividades productivas dentro del Área de Protección de Flora y Fauna Pico de Tancítaro y su reconversión productiva hacia sistemas más amigables con el ambiente.
- Fomentar la realización de inventarios forestales de las principales especies aprovechables (pinos y encinos).
- Promover apoyos para la realización de proyectos productivos sustentables en el Área Natural Protegida.
- Promover el Pago por Servicios Ambientales en el Área Natural Protegida y en su Zona de Influencia.

Componente de actividades productivas alternativas y tradicionales

Una de las principales actividades económicas en la región del Pico de Tancítaro es el cultivo del aguacate; este sistema de uso del suelo ocasiona que diferentes servicios ambientales, como es el caso del agua, se vea afectado, debido a la contaminación por el uso de insumos químicos y a la disminución del recurso, ya que el cultivo demanda una gran cantidad de agua para sobrevivir.

Esta práctica es la que ha promovido los cambios de uso de suelo más fuertes en la región en las últimas décadas (Fuentes, 2000). Por ello será importante implementar programas de reconversión de los sistemas de producción tradicionales por alternativas de producción sustentable y oportunidades de generación de ingresos, bienes y servicios, de tal forma que los ecosistemas, tipos de vegetación, suelos, flora, fauna, cuerpos de agua, atractivos turísticos y otros atributos naturales sean aprovechados de forma sustentable.

Además del fomento de oportunidades de proyectos productivos para las y los habitantes dentro del Anp y su Zona de Influencia es de vital importancia obtener beneficios ambientales y sociales a favor de la conservación del área.

Objetivo específico

- Favorecer el desarrollo económico de la población del Área Natural Protegida y su Zona de Influencia a través de la promoción de actividades productivas alternativas ambientalmente compatibles con los objetivos de conservación.

Metas y resultados esperados

- Contar en tres años con un diagnóstico de las actividades productivas alternativas que podrían realizarse en el Área Natural Protegida y su Zona de Influencia.
- Implementar al menos un proyecto productivo alternativo por año.

Actividades* y acciones	Plazo
Identificar las actividades productivas alternativas en el ANP	
Elaborar y difundir un diagnóstico de actividades productivas tradicionales realizadas y potenciales a realizarse en el Área Natural Protegida	M
Promover actividades productivas alternativas sustentables para la Zona de Influencia	
Impulsar la diversificación productiva afines con los objetivos de conservación	M
Gestionar Programas de Subsidio que favorezcan la implementación de actividades productivas alternativas, con la autoridades competentes	M
Realizar gestiones antes las autoridades competentes, para que los diferentes programas productivos y de uso de suelo actual que se aplican al interior del ANP se desarrollen en apego a los objetivos de ésta	P

*Las actividades se presentan en letra cursiva.

Componente de manejo y uso sustentable de agroecosistemas y ganadería

Los cambios de cobertura de bosque a cultivos permanentes fuera del Área Natural Protegida ejercen una presión sobre los bosques del Área de Protección de Flora y Fauna Pico de Tancítaro. Es importante realizar actividades y gestiones con el fin de detener la tendencia del incremento del cambio de uso de suelo en la Zona de Influencia, a efectos de disminuir el impacto en el Área Natural Protegida.

La ganadería extensiva es una actividad generalizada y poco redituable pero que culturalmente se encuentra muy arraigada y dañina en zonas donde hay regeneración natural de la vegetación; dentro del Área Natural Protegida la ganadería aún no es un gran problema; sin embargo, es importante regular esta práctica a tiempo para que no sea un problema significativo en un futuro.

Objetivo específico

- Contener los procesos erosivos provocados por las actividades agropecuarias y ganaderas, disminuyendo la superficie bajo esos usos en el Área de Protección de Flora y Fauna Pico de Tancítaro.

Metas y resultados esperados

- Contar a cinco años con una estrategia de recuperación de áreas degradadas por uso agropastoril.
- Generar acciones para la reconversión a sistemas agrosilvopastoriles.

Actividades* y acciones	Plazo
Actualizar la información en materia agropecuaria	
Elaborar un diagnóstico agropecuario y ganadero en el Anp	C
Promover la participación comunitaria para la aplicación de técnicas agropecuarias sustentables	M
Coordinar acciones interinstitucionales que promuevan el desarrollo de proyectos alternativos a la agricultura y ganadería	C
Aplicar sistemas agrosilvopastoriles y modelos agrícolas sustentables que contribuyan a disminuir las áreas de uso ganadero	
Promover el desarrollo de proyectos agroecológicos	M
Difundir la inconveniencia del uso de agroquímicos	C

*Las actividades se presentan en letra cursiva.

Componente de manejo y uso sustentable de ecosistemas terrestres y recursos forestales

Los servicios ambientales que brindan las comunidades vegetales en el APFFPT son de vital importancia, por lo que para garantizar la continuidad de estos servicios ambientales es necesario un manejo adecuado de las comunidades vegetales y de los recursos naturales presentes en el área, con el fin de asegurar la continuidad de los procesos biológico-ecológicos que se llevan a cabo.

La explotación de madera es una actividad que se realiza en todo el país, ya que genera fuentes de empleo para muchas de las comunidades que habitan las áreas aledañas a estos sitios explotados; sin embargo, esta actividad se realiza mayormente de forma ilegal y es una de las actividades económicas preponderantes en el Área de Protección de Flora y Fauna Pico de Tancítaro.

Dada la importancia de esta actividad para el desarrollo regional, es necesario garantizar el mantenimiento de estos beneficios a largo plazo, por lo que este componente contiene acciones enfocadas al cumplimiento de esa meta y se propone el desarrollo de un proyecto para conformar una unidad para la conservación y aprovechamiento sustentable de los recursos naturales en el Área Natural Protegida.

Objetivo específico

- Mantener los ecosistemas forestales y sus procesos ecológicos mediante la generación de un Programa de Manejo Forestal con fines de conservación y aprovechamiento sustentable dentro del Área de Protección de Flora y Fauna Pico de Tancítaro y su Zona de Influencia.

Meta y resultado esperado

- Contar en cinco años con un Programa de Manejo Forestal y uso sustentable de los ecosistemas terrestres con el fin de mantener los servicios ambientales que brinda el Anp.

Actividades* y acciones	Plazo
Elaborar un Programa de Manejo de los recursos forestales	
Identificar los aprovechamientos forestales realizados en el Área Natural Protegida	M
Elaborar un listado de actividades y permisionarios actuales y potenciales	C
Fomentar la sensibilización y organización de las comunidades a través de la capacitación sobre el manejo y conservación de los recursos forestales	P
Promover ante las autoridades competentes la regulación de las actividades de aprovechamiento forestal	P
Promover el establecimiento de un vivero de producción de especies nativas para reforestar las zonas de aprovechamiento forestal	L

*Las actividades se presentan en letra cursiva.

Componente de manejo y uso sustentable de vida silvestre

El Área de Protección de Flora y Fauna Pico de Tancítaro posee una gran diversidad de paisajes que se expresa en la presencia de diversos tipos de vegetación, la riqueza de especies y endemismos que permiten considerar al ANP como un territorio de alta importancia biogeográfica por ser una zona de relicto o refugio de un alto número de especies de animales y vegetales, algunas de ellas consideradas dentro de la NORMA OFICIAL MEXICANA NOM-059-SEMARNAT-2010, Protección ambiental-Especies nativas de México de flora y fauna silvestres-Categorías de riesgo y especificaciones para su inclusión, exclusión o cambio-Lista de especies en riesgo.

Estas características tan especiales le confieren un gran valor regional al Área y la permanencia de estos recursos dependerá en gran medida de la regulación para su aprovechamiento sustentable y su adecuado manejo.

Por lo anterior, este componente busca el uso sustentable de la vida silvestre presente en el Área Natural Protegida y su Zona de Influencia para consolidar su conservación.

OBJETIVO ESPECÍFICO

- Fomentar la conservación y protección de la vida silvestre y sus procesos biológicos mediante el mantenimiento de las condiciones naturales de los ecosistemas.

METAS Y RESULTADOS ESPERADOS

- Fomentar el establecimiento de UMA para el manejo y aprovechamiento de la vida silvestre en el ANP.

- Contar a cinco años con mecanismos de seguimiento, monitoreo y evaluación que complementen los planes de manejo de las UMA, a fin de garantizar que las especies aprovechadas mantengan y/o recuperen sus poblaciones naturales.

Actividades* y acciones	Plazo
Facilitar la participación de las comunidades locales en el manejo y conservación de la vida silvestre	
Impulsar programas de recuperación para especies de vida silvestre que se encuentren dentro de alguna categoría de riesgo	P
Impulsar el establecimiento de UMA para el manejo y aprovechamiento de la vida silvestre	
Fomentar la capacitación a las comunidades para el manejo adecuado y aprovechamiento sustentable de la vida silvestre	C

*Las actividades se presentan en letra cursiva.

Componente de mantenimiento de servicios ambientales

La preservación de los recursos naturales en el Área de Protección de Flora y Fauna Pico de Tancítaro es de vital importancia, ya que contribuye a la producción de bienes y servicios ambientales, de los cuales dependen directamente las poblaciones locales.

Los ecosistemas forestales brindan servicios ambientales: de manera natural o por medio del manejo sustentable de los recursos forestales, tales como la provisión del agua en calidad y cantidad; la captura de carbono de contaminantes y componentes naturales; la generación de oxígeno; el amortiguamiento del impacto de los fenómenos naturales; la modulación o regulación climática; la protección de la biodiversidad de los ecosistemas y formas de vida; la protección y recuperación de suelos; el paisaje y la recreación, entre otros.

El Área de Protección de Flora y Fauna Pico de Tancítaro contiene los ecosistemas mejor conservados de la zona, que a su vez conforman las masas forestales y las principales cuencas hidrográficas de la región. Sus bienes y servicios ambientales tienen un alto valor para las y los habitantes locales o colindantes, así como para las poblaciones y sus actividades en el ámbito regional. Los bosques que se localizan en este macizo montañoso conforman paisajes de gran valor escénico y gran atractivo turístico, son refugio de flora y fauna; y dada la importancia que estas montañas tienen en los contextos regional y nacional, fueron integradas al Programa de las 60 Montañas Prioritarias de la Conafor, abriendo la posibilidad de beneficiar directamente a las y los dueños y poseedores del bosque por Pago por Servicios Ambientales.

Objetivo específico

- Establecer estrategias de conservación que permitan mantener en estado óptimo los recursos naturales del Anp mediante la implementación, validación y sistematización de acciones que contribuyan a la aplicación de mecanismos de valorización de los servicios ambientales de la región.

Metas y resultados esperados

- Impulsar esquemas de pago por Servicios Ambientales en el Anp.

- Contar a cinco años con un estudio de los bienes y servicios ambientales que brinda el Área de Protección de Flora y Fauna Pico de Tancítaro, su valor económico y ambiental y su influencia en los ámbitos local y regional.

Actividades* y acciones	Plazo
Generar esquemas de Pago por Servicios Ambientales	
Gestionar con los tres niveles de gobierno y Organizaciones de la Sociedad Civil (OSC) nacionales e internacionales un esquema de Pago por Servicios Ambientales	M
Impulsar un programa comunitario para la adopción de tecnologías de bajo consumo de agua	P
Elaborar un patrón de las y los dueños y poseedores de terrenos con bosques en buen estado de conservación	M
Promover ante la Conafor el Pago por Servicios Ambientales a las y los dueños y poseedores que cumplan con los requisitos para tal fin	C
Diagnóstico sobre los servicios ambientales en el Área Natural Protegida	
Promover la elaboración de un estudio sobre los servicios ambientales con los que cuenta el Anp, así como su valor económico e importancia ambiental	M

*Las actividades se presentan en letra cursiva.

Componente de turismo, uso público y recreación al aire libre

La abundancia, la diversidad de especies y los ecosistemas que presenta el Área de Protección de Flora y Fauna Pico de Tancítaro ofrecen una amplia gama de paisajes y escenarios que poseen un elevado atractivo natural propicio para el desarrollo del turismo, el cual puede desempeñar un papel muy importante en la diversificación de las actividades productivas de la región y estimular el crecimiento económico para mejorar los niveles de vida de las poblaciones locales.

El presente componente tiene como objetivo establecer los lineamientos para el desarrollo de un turismo de bajo impacto ambiental que asegure la sostenibilidad a largo plazo de los recursos naturales, además de promover la participación de las comunidades en la conservación, la preservación y el uso sustentable de los recursos naturales.

Objetivo específico

- Promover el desarrollo de turismo sostenible y actividades de ecoturismo fomentando la participación de las comunidades aledañas al Anp.

Meta y resultado esperado

- Lograr que el 60 por ciento de los proyectos de turismo de bajo impacto en el Área Natural Protegida se desarrollen con la participación de las comunidades.

Actividades* y acciones	Plazo
Evaluar y ordenar las actividades turísticas en el Área de Protección de Flora y Fauna Pico de Tancítaro	
Impulsar estudios para la implementación de proyectos de turismo de bajo impacto ambiental	C
Identificar con precisión las áreas destinadas al uso turístico de bajo impacto acorde con la zonificación del ANP	C
Impulsar la realización de estudios relacionados a la capacidad de carga, con el fin de establecer el límite de número de visitantes y el tiempo de permanencia en el Área de Protección de Flora y Fauna Pico de Tancítaro	M
Promover la consolidación de organizaciones comunitarias en el desarrollo del turismo de bajo impacto ambiental	M

*Las actividades se presentan en letra cursiva.

Subprograma de restauración

La recuperación de ambientes degradados es una preocupación creciente en nuestros días, pues la acelerada destrucción de los recursos naturales ha provocado grandes alteraciones en los procesos ecológicos. Actualmente, la restauración ecológica resulta ser un elemento clave en la conservación de los ecosistemas y su biodiversidad.

El Área de Protección de Flora y Fauna Pico de Tancítaro ha estado sujeta a la acción permanente de agentes que la deterioran, como son los incendios forestales, las plagas, el pastoreo sin control, la tala clandestina y el cambio de uso del suelo, los cuales han reducido considerablemente la cobertura boscosa del ANP.

Lograr la restauración de las áreas degradadas es, sin duda, una de las acciones de mayor prioridad para la administración del ANP.

Este subprograma tiene la finalidad de desarrollar las acciones dirigidas a lograr la restauración ecológica de diversas áreas afectadas en el Área de Protección de Flora y Fauna Pico de Tancítaro, así como la recuperación de especies de flora y fauna vulnerables y la conectividad de los ecosistemas.

Objetivo general

Recuperar y restablecer las condiciones ecológicas previas a las modificaciones causadas por las actividades humanas o fenómenos naturales, permitiendo la continuidad de los procesos naturales en los ecosistemas del Área de Protección de Flora y Fauna Pico de Tancítaro.

Estrategias

- Identificar los ambientes que deben ser restaurados, cuantificando las superficies afectadas y dando prioridad a las áreas críticas.

- Desarrollar proyectos de investigación para contar con un diagnóstico de los ecosistemas degradados.

- Fomentar la participación de la comunidad en el proceso de restauración ecológica.

- Establecer un plan de recuperación y manejo del suelo y conservación del agua en el Área de Protección de Flora y Fauna Pico de Tancítaro.

Componente de conectividad y ecología del paisaje

La mayor actividad económica de la población que incide en el Área de Protección de Flora y Fauna Pico de Tancítaro implica principalmente el cultivo del aguacate. Este régimen de uso de suelo ha ocasionado que diferentes servicios ambientales se vean perturbados por la deforestación o por los incendios inducidos que permiten el cambio de uso de suelo; esta actividad ha creado en gran medida parches de características diferentes en el paisaje boscoso propio del ANP, fenómeno que puede generar el aislamiento de los procesos ecológicos y evolutivos, por lo que mantener la conectividad ambiental del Área Natural Protegida es fundamental.

Objetivo específico

- Evitar el deterioro ambiental producido por interrupciones en la conectividad, el flujo genético y la integridad mediante la prevención y restauración de los ecosistemas del Área de Protección de Flora y Fauna Pico de Tancítaro.

Meta y resultado esperado

- Contar a cinco años con un plan de acción para la recuperación de las características de los paisajes afectados.

Actividades* y acciones	Plazo
Establecer las estrategias adecuadas que promuevan la conservación, el mantenimiento de la conectividad y el flujo genético entre los ecosistemas y la integridad del paisaje	
Realizar un diagnóstico de los paisajes transformados por actividades humanas o naturales en el ANP y que son susceptibles de conectividad	M
Establecer las medidas de prevención para evitar y reducir la fragmentación del ecosistema derivado de los impactos generados por las actividades antropogénicas	C
Crear una sinergia interinstitucional	
Impulsar acciones a nivel regional para mejorar la conectividad entre los ecosistemas y asegurar el mantenimiento de los bienes y servicios que presta el ANP	M

*Las actividades se presentan en letra cursiva.

Componente de recuperación de especies en riesgo

El Área de Protección de Flora y Fauna Pico de Tancítaro es considerada como de alta importancia biológica nacional e internacional debido a la riqueza de especies y a los endemismos que la componen. Es una zona de relicto o refugio de especies de flora y fauna al encontrarse un alto número de especies dentro de la Norma Oficial Mexicana Nom-059-Semarnat-2010, Protección ambiental-Especies nativas de México de flora y fauna silvestres-Categorías de riesgo y especificaciones para su inclusión, exclusión o cambio-Lista de especies en riesgo, a consecuencia, entre otros factores, de su condición de relieve-isla. En el Área de Protección de Flora y Fauna Pico de Tancítaro sobreviven especies raras y/o endémicas, además de que presenta tránsito o estancia de especies migratorias (Fuentes y Alvarado, 2006).

En la actualidad, en el Área Natural Protegida, la estabilidad poblacional de algunas especies que se ubican en la norma antes citada se encuentra amenazada debido a actividades antropogénicas no reguladas, como la cacería furtiva y a fenómenos naturales. Esto hace prioritaria la necesidad de desarrollar acciones que contribuyan a la conservación, el manejo, la recuperación y la restauración del hábitat de estas especies.

Objetivo específico

- Establecer el Programa de Acción para la Conservación de Especies (PACE) en riesgo para mantener poblaciones viables de especies de flora y fauna.

Meta y resultado esperado

- Contar a tres años con un protocolo de acción para la conservación de especies en riesgo en el Área Natural Protegida.

**Actividades* y acciones**	**Plazo**
Fomentar la recuperación y conservación de las especies en riesgo	
Elaborar y ejecutar un Programa de Acción para la Conservación de Especies para la recuperación y conservación de especies prioritarias presentes en el Anp	L
Identificar las principales amenazas que enfrentan las especies en alguna categoría de riesgo	C

*Las actividades se presentan en letra cursiva.

Componente de conservación del agua y el suelo

El Área de Protección de Flora y Fauna Pico de Tancítaro se localiza en el sistema de la Cuenca del Balsas. Los ecosistemas que conforman el Área Natural Protegida son fuente esencial para la captación de agua y la recarga de los mantos acuíferos en la región, proporcionan un importante servicio ambiental a las comunidades circundantes y permiten el adecuado desarrollo de los procesos ecológicos. Sin embargo, las alteraciones negativas, como la pérdida de la cubierta vegetal originada por incendios forestales, la tala clandestina y las actividades agrícolas, que incluye la contaminación del agua y el suelo por productos químicos, influyen en la disminución de la captación de este vital recurso. A fin de contrarrestar su deterioro, es necesario implementar estrategias y acciones orientadas a la reforestación, recuperación y conservación del suelo y el agua.

Objetivo específico

- Conservar los suelos y el agua como elementos clave para mantener los procesos ecológicos del Área Natural Protegida mediante acciones de manejo.

Meta y resultado esperado

- Contar a cinco años con una estrategia de conservación de suelo y agua que disminuya la degradación ocasionada por fenómenos naturales y actividades humanas.

Actividades* y acciones	Plazo
Establecer una estrategia de conservación de suelo y agua	
Identificar y caracterizar los puntos que requieren atención prioritaria para la conservación del suelo y el agua	C
Establecer e impulsar medidas que permitan mitigar los problemas de pérdida de suelo en puntos críticos del Área, para evitar la erosión	M
Coordinar con la Conagua el manejo, la extracción y la disponibilidad de agua	M
Difundir la importancia del recurso agua y el cuidado de la cuenca, así como la promoción de técnicas sobre el uso y manejo sustentable de este recurso en coordinación con instituciones involucradas en el tema	P, M
Impulsar la generación y aplicación de técnicas de uso y manejo sustentable del suelo y el agua, en coordinación con instituciones involucradas en el tema	M

*Las actividades se presentan en letra cursiva.

Componente de restauración de ecosistemas

Como se ha mencionado anteriormente, dada la ubicación estratégica del Anp y la función clave que desempeña por su importancia hidrológica, biológica y social (en ese orden) y la compleja biodiversidad que alberga, es un reflejo de la gran variabilidad paisajística con la presencia de relieves volcánicos muy diversos, además de que cuenta con una superficie boscosa que conforma un sistema de cobertura importante para el secuestro de carbono y la regulación del clima regional.

El grado de afectación de los ecosistemas en el área ha sido principalmente por causas antropogénicas (tala, siembra de árboles frutales y cacería furtiva, entre otras) y por causas naturales, como la erupción del Volcán Paricutín en 1943 y los incendios forestales ocurridos dentro y fuera del área.

Uno de los principales factores que contribuyen al deterioro o pérdida de la cubierta vegetal es el cambio de uso de suelo, principalmente para la agricultura y la ganadería.

Estas actividades generan impactos ambientales negativos que se traducen en la pérdida del hábitat, la erosión del suelo y la afectación a otros servicios ambientales, lo cual conlleva graves problemas sociales, económicos y ecológicos. Es necesario establecer acciones encaminadas a la reforestación y restauración efectiva de las áreas afectadas, con miras a la recuperación de ecosistemas que sustentan la biodiversidad del área.

Objetivo específico

- Recuperar los ecosistemas que han sido dañados y modificados por fenómenos naturales o por actividades humanas mediante actividades de rehabilitación.

Meta y resultado esperado

- Recuperar en un 20 por ciento los ecosistemas que hayan sufrido algún impacto negativo derivado de las actividades antropogénicas o fenómenos naturales mediante trabajos de recuperación.

Actividades* y acciones	Plazo
Identificar las zonas alteradas que requieran recuperación	
Promover la realización de estudios que permitan identificar las características ecológicas de los ecosistemas originales del Área de Protección de Flora y Fauna Pico de Tancítaro, a fin de contar con un punto de referencia en las acciones de recuperación de los ecosistemas	M
Realizar un estudio de evaluación del estado de conservación del Área Natural Protegida que identifique los ecosistemas que requieren acciones de restauración, así como las actividades necesarias para la recuperación de los ecosistemas	M
Aplicar los métodos de recuperación de los ecosistemas	
Impulsar la recuperación de ecosistemas críticos mediante actividades mecánicas necesarias para la preservación de las especies y procesos ecológicos	M
Realizar programas de restauración con especies nativas	P

*Las actividades se presentan en letra cursiva.

Subprograma de conocimiento

La investigación que se desarrolla dentro de un Área Natural Protegida es la base para tomar decisiones que conduzcan a un manejo adecuado de los recursos naturales y así lograr su restauración, protección y conservación. Por otro lado permite explicar los fenómenos que ocurren e inciden en los recursos naturales.

El desarrollo de este subprograma en el Área de Protección de Flora y Fauna Pico de Tancítaro permitirá vincular esfuerzos de colaboración con diversas instituciones académicas y guiar las líneas importantes de investigación, que deberán aportar información y elementos objetivos que sirvan como base para apoyar las Reglas de uso del área, el uso sustentable de los recursos y la evaluación de los esfuerzos de manejo.

Objetivo general

Generar, rescatar y divulgar conocimientos, prácticas y tecnologías, tradicionales o nuevas que permitan la preservación, la toma de decisiones y el aprovechamiento sustentable de la biodiversidad del Área de Protección de Flora y Fauna Pico de Tancítaro.

Estrategias

- Promover entre las instituciones de educación superior la investigación científica, que permita conocer, evaluar, proteger y manejar la biodiversidad del Área Natural Protegida, en especial las especies catalogadas bajo alguna categoría de riesgo.
- Establecer líneas de investigación y monitoreo dentro del Área Natural Protegida acordes a los lineamientos

del Programa de Manejo y con las necesidades del Área, que permitan contar con información oportuna para la planeación, implementación y evaluación de los procesos.

- Plantear acciones que permitan contar con los elementos técnicos y científicos para el adecuado monitoreo, estudio y protección de la biodiversidad, así como del uso de los recursos naturales.

- Crear una base de datos de las investigaciones realizadas en el Área de Protección de Flora y Fauna Pico de Tancítaro, relacionadas con los ecosistemas y su biodiversidad.

Componente de fomento a la investigación

En la región del Pico de Tancítaro se han desarrollado diversos estudios, como los realizados por Fuentes y Alvarado. Sin embargo, aún es necesario generar estudios que apoyen el desarrollo de las localidades del Área, y den como resultado cambios positivos en el manejo y/o la planeación de recursos presentes en el Anp.

Objetivo específico

- Fomentar, promover e incrementar los conocimientos básicos y aplicados de las características y funcionamiento de los ecosistemas; así como de sus recursos y su fragilidad mediante la realización de proyectos de investigación que aporten información relevante para la toma de decisiones del Área de Protección de Flora y Fauna Pico de Tancítaro.

Meta y resultado esperado

- Contar a cinco años con la línea base de investigación para el Anp.

**Actividades* y acciones**	**Plazo**
Establecer las líneas prioritarias de investigación para el Área Natural Protegida	
Identificar las necesidades de conocimiento del Área Natural Protegida	C
Mantener actualizada una base de datos sobre la información científica generada en el Área Natural Protegida y su Zona de Influencia por las investigaciones efectuadas	P
Establecer convenios interinstitucionales para ampliar el campo de investigación	
Establecer convenios y fomentar la participación de centros de investigación, universidades, OSC, gobiernos municipal, estatal y federal, para la realización de trabajos de investigación que completen el conocimiento de aspectos biológicos del Anp	C

*Las actividades se presentan en letra cursiva.

Componente de inventarios y monitoreo ambiental y socioeconómico

La integración, la sistematización y el seguimiento del conocimiento que se genere para el Área de Protección de Flora y Fauna Pico de Tancítaro será la base para la optimización de los recursos financieros y de las investigaciones en cuanto a sus alcances e impacto tanto a los ecosistemas como a las comunidades presentes en el Área Natural Protegida.

Con estas herramientas se podrá dar seguimiento al estado de salud o condición de un ecosistema, recurso o comunidad y su situación previa y posterior a un evento ambiental, como un incendio, la situación de una actividad productiva y un impacto antropogénico.

Este tipo de información permitirá proyectar, por medio de planteamientos científicos, escenarios futuros de los recursos y ecosistemas.

Objetivos específicos

- Establecer las bases para el desarrollo de un monitoreo ambiental que permita reconocer cambios temporales y/o espaciales en la estructura de las comunidades biológicas y en la calidad de los recursos abióticos.

- Identificar los fenómenos sociales de la región que inciden o pudieran incidir directamente en los objetivos del Área Natural Protegida.

Metas y resultados esperados

- Contar a cinco años con la línea base de monitoreo ambiental del Anp.

- Contar a cinco años con un programa de monitoreo permanente para los impactos derivados de los factores ambientales, sociales y económicos que incidan en el Área Natural Protegida.

**Actividades* y acciones**	**Plazo**
Establecer sistemas de monitoreo	
Impulsar con las instituciones de investigación y académicas, la actualización del inventario de especies de flora y fauna del Anp	L
Elaborar un protocolo de monitoreo para flora y fauna y llevarlo a cabo de manera permanente para tener inventarios actualizados	L
Monitorear los factores ambientales, sociales y económicos de mayor importancia y los sitios críticos presentes en el Anp	M
Difundir la información generada por las acciones de monitoreo	
Promover la participación de las y los usuarios de los recursos naturales en el monitoreo ambiental, socioeconómico y de la efectividad de manejo	P

*Las actividades se presentan en letra cursiva.

Componente de sistemas de información

Los Sistemas de Información Geográfica (SIG) son una herramienta que presenta información de la interacción de la dimensión ambiental, cultural, económica, social y espacial, entre otras, lo que permite realizar un análisis integral del territorio.

Es importante que toda la información generada y la obtenida de diversas fuentes sea organizada en bases de datos funcionales y permitan un acceso sencillo y eficiente.

Objetivo específico

- Contar con un sistema de información que concentre bases de datos ambientales, socioeconómicas e histórico-culturales del Área de Protección de Flora y Fauna Pico de Tancítaro que permita la proyección de posibles tendencias y condiciones del área, lo cual ayudará en la conservación, manejo y administración del Área Natural Protegida.

Meta y resultado esperado

- Contar con un sistema de información geográfica para la toma de decisiones del Anp a cinco años.

Actividades* y acciones	Plazo
Establecer un Sistema de Información Geográfica para el Área Natural Protegida	
Gestionar la adquisición del equipo necesario requerido	C
Generar la información base para el Sistema de Información Geográfica	M
Obtener imágenes de satélite y fotografía del Anp y su Zona de Influencia	C
Elaborar mapas de distribución de vegetación, fauna, edafología, ríos subterráneos, información social, económica, etc.	L
Actualizar periódicamente la información al SIG	P

*Las actividades se presentan en letra cursiva.

Subprograma de cultura

El acercamiento a un manejo sustentable del Área Natural Protegida obliga al conocimiento e interpretación integral de los procesos y fenómenos naturales, sociales y económicos que influyen en ella; sin embargo, no basta que los involucrados directamente en el manejo tengan una alta educación ambiental, sino que es necesario que la comunidad local y las y los turistas tengan el conocimiento básico que les permita reconocer la importancia de la preservación de los recursos naturales y su adecuado manejo.

Las comunidades colindantes son las que se benefician directamente con el establecimiento del Área Natural Protegida, por lo que deberán estar conscientes de la necesidad de cuidar,

proteger, preservar y conservar sus recursos. Para lograr esto es necesario que se ofrezca información a la población tanto dentro de los límites del Anp como de su Zona de Influencia.

Objetivo general

Difundir acciones de conservación del Área de Protección de Flora y Fauna Pico de Tancítaro, propiciando la valoración de los servicios ambientales mediante la difusión y la educación para la conservación de la biodiversidad que contiene.

Estrategias

- Fomentar proyectos de educación ambiental a nivel local, como una estrategia para minimizar los impactos que se generan por los principales problemas, como son la basura y la calidad del agua, así como los originados por la actividad turística.

- Conformar un esquema de participación multisectorial en los proyectos de educación ambiental, con la finalidad de fomentar la participación de todos los sectores sociales que tengan injerencia en el Anp.

- Diseñar y desarrollar materiales enfocados a la difusión, educación e interpretación ambiental.

Componente de fomento a la educación y cultura para la conservación

El fomento a la educación tiene como objetivo lograr que las y los prestadores de servicios, encargados y encargadas del cuidado del hogar, campesinos y campesinas, niños y niñas, jóvenes, adultos, autoridades, entre otros, tengan conocimiento sobre la importancia de conservar los recursos naturales presentes en el Área Natural Protegida y sobre todo que tengan una relación respetuosa y armónica con la naturaleza.

Objetivo específico

- Suscitar entre las y los habitantes de las comunidades que inciden en el Área de Protección de Flora y Fauna Pico de Tancítaro, las y los usuarios, prestadores de servicios, niños y niñas, adolescentes, adultos y público en general una actitud comprometida para conservar los recursos naturales presentes en el Área Natural Protegida.

Meta y resultado esperado

- Contar a tres años con una estrategia de educación ambiental para el Área Natural Protegida.

Actividades* y acciones	Plazo
Establecer una estrategia de educación ambiental enfocada a la conservación de los recursos naturales en el Área de Protección de Flora y Fauna Pico de Tancítaro	
Definir e implementar acciones de educación ambiental dirigidas al público en general sobre la importancia, conservación y uso sustentable de los recursos naturales presentes en el ANP	M
Establecer acuerdos de cooperación con instituciones y OSC en el desarrollo de actividades de educación ambiental	P
Integrar a la comunidad en las actividades de educación ambiental	P

*Las actividades se presentan en letra cursiva.

Componente de capacitación para el desarrollo sostenible

La base principal para la protección de los recursos naturales es una adecuada formación de las comunidades que hacen uso de ellos, creando conciencia de la importancia de su preservación y del establecimiento de las Áreas Naturales Protegidas. De la misma manera, se requiere capacitar y dar una formación adecuada a las comunidades, usuarios y usuarias acerca de los métodos básicos para interactuar con el medio ambiente circundante.

Este componente también contempla la capacitación del personal que labora en el Área Natural Protegida en búsqueda de una mejora en el desempeño de sus labores cotidianas.

Objetivo específico

- Fomentar entre las y los habitantes de las comunidades y prestadores (as) de servicios del Área Natural Protegida que adquieran habilidades y se formen o actualicen conocimientos para el desarrollo de los proyectos productivos sustentables con una actitud comprometida hacia la conservación de los recursos naturales.

Meta y resultado esperado

- Organizar al menos un taller anual de capacitación para las y los usuarios del ANP.

Actividades* y acciones	Plazo
Proporcionar capacitación y formación a comunidades y prestadores (as) de servicios	
Gestionar los recursos para el desarrollo de los talleres de capacitación	P
Crear un grupo de capacitadores (as) para difundir temas de prácticas productivas sostenibles	M
Impartir los talleres de capacitación en prácticas productivas sostenibles	P

*Las actividades se presentan en letra cursiva.

Componente de comunicación, difusión e interpretación ambiental

Este componente está encaminado a difundir y divulgar a la población local, regional, nacional e internacional información sobre la riqueza biológica del Anp, su importancia y el objetivo de su creación, buscando fomentar y promover su conservación, uso sustentable y entendimiento de los aspectos normativos aplicables en el Área. Todo ello con el fin de que las Anp dejen de percibirse como un obstáculo para el desarrollo y se consideren espacios donde se conservan y manejan bienes públicos que brindan beneficios a la sociedad y a las comunidades que forman parte directa o indirecta del Área de Protección de Flora y Fauna Pico de Tancítaro.

Objetivo específico

- Difundir las características y beneficios que brindan los recursos naturales del Área de Protección de Flora y Fauna Pico de Tancítaro entre las y los pobladores, usuarios y el ámbito interinstitucional a través de la participación en medios de comunicación impresos y medios masivos, y el acercamiento a las comunidades locales y la Zona de Influencia.

Meta y resultado esperado

- Contar a cinco años con un programa de difusión, comunicación e interpretación ambiental que transmita la información generada y que logre crear conciencia ambiental y un mejor conocimiento del Anp y su riqueza biológica, involucrando a las comunidades locales y al público en general.

Actividades* y acciones	Plazo
Diseñar e implementar un programa de difusión y divulgación	
Gestionar la adquisición de materiales gráficos impresos y audiovisuales sobre el Área Natural Protegida para que sean difundidos en los ámbitos local, estatal, nacional e internacional	P
Promover la participación de otras instancias para apoyar y consolidar el programa de comunicación y difusión del Área Natural Protegida	P
Gestionar y diseñar campañas de difusión para informar a la población local de la existencia, importancia y efectos negativos de las actividades humanas, así como la conservación de los recursos naturales presentes en el Área Natural Protegida	M
Diseñar y construir senderos interpretativos en el Área Natural Protegida	M

*Las actividades se presentan en letra cursiva.

Subprograma de gestión

La preservación y el manejo de la biodiversidad y los recursos naturales debe considerar la relación entre la dimensión social y ambiental en un espacio determinado, toda vez que el ser humano aprovecha los múltiples beneficios que le proporciona el medio ambiente, causando degradación en el mismo. Bajo este enfoque, es necesario que los diferentes actores sociales se involucren para respaldar las acciones de conservación y asegurar el financiamiento suficiente y oportuno para cubrir todos los aspectos funcionales y operativos durante la vigencia del Programa de Manejo y establecer acciones de coordinación y cooperación con OSC, institutos, universidades y los tres niveles de gobierno para fortalecer la gestión.

Por otra parte, y conforme a lo establecido en el Programa Nacional de Áreas Naturales Protegidas 2014-2018, la administración se lleva a cabo en estricto apego a un Programa Operativo Anual (POA), previamente autorizado por la Conanp, en concordancia con este Programa de Manejo y con la opinión de la sociedad a través de un Consejo Asesor del Área de Protección de Flora y Fauna Pico de Tancítaro.

Objetivo general

Establecer las formas en que se organizará la administración del Área de Protección de Flora y Fauna Pico de Tancítaro y los mecanismos de participación de los tres órdenes de gobierno, los individuos y las comunidades aledañas a la misma, así como de todas aquellas personas, instituciones, grupos y organizaciones sociales interesadas en su conservación y aprovechamiento sustentable.

Estrategias

- Contar con sistemas administrativos y operativos eficientes que garanticen el funcionamiento del Anp.

- Gestionar ante las diferentes instituciones locales, regionales y sectoriales convenios de colaboración y participación que enriquezcan los objetivos del Anp.

- Convenir con el sector privado la colaboración para el fomento y la promoción del Anp.

- Establecer comunicación con las diversas autoridades municipales, estatales y federales para la instalación de infraestructura, señalización y obra pública.

- Gestionar y dar seguimiento a la atención de los problemas legales del Área Natural Protegida y establecer la normatividad para su uso y manejo.

- Establecer el Consejo Asesor que involucre a todos los sectores de la población.

- Buscar la realización de convenios interinstitucionales nacionales e internacionales para lograr una mayor y mejor procuración de recursos.

- Contratar al personal con el mejor perfil profesional y/u operativo requerido.

- Establecer los mecanismos que permitan gestionar eficientemente el trámite de permisos, concesiones y autorizaciones.

Componente de administración y operación

Uno de los objetivos principales de la CONANP es la administración de calidad y la innovación de las Áreas Naturales Protegidas, por lo que la administración del Área de Protección de Flora y Fauna Pico de Tancítaro se llevará a cabo en apego a un Programa Operativo Anual, previamente autorizado por la CONANP y acorde con lo establecido en el presente Programa de Manejo y debidamente consensuado con el Consejo Asesor que se establezca para el ANP.

OBJETIVO ESPECÍFICO

- Lograr la adquisición, el manejo y el control de los recursos, tanto económicos como humanos, que garanticen la operación eficiente de los diversos componentes del Área Natural Protegida.

META Y RESULTADO ESPERADO

- Contar a cinco años con un presupuesto y recursos humanos que permitan cumplir con las actividades y acciones del Programa de Manejo y del Programa Operativo Anual (POA).

Actividades* y acciones	Plazo
Consolidación de la estructura administrativa	
Identificar las necesidades de personal para el logro de los objetivos del Área Natural Protegida	C
Consolidación del Consejo Asesor	
Impulsar la conformación del Consejo Asesor del ANP	M
Elaboración del Programa Operativo Anual (POA)	
Elaborar el POA con apoyo del Consejo Asesor y coordinado con la Dirección del Área	P
Presentar un informe anual de las actividades realizadas con base en lo planteado en el POA	P

*Las actividades se presentan en letra cursiva.

Componente de protección civil y mitigación de riesgos

Dentro del Área de Protección de Flora y Fauna Pico de Tancítaro inciden fenómenos físico-biológicos y actividades humanas que bajo ciertas condiciones pueden presentar factores de riesgo para el ecosistema y para las comunidades en la Zona de Influencia del área; por lo tanto, se requiere desarrollar las actividades necesarias para establecer los mecanismos adecuados que permitan minimizar los efectos negativos sobre los recursos naturales y las poblaciones cercanas.

Objetivo específico

- Incrementar la permanencia de los ecosistemas y seguridad de las y los usuarios y de las poblaciones aledañas al Anp ante los fenómenos catastróficos naturales o antropogénicos mediante la gestión de acuerdos con instituciones especializadas.

Meta y resultado esperado

- Contar a cinco años con un acuerdo de colaboración con otras dependencias para el manejo de contingencias ambientales y mitigación de riesgos.

Actividades* y acciones	Plazo
Establecer mecanismos de coordinación con otras dependencias para la atención de contingencias ambietales y mitigación de riesgos	
Coordinar con las autoridades competentes el establecimiento de acciones de atención ante contingencias ambientales	C
Implementar una campaña informativa sobre prevención de contingencias y mitigación de riesgos dentro del Área Natural Protegida	P

*Las actividades se presentan en letra cursiva.

Componente de infraestructura, señalización y obra pública

Este componente está enfocado en la implementación de la base física para garantizar la administración y operación del Área, mediante el cual se programa la adquisición de la infraestructura necesaria para poder llevar a cabo de forma eficiente los objetivos y las metas planteadas.

Es necesario contar con la infraestructura suficiente y necesaria para realizar la operación y el manejo del Área de Protección de Flora y Fauna Pico de Tancítaro, así como contar con la señalización pertinente que permita disminuir los impactos negativos sobre los recursos naturales y culturales y que informe puntualmente a las y los usuarios sobre la normatividad que rige en el área y el desarrollo de actividades permitidas conforme a la zonificación establecida, así como las facilidades y beneficios que el área les brinda.

Objetivo específico

- Contar con la infraestructura, la señalización y el equipamiento necesarios para el adecuado funcionamiento del Área Natural Protegida.

Meta y resultado esperado

- Contar con señalamientos restrictivos e informativos en los principales sitios de uso, acceso y caminos del Área.

Actividades* y acciones	Plazo
Diseñar una estrategia de señalización para el ANP	
Identificar los sitios potenciales para la ubicación de la señalización del Área Natural Protegida	M
Gestionar recursos para establecer la señalización requerida en el Área Natural Protegida	C
Colocar señalamientos de ubicación e información	M

*Las actividades se presentan en letra cursiva.

Componente de recursos humanos y profesionalización

El capital humano profesionalizado es un factor clave para la administración efectiva y estratégica del Área de Protección de Flora y Fauna Pico de Tancítaro, por lo que es necesario contar con personal preparado y capacitado para cumplir con los objetivos de conservación y manejo planteados en el presente Programa de Manejo.

Objetivo específico

- Contar con personal capacitado y suficiente para la operación y manejo del Área Natural Protegida.

Metas y resultados esperados

- Recibir al menos un curso de capacitación anual para el personal de la Dirección del Área Natural Protegida.
- Contar con el personal suficiente para cubrir las necesidades del ANP a cinco años.

Actividades* y acciones	Plazo
Formar la plantilla administrativa y operativa	
Gestionar la contratación de al menos tres personas para operar el área	M
Identificar las necesidades de capacitación del personal	
Gestionar la capacitación del personal a través de cursos, talleres, diplomados u otros procesos de profesionalización	P
Promover el intercambio de experiencias con otras Áreas Naturales Protegidas, Organizaciones de la Sociedad Civil y centros de investigación	P

*Las actividades se presentan en letra cursiva.

7. ORDENAMIENTO ECOLÓGICO Y ZONIFICACIÓN

Ordenamiento ecológico

Para la integración de esta sección se consideró el Programa de Ordenamiento Ecológico Regional de la Cuenca del Río Tepalcatepec, del estado de Michoacán de Ocampo, el cual fue publicado en el Periódico Oficial del Gobierno Constitucional del estado de Michoacán de Ocampo el 5 de junio de 2007; dicho Programa abarca los municipios de Tancítaro, Peribán de Ramos, Nuevo Parangaricutiro y Uruapan, que considera al Área de Protección de Flora y Fauna Pico de Tancítaro como una Unidad de Gestión Ambiental para todo el polígono; con bienes y servicios ambientales; donde los usos compatibles son el ecoturismo, el manejo sustentable de vida silvestre y la investigación; los usos condicionados son el forestal, la acuacultura, el doméstico y la sanidad; y como usos incompatibles se señalan los asentamientos humanos e industriales.

De conformidad con lo establecido en la fracción XXXVII del Artículo 3 de la Ley General del Equilibrio Ecológico y la Protección al Ambiente, la zonificación es el instrumento técnico de planeación que puede ser utilizado en el establecimiento de las Áreas Naturales Protegidas, que permite ordenar su territorio en función del grado de conservación y representatividad de sus ecosistemas, la vocación natural del terreno, de su uso actual y potencial, de conformidad con los objetivos dispuestos en la misma declaratoria. Asimismo, existirá una subzonificación, la cual consiste en el instrumento técnico y dinámico de planeación, que se establecerá en el programa respectivo, y que es utilizado en el manejo de las Áreas Naturales Protegidas.

Zonificación y subzonificación

Criterios de subzonificación

Para la subzonificación se consideró lo previsto en el Decreto publicado en el *Diario Oficial de la Federación* el 19 de

agosto de 2009, por el que se declara Área Natural Protegida, con el carácter de Área de Protección de Flora y Fauna, la región denominada Pico de Tancítaro, ubicada en los municipios de Tancítaro, Peribán de Ramos, Nuevo Parangaricutiro y Uruapan, en el estado de Michoacán y el marco definido por la Ley General del Equilibrio Ecológico y la Protección al Ambiente.

Además de lo anterior, para definir las subzonas que integran el Programa de Manejo se consideraron los siguientes criterios: 1) Según las características biológicas de cada subzona, tomando en cuenta la biodiversidad existente, como son especies presentes en la Norma Oficial Mexicana Nom-059-Semarnat-2010, Protección ambiental-Especies nativas de México de flora y fauna silvestres-Categorías de riesgo y especificaciones para su inclusión, exclusión o cambio-Lista de especies en riesgo; así como a los tipos de vegetación existentes; 2) De acuerdo a los servicios ambientales presentes, 3) De acuerdo al grado de conservación que presentan las subzonas y 4) Las actividades productivas.

El Decreto de Creación del Área Natural Protegida establece una superficie para el Área de Protección de Flora y Fauna Pico de Tancítaro de 23 mil 405-92-09.55 hectáreas; de esta superficie los tipos de vegetación son: bosque de pino-encino, (44.43 por ciento), bosque de pino (16.07 por ciento), bosque de pino abierto (11.22 por ciento), pastizal (4.62 por ciento), áreas sin vegetación aparente (2.16 por ciento) y cuerpos de agua (0.08 por ciento), además existen áreas agrícolas con el 21.41 por ciento y los asentamientos humanos con el 0.01 por ciento del total del área.

Metodología

Para la integración de la subzonificación del Anp se consideraron elementos de carácter geográfico, ecológico y de uso del suelo; además se tomó en cuenta la información contenida en los trabajos de investigación realizados en el polígono del Área Natural Protegida y su Zona de Influencia, que han llevado a cabo principalmente centros e institutos de investigación como es el Centro de Investigaciones en Ecosistemas (CIEco) y el Centro de Investigaciones en Geografía Ambiental (CIGA), ambos de la Universidad Nacional Autónoma de México.

Con la información antes mencionada se definió una propuesta de las subzonas, se editaron las bases de datos y se generó la cartografía correspondiente, usando las cartas digitales y los procesos de georreferenciación satelital; se integraron las coberturas de uso actual y potencial del suelo y vegetación en el área con base en el INEGI y la clasificación descrita por Rzedowski y a partir de las técnicas del Protocolo para la Evaluación del Uso del Suelo y Vegetación en Áreas Naturales Protegidas Federales de México (Conanp, 2007).

Subzonas y políticas de manejo

La subzonificación para el Área de Protección de Flora y Fauna es la siguiente:

I. **Zona Núcleo.** Conformadas por las subzonas:

   a) **De Protección Pico de Tancítaro-Piedra del Horno,** con una extensión de mil 080.563357 hectáreas, conformada por un polígono, y

   b) **De Uso Restringido Cerro Tancítaro-La Culebra y La Zafra-Cerro San Pedro,** con una superficie de mil 709.738968 hectáreas, está integrada por dos polígonos.

II. **Zona de Amortiguamiento.** Conformadas por las subzonas:

   a) **De Aprovechamiento Sustentable de los Recursos Naturales Laderas Medias Circundantes del Tancítaro,** abarca una superficie de 12 mil 286.690369 hectáreas, constituida por cuatro polígonos;

   b) **De Aprovechamiento Sustentable de los Ecosistemas Laderas Bajas del Tancítaro,** abarca una superficie de 6 mil 889.181620 hectáreas, comprendida en siete polígonos;

   c) **De Uso Público Cerro Grande-Cenizas Volcánicas,** abarca una superficie de 730.600124 hectáreas, constituida por tres polígonos; y,

   d) **De Recuperación Ladera Norte y Ladera Oriente,** abarca una superficie de 709.146517 hectáreas, constituida por dos polígonos.

Zona Núcleo

Subzona de Protección Pico de Tancítaro-Piedra del Horno.

Esta subzona la conforma un polígono cuyo nombre es Pico de Tancítaro-Piedra del Horno, ubicado en la parte central del Área Natural Protegida, con una extensión de mil 080.563357 hectáreas. Se consideró por su importancia en términos del recurso hídrico al ser una zona de captación y generación de manantiales permanentes, lo que la convierte en una subzona de importancia hidrológica al suministrar este recurso a diversas comunidades, tanto dentro como fuera del Área Natural Protegida. Esto es posible dado el grado de conservación de los macizos forestales que ahí se encuentran con especies como el pino *(Pinus hartwegii)* y el oyamel *(Abies religiosa).* Además existen paisajes naturales singulares, como el conocido Piedra del Horno y el propio volcán Pico de Tancítaro, que le da nombre al Área Natural Protegida.

En cuanto a la vegetación, la especie enebro azul *(Juniperus monticola)* se encuentra presente en esta subzona, la cual es de importancia por presentar categoría de protección especial de acuerdo a la Norma Oficial Mexicana Nom-059-Semarnat-2010, Protección ambiental-Especies nativas de México de flora y fauna silvestres-Categorías de riesgo y especificaciones para su inclusión, exclusión o cambio-Lista de especies en riesgo. Asimismo, se encuentran macizos

de pino *(Pinus hartwegii)*, un tipo de vegetación de distribución restringida a las zonas altas, así como el zacatonal o páramo de altura, el cual se compone comúnmente de algunas especies de gramíneas, tales como zacate barbón *(Aegopogon cenchroides)*, *Bouteloua purpurea*, zacate, pastos *(Festuca amplissima* y *Festuca breviglumis*), zacate *(Muhlenbergia macroura)*, zacate azul *(Poa annua)*, zacate *(Trisetum virlettii)* y *Zeugites americanus*.

En cuanto a la fauna, es posible encontrar las siguientes especies de anfibios endémicos y con alguna categoría de riesgo dentro de la Norma Oficial Mexicana Nom-059-Semarnat-2010, Protección ambiental-Especies nativas de México de flora y fauna silvestres-Categorías de riesgo y especificaciones para su inclusión, exclusión o cambio-Lista de especies en riesgo: en la categoría de sujeta a protección especial están la rana fisgona de Pátzcuaro *(Eleutherodactylus angustidigitorum)*, la rana de Moctezuma *(Lithobates montezumae)* y el tlaconete pinto *(Pseudoeurycea belli)*, especie endémica y amenazada.

En cuanto a los reptiles, sobresale la presencia de la culebra terrestre dos líneas *(Conopsis biserialis)* y la culebra sorda mexicana *(Pituophis deppei)*, las cuales son especies de culebras endémicas y con categoría de amenazadas según la Norma Oficial Mexicana Nom-059-Semarnat-2010, Protección ambiental-Especies nativas de México de flora y fauna silvestres-Categorías de riesgo y especificaciones para su inclusión, exclusión o cambio-Lista de especies en riesgo, en la categoría de sujeta a protección especial; también endémicas pero con categoría de protección especial se encuentran el eslizón de copete *(Plestiodon copei)* y el eslizón de Duges *(Plestiodon dugesi)*.

Con relación a los mamíferos, se encuentran especies del orden Rodentia, enlistadas en la Norma Oficial Mexicana Nom-059-Semarnat-2010, Protección ambiental-Especies nativas de México de flora y fauna silvestres-Categorías de riesgo y especificaciones para su inclusión, exclusión o cambio-Lista de especies en riesgo en la categoría de sujetas a protección especial, aunque con escasa distribución dentro de la subzona.

Finalmente, se tienen registros del cacomixtle *(Bassariscus astutus)* y zorra gris *(Urocyon cinereoargenteus)*; estas especies, aunque no están en alguna categoría de riesgo, sí son interesantes, por estar registradas por encima de los tres mil 500 metros sobre el nivel del mar.

Las actividades permitidas y no permitidas en esta subzona se establecen en el siguiente cuadro:

Subzona de Protección Pico de Tancítaro-Piedra del Horno	
**Actividades Permitidas**	**Actividades no Permitidas**
1. Investigación científica y monitoreo ambiental, sin extracción de flora o fauna	1. Abrir senderos, brechas o caminos<br>2. Agricultura<br>3. Alimentar, tocar o hacer ruidos intensos que alteren el comportamiento natural de los ejemplares de la vida silvestre<br>4. Alterar o destruir por cualquier medio o acción los sitios de alimentación, anidación, refugio o reproducción de las especies silvestres<br>5. Aprovechamiento forestal<br>6. Apertura de bancos de materiales<br>7. Arrojar, verter o descargar cualquier tipo de desechos orgánicos, residuos sólidos o líquidos o cualquier otro tipo de contaminante, tales como insecticidas, fungicidas y pesticidas, entre otros, al suelo o a cuerpos de agua<br>8. Cambiar el uso de suelo<br>9. Colecta científica<br>10. Construcción de obra pública o privada<br>11. Dañar, cortar y marcar árboles<br>12. Encender fogatas<br>13. Establecimiento de Unidades de Manejo para la Conservación de Vida Silvestre<br>14. Ganadería<br>15. Hacer uso de explosivos<br>16. Interrumpir, rellenar, desecar o desviar flujos hidráulicos<br>17. Introducir ejemplares o poblaciones silvestres exóticas[1]<br>18. Introducir organismos genéticamente modificados<br>19. Introducir plantas, semillas y animales domésticos<br>20. Modificar cuencas hidrológicas, cauces naturales, manantiales<br>21. Molestar, capturar, remover, extraer, retener o apropiarse de vida silvestre o sus productos<br>22. Motociclismo extremo (enduro y cuatrimotor)

Subzona de Protección Pico de Tancítaro-Piedra del Horno	
**Actividades Permitidas**	**Actividades no Permitidas**
	23. Realizar actividades cinegenéticas, o de explotación y aprovechamiento de especies de flora y fauna silvestres y extracción de tierra de monte y su cubierta vegetal<br>24. Remover o extraer material mineral<br>25. Trasladar especímenes de poblaciones nativas de una comunidad biológica a otra<br>26. Turismo y turismo de bajo impacto ambiental<br>27. Usar altavoces, radios o cualquier aparato de sonido que altere el comportamiento de las poblaciones o ejemplares de las especies silvestres<br>28. Utilizar lámparas o cualquier fuente de luz para aprovechamiento u observación de ejemplares de la vida silvestre, salvo para actividades de investigación científica y monitoreo científico<br>29. Verter o descargar contaminantes en el suelo, subsuelo y cualquier clase de cause, vaso o acuífero, así como desarrollar cualquier actividad contaminante

[1] Conforme a lo establecido en las fracciones XIII y XVII del Artículo 3o., de la Ley General de Vida Silvestre.

Subzona de Uso Restringido Cerro Tancítaro-La Culebra y La Zafra-Cerro San Pedro

Esta subzona está integrada por dos polígonos, con una superficie total de mil 709.738968 hectáreas. El polígono 1 La Zafra-Cerro San Pedro, con una superficie de 835.898990 hectáreas, y El polígono 2 Cerro Tancítaro-La Culebra, con una superficie de 873.839978, están ubicados en la parte central del Área Natural Protegida. En esta subzona se encuentran fragmentos de bosques de pino-encino, de pino abierto, bosques mixtos de abeto-encino-pino, pastizal y áreas sin vegetación aparente (zonas de cenizas volcánicas), además existe la presencia de bosques de oyamel *(Abies religiosa),* los cuales forman masas continuas bien conservadas, en cañadas por arriba de los dos mil 800 metros sobre el nivel del mar, las cuales favorecen la formación de escurrimientos perennes e intermitentes para la captación de agua. Dentro de estas subzonas se localiza el área de influencia del nacimiento de dos manantiales de gran importancia para el abasto de agua potable de poblaciones como Zacán, en la ladera norte de la subzona La Zafra-Cerro San Pedro y Tancítaro y Apo, en la ladera poniente.

Por su ubicación presenta una mayor distribución efectiva de especies enlistadas en la Norma Oficial Mexicana Nom-059-Semarnat-2010, Protección ambiental-Especies nativas de México de flora y fauna silvestres-Categorías de riesgo y especificaciones para su inclusión, exclusión o cambio-Lista de especies en riesgo; dentro de las especies de flora podemos encontrar la vara de San Juan *(Phymosia rosea),* el enebro azul *(Juniperus monticola)* y el cedro blanco *(Cupressus lusitánica),* especies sujetas a Protección Especial; así como la flor de tila *(Tilia mexicana),* la cual se encuentra en peligro de extinción. Asimismo, se encuentran especies de fauna, como el tlaconete pinto *(Pseudoeurycea belli),* el murciélago hocicudo *(Leptonycteris curasoae)* y el murciélago trompudo *(Choeronycteris mexicana),* especies en categoría de Amenazadas. Por otra parte, también se encuentra el ratón *(Peromyscus difficilis),* la cual es una especie endémica de la región.

Las actividades permitidas y no permitidas en esta subzona se establecen en el siguiente cuadro :

**Subzona de Uso Restringido Cerro de Tancítaro-La Culebra y La Zafra-Cerro San Pedro**	
**Actividades permitidas**	**Actividades no permitidas**
1. Colecta científica[1]<br>2. Colecta cientifica[2]<br>3. Educación ambiental que no implique ninguna actividad extractiva<br>4. Filmaciones, fotografías, captura de imágenes y sonidos por cualquier medio, con fines científicos y culturales<br>5. Investigación científica y monitoreo ambiental<br>6. Turismo de bajo impacto ambiental	1. Abrir senderos, brechas o caminos<br>2. Agricultura<br>3. Alimentar, tocar o hacer ruidos intensos que alteren el comportamiento natural de los ejemplares de la vida silvestre<br>4. Alterar o destruir por cualquier medio o acción los sitios de alimentación, anidación, refugio o reproducción de las especies silvestres<br>5. Aprovechamiento forestal<br>6. Apertura de bancos de materiales<br>7. Arrojar, verter o descargar cualquier tipo de desechos orgánicos, residuos sólidos o líquidos o cualquier otro tipo de contaminante, tales como insecticidas, fungicidas y pesticidas, entre otros, al suelo o a cuerpos de agua<br>8. Cambiar el uso de suelo<br>9. Dañar, cortar y marcar árboles<br>10. Encender fogatas<br>11. Establecimiento de Unidades de Manejo para la Conservación de Vida Silvestre<br>12. Ganadería<br>13. Hacer uso de explosivos<br>14. Interrumpir, rellenar, desecar o desviar flujos hidráulicos<br>15. Introducir ejemplares o poblaciones silvestres exóticas[3]<br>16. Introducir organismos genéticamente modificados<br>17. Introducir plantas, semillas y animales domésticos<br>18. Modificar cuencas hidrológicas, cauces naturales, manantiales<br>19. Molestar, capturar, remover, extraer, retener o apropiarse de vida silvestre o sus productos, salvo para actividades de investigación científica<br>20. Motociclismo extremo (enduro y cuatrimotor)

Subzona de Uso Restringido Cerro de Tancítaro-La Culebra y La Zafra-Cerro San Pedro	
**Actividades permitidas**	**Actividades no permitidas**
	21. Realizar actividades cinegenéticas o de explotación y aprovechamiento de especies de flora y fauna silvestres y extracción de tierra de monte y su cubierta vegetal<br>22. Remover o extraer material mineral<br>23. Trasladar especímenes de poblaciones nativas de una comunidad biológica a otra<br>24. Usar altavoces, radios o cualquier aparato de sonido que altere el comportamiento de las poblaciones o ejemplares de las especies silvestres<br>25. Utilizar lámparas o cualquier fuente de luz para aprovechamiento u observación de ejemplares de la vida silvestre, salvo para actividades de investigación científica y monitoreo científico<br>26. Verter o descargar contaminantes en el suelo, subsuelo y cualquier clase de cause, vaso o acuífero, así como desarrollar cualquier actividad contaminante

[1] Conforme a lo previsto por el Artículo 2o., fracción VI, del Reglamento de la Ley General de Vida Silvestre.
[2] Conforme a lo previsto por el Artículo 2o., fracción VII, del Reglamento de la Ley General de Desarrollo Forestal Sustentable.
[3] Conforme a lo establecido en las fracciones XIII y XVII del Artículo 3o., de la Ley General de Vida Silvestre.

Zona de Amortiguamiento

Subzona de Aprovechamiento Sustentable de los Recursos Naturales Laderas Medias Circundantes del Tancítaro

Abarca una superficie de 12 mil 286.690369 hectáreas, constituida por cuatro polígonos: El polígono 1 Laderas Medias Circundantes al Pico de Tancítaro, con una superficie de 11 mil 534.946440 hectáreas, se encuentra ubicado en las laderas de las partes más altas del Área Natural Protegida; El polígono 2 Cerro Prieto, con una superficie de 484.100378 hectáreas, ubicado al este del Área Natural Protegida; El polígono 3 Barranca Pedregosa, con una superficie de 93.291708 hectáreas, se encuentra ubicado en la parte oeste y El polígono 4 Cerro La Chimenea, con una superficie de 174.351843 hectáreas, también ubicado en la parte este del Área de Protección de Flora y Fauna Pico de Tancítaro.

Esta subzona contiene superficies con altitud y pendiente considerables ubicadas en laderas medias que rodean al Pico de Tancítaro, que contiene vegetación de bosque de pino-encino, bosque de pino, bosque de pino abierto,

pastizal y un área sin vegetación aparente por las cenizas volcánicas.

En esta subzona se conservan en su mayoría los servicios ambientales mencionados para las dos anteriores: captura de carbono, regulación climática, servicios hidrológicos, recreación y belleza escénica entre otros, así como el uso y aprovechamiento sustentable de los recursos naturales.

El Área Natural Protegida alberga diferentes especies en alguna categoría de riesgo de acuerdo a la Norma Oficial Mexicana Nom-059-Semarnat-2010, Protección ambiental-Especies nativas de México de flora y fauna silvestres-Categorías de riesgo y especificaciones para su inclusión, exclusión o cambio-Lista de especies en riesgo, incluyendo especies vegetales relevantes, como la flor de tila *(Tilia mexicana)*, especie en peligro de extinción; la vara de San Juan *(Phymosia rosea)*, el enebro azul *(Juniperus monticola)* y el cedro blanco *(Cupressus lusitanica)*, especies sujetas a protección especial. Asimismo, alberga especies de fauna enlistadas en la misma Norma Oficial Mexicana, como anfibios y reptiles, como el tlaconete pinto *(Pseudoeurycea belli)*, la culebra terrestre dos líneas *(Conopsis biserialis)*, la víbora de cascabel *(Crotalus pusillus)* y la culebra listonada cuello negro *(Thamnophis cyrtopsis)*, especies amenazadas; la rana fisgona de Pátzcuaro *(Eleutherodactylus angustidigitorum)*, la rana de Moctezuma *(Lithobates montezumae)*, la tortuga casquito *(Kinosternon integrum)*, el eslizón de Cope *(Plestiodon copei)*, *el* eslizón de Duges *(Plestiodon duges)*, la culebra parchada de Baird *(Salvadora bairdi)*, la rana de árbol de pliegue mexicana *(Plectrohyla bistincta)* y la lagartija escamosa de mezquite *(Sceloporus grammicus)*, especies sujetas a protección especial; así como mamíferos: murciélago hocicudo de Curazao *(Leptonycteris curasoae)* y murciélago trompudo *(Choeronycteris mexicana)*, con categoría de amenazadas y tuza michoacana *(Zygogeomys trichopus)*, en peligro de extinción.

Asimismo, esta subzona constituye el hábitat de especies como el venado cola blanca *(Odocoileus virginianus)*, el gato de monte *(Lynx rufus)*, la zorra gris *(Urocyon cinereoargenteus)*, el lagarto alicante *(Barisia imbricata)*, el aguilillla cola roja *(Buteo jamaicensis)*, el mulato *(Melanotis caerulescens)*, los colibríes y la rata de campo, entre otros.

Las actividades permitidas y no permitidas en esta subzona se establecen en el siguiente cuadro:

Subzona de Aprovechamiento Sustentable de los Recursos Naturales Laderas Medias Circundantes del Tancítaro	
**Actividades permitidas**	**Actividades no permitidas**
1. Apertura de senderos interpretativos<br>2. Aprovechamiento forestal<br>3. Colecta científica[1]<br>4. Colecta científica[2]<br>5. Construcción exclusivamente de instalaciones de apoyo para la investigación y monitoreo del ambiente[3]<br>6. Educación ambiental<br>7. Aprovechamiento extractivo de vida silvestre bajo el esquema de UMA[4]<br>8. Filmaciones, fotografías, captura de imágenes y sonidos por cualquier medio<br>9. Turismo de bajo impacto ambiental	1. Abrir brechas o caminos, salvo senderos interpretativos<br>2. Agricultura<br>3. Alimentar, tocar o hacer ruidos intensos que alteren el comportamiento natural de los ejemplares de la vida silvestre<br>4. Alterar o destruir por cualquier medio o acción los sitios de alimentación, anidación, refugio o reproducción de las especies silvestres<br>5. Aprovechar bancos de materiales<br>6. Arrojar, verter o descargar cualquier tipo de desechos orgánicos, residuos sólidos o líquidos o cualquier otro tipo de contaminante, tales como insecticidas, fungicidas y pesticidas, entre otros, al suelo o a cuerpos de agua<br>7. Cambiar el uso de suelo, salvo para la construcción de instalaciones de apoyo para la investigación y monitoreo del ambiente<br>8. Construcción de obra pública o privada, salvo de instalaciones de apoyo para la investigación y monitoreo del ambiente<br>9. Construir confinamientos para materiales y sustancias peligrosas<br>10. Encender fogatas<br>11. Ganadería<br>12. Hacer uso de explosivos<br>13. Introducir ejemplares o poblaciones silvestres exóticas[5]<br>14. Introducir plantas, semillas y animales domésticos<br>15. Modificar cuencas hidrológicas, cauces naturales o manantiales<br>16. Molestar, capturar, remover, extraer, retener o apropiarse de vida silvestre o sus productos, salvo para actividades de investigación y colecta científica que así lo requieran

Subzona de Aprovechamiento Sustentable de los Recursos Naturales Laderas Medias Circundantes del Tancítaro	
**Actividades permitidas**	**Actividades no permitidas**
	17. Remover o extraer material mineral<br>18. Tirar o abandonar residuos<br>19. Trasladar especímenes de poblaciones nativas de una comunidad biológica a otra<br>20. Turismo<br>21. Usar altavoces, radios o cualquier aparato de sonido que altere el comportamiento de las poblaciones o ejemplares de las especies silvestres<br>22. Utilizar lámparas o cualquier fuente de luz para aprovechamiento u observación de ejemplares de la vida silvestre, salvo para actividades de investigación y colecta científica que así lo requieran<br>23. Verter o descartar desechos o cualquier tipo de material nocivo en el suelo, subsuelo y en cualquier clase de cauce, vaso o acuífero

[1] Conforme a lo previsto por el Artículo 2o., fracción VI, del Reglamento de la Ley General de Vida Silvestre.

[2] Conforme a lo previsto por el Artículo 2o., fracción VII, del Reglamento de la Ley General de Desarrollo Forestal Sustentable.

[3] Utilizando ecotecnias y materiales tradicionales de construcción propios de la región y sin cercados que obstaculicen el cruce de fauna silvestre.

[4] Siempre y cuando se garantice la preservación de las especies aprovechadas, conforme a las disposiciones legales y reglamentarias aplicables.

[5] Conforme a lo establecido en las fracciones XIII y XVII del Artículo 3o., de la Ley General de Vida Silvestre.

Subzona de Aprovechamiento Sustentable de los Ecosistemas Laderas Bajas del Tancítaro

Esta subzona abarca una superficie de seis mil 889.181620 hectáreas, comprendida en siete polígonos, ubicados en las partes bajas y los límites del Área Natural Protegida: El polígono 1 Uña de Gato-La Majada, con una superficie de mil 726.405325 hectáreas; El polígono 2 Barranca La Angostura, con una superficie de 206.598247 hectáreas; El polígono 3 El Jazmín-Plan de Parastaco, con una superficie de mil 746.999141 hectáreas; El polígono 4 La Escondida, con una superficie de 434.503584 hectáreas; El polígono 5 Barranca Bueyera con una superficie de 166.742259; El polígono 6 Tiscato, con una superficie de mil 376.758575 hectáreas, y El polígono 7 Zirimóndiro, con una superficie de mil 231.174489 hectáreas.

En esta subzona predominan áreas agrícolas, entre las cuales existen manchones aislados de bosque de pino-encino, bosque de pino y bosque de pino abierto, con individuos de pinos *(Pinus pseudostrobus, Pinus leiophylla, Pinus hartwegii, Pinus douglasiana* y *Pinus montezumae),* encinos *(Quercus rugosa, Quercus martinezii, Quercus laurina, Quercus crassifolia, Quercus obtusata),* aile *(Alnus jorullensis* subsp. *lutea),* zacate *(Muhlenbergia macroura)* y pericón *(Tagetes filifolia),* así como enebro azul *(Juniperus monticola)* y cedro blanco *(Cupressus lusitanica).*

Esta superficie requiere altos volúmenes de agua provenientes de numerosos arroyos permanentes e intermitentes, los cuales se encuentran principalmente aprovechados para el riego de huertas de aguacate.

Las actividades permitidas y no permitidas en esta subzona se establecen en el siguiente cuadro:

Subzona de aprovechamiento sustentable de los ecosistemas Laderas Bajas del Tancítaro	
**Actividades permitidas**	**Actividades no permitidas**
1. Agricultura[1]<br>2. Apertura de senderos interpretativos<br>3. Aprovechamiento forestal<br>4. Colecta científica[2]<br>5. Colecta científica[3]<br>6. Construcción de infraestructura de apoyo a las actividades de investigación científica y educación ambiental[4]<br>7. Educación ambiental<br>8. Aprovechamiento extractivo de vida silvestre bajo el esquema de UMA[5]<br>9. Filmaciones, fotografías, captura de imágenes y sonidos por cualquier medio<br>10. Ganadería[6]<br>11. Investigación científica y monitoreo ambiental<br>12. Mantenimiento de caminos existentes<br>13. Turismo de bajo impacto ambiental	1. Abrir brechas o caminos, salvo senderos interpretativos<br>2. Alimentar, tocar o hacer ruidos intensos que alteren el comportamiento natural de los ejemplares de la vida silvestre<br>3. Alterar o destruir por cualquier medio o acción los sitios de alimentación, anidación, refugio o reproducción de las especies silvestres<br>4. Arrojar, verter o descargar cualquier tipo de desechos orgánicos, residuos sólidos o líquidos o cualquier otro tipo de contaminante, tales como insecticidas, fungicidas y pesticidas, entre otros, al suelo o a cuerpos de agua<br>5. Construcción de obra pública o privada, salvo infraestructura de apoyo a las actividades de investigación científica y educación ambiental<br>6. Encender fogatas<br>7. Hacer uso de explosivos<br>8. Interrumpir, rellenar, desecar o desviar flujos hidráulicos<br>9. Introducir ejemplares o poblaciones silvestres exóticas[7]<br>10. Molestar, capturar, remover, extraer, retener o apropiarse de vida silvestre o sus productos, salvo para actividades de investigación y colecta científica que así lo requieran<br>11. Aprovechar bancos de materiales<br>12. Trasladar especímenes de poblaciones nativas de una comunidad biológica a otra<br>13. Usar altavoces, radios o cualquier aparato de sonido que altere el comportamiento de las poblaciones o ejemplares de las especies silvestres

Subzona de aprovechamiento sustentable de los ecosistemas Laderas Bajas del Tancítaro	
**Actividades permitidas**	**Actividades no permitidas**
	14. Utilizar lámparas o cualquier fuente de luz para aprovechamiento u observación de ejemplares de la vida silvestre, salvo para actividades de investigación y colecta científica que así lo requieran<br>15. Verter o descartar, desechos o cualquier tipo de material nocivo en el suelo, subsuelo y en cualquier clase de cauce, vaso o acuífero

[1 y 6] Siempre y cuando sean de baja intensidad y se lleven a cabo en predios que cuenten con aptitud para este fin y en aquellos en que dichas actividades se realicen de manera cotidiana; asimismo, se podrán realizar actividades de agroforestería y silvopastoriles, siempre y cuando sean compatibles con las acciones de conservación del área y que contribuyan al control de la erosión y evitar la degradación de los suelos.
[2] Conforme a lo previsto por el Artículo 2o., fracción VI, del Reglamento de la Ley General de Vida Silvestre.
[3] Conforme a lo previsto por el Artículo 2o., fracción VII, del Reglamento de la Ley General de Desarrollo Forestal Sustentable.
[4] Utilizando ecotecnias y materiales tradicionales de construcción propios de la región y sin cercados que obstaculicen el cruce de fauna silvestre.
[5] Siempre y cuando se garantice la preservación de las especies aprovechadas, conforme a las disposiciones legales y reglamentarias aplicables.
[7] Conforme a lo establecido en las fracciones XIII y XVII del Artículo 3o, de la Ley General de Vida Silvestre.

Subzona de Uso Público Cerro Grande-Cenizas Volcánicas

Esta subzona abarca una superficie de 730.600124 hectáreas, constituida por tres polígonos: El polígono 1 Llanos de Cenizas del Oeste del Paricutín, con una superficie de 118.955223 hectáreas; El polígono 2 Llanos de Cenizas del Suroeste del Paricutín, con una superficie de 322.771755 hectáreas, ubicados al noreste del polígono del Área Natural Protegida; y El polígono 3 La Zafra-Paso La Nieve, con una superficie de 288.873146 hectáreas, ubicado en la parte centro-oeste del Área Natural Protegida, en el cual existen bosques de pino y pino-encino, así como laderas con belleza escénica. Los polígonos Llanos de Cenizas están conformados por áreas sin vegetación aparente y áreas de rocas ígneas derivadas de erupciones recientes del Volcán Paricutín, con manchones de bosque de pino abierto y bosque de pino-encino, así como áreas de pastizal.

Esta subzona es de especial importancia hídrica para la recarga de acuíferos debido al tipo de sustrato geológico, el cual suele ser muy poroso. Además, estos sitios poseen vistas singulares de interés para la recreación, el disfrute y el esparcimiento.

Las actividades permitidas y no permitidas en esta subzona se establecen en el siguiente cuadro:

Subzona de Uso Público Cerro Grande-Cenizas Volcánicas	
**Actividades permitidas**	**Actividades no permitidas**
1. Apertura de senderos interpretativos<br>2. Colecta científica[1]<br>3. Colecta científica[2]<br>4. Construcción y mantenimiento de infraestructura de apoyo al turismo, a la investigación científica, el monitoreo del ambiente y la educación ambiental[3]<br>5. Educación ambiental<br>6. Aprovechamiento extractivo de vida silvestre bajo el esquema de UMA [4]<br>7. Filmaciones, fotografías, captura de imágenes y sonidos por cualquier medio<br>8. Investigación científica y monitoreo del ambiente<br>9. Turismo y turismo de bajo impacto ambiental<br>10. Encender fogatas[5]<br>11. Señalización con fines de administración y manejo<br>12. Venta de alimentos y artesanías	1. Abrir brechas o caminos, salvo senderos interpretativos<br>2. Alimentar, tocar o hacer ruidos intensos que alteren el comportamiento natural de los ejemplares de la vida silvestre<br>3. Agricultura<br>4. Alterar o destruir por cualquier medio o acción los sitios de alimentación, anidación, refugio o reproducción de las especies silvestres<br>5. Aprovechamiento forestal<br>6. Arrojar, verter o descargar cualquier tipo de desechos orgánicos, residuos sólidos o líquidos o cualquier otro tipo de contaminante, tales como insecticidas, fungicidas y pesticidas, entre otros, al suelo o a los cuerpos de agua<br>7. Cambiar el uso de suelo, salvo para la infraestructura de apoyo al turismo, a la investigación científica, el monitoreo del ambiente y la educación ambiental<br>8. Construcción de obra pública o privada, salvo para la infraestructura de apoyo al turismo, a la investigación científica, al monitoreo del ambiente y la educación ambiental<br>9. Construir confinamientos de materiales y sustancias peligrosas y no peligrosas<br>10. Ganadería<br>11. Hacer uso de explosivos<br>12. Interrumpir, rellenar, desecar o desviar flujos hidráulicos<br>13. Introducir ejemplares o poblaciones silvestres exóticas[6]<br>14. Introducir organismos genéticamente modificados<br>15. Modificar cuencas hidrológicas, cauces naturales y manantiales<br>16. Molestar, capturar, remover, extraer, retener o apropiarse de vida silvestre o sus productos, salvo para actividades de investigación y colecta científica que así lo requieran

Subzona de Uso Público Cerro Grande-Cenizas Volcánicas	
**Actividades permitidas**	**Actividades no permitidas**
	17. Realizar actividades cinegéticas, o de explotación y aprovechamiento de especies de flora y fauna silvestres y extracción de tierra de monte y su cubierta vegetal<br>18. Aprovechar bancos de materiales<br>19. Usar altavoces, radios o cualquier aparato de sonido, que altere el comportamiento de las poblaciones o ejemplares de las especies silvestres<br>20. Utilizar lámparas o cualquier fuente de luz para aprovechamiento u observación de ejemplares de la vida silvestre, salvo para actividades de investigación científica y monitoreo científico que así lo requieran<br>21. Verter o descartar, desechos o cualquier tipo de material nocivo en el suelo, subsuelo y en cualquier clase de cauce, vaso o acuífero

[1] Conforme a lo previsto por el Artículo 2o., fracción VI, del Reglamento de la Ley General de Vida Silvestre.
[2] Conforme a lo previsto por el Artículo 2o., fracción VII, del Reglamento de la Ley General de Desarrollo Forestal Sustentable.
[3] Utilizando ecotecnias y materiales tradiciones de construcción propios de la región y sin cercados que obstaculicen el cruce de fauna silvestre.
[4] Exclusivamente de aprovechamiento no extractivo, siempre y cuando se garantice la preservación de las especies aprovechadas, conforme a las disposiciones legales y reglamentarias aplicables.
[5] Unicamente en los sitios destinados para ello.
[6] Conforme a lo establecido en las fracciones XIII y XVII del Artículo 3o, de la Ley General de Vida Silvestre.

Subzona de Recuperación Ladera Norte y Ladera Oriente

Esta subzona abarca una superficie de 709.146517 hectáreas, constituida por dos polígonos: El polígono 1 Mesa Isingo-La Caja, con una superficie de 303.430166 hectáreas, se encuentra ubicado en la parte norte del polígono de la Subzona de Uso Restringido; y El polígono 2 La Culebra-El Tepetate, con una superficie de 405.716351 hectáreas, ubicado al este de la Subzona de Protección Pico de Tancítaro-Piedra del Horno. Comprende superficies que presentan degradación alta, debido a la erosión por la deforestación y en algunos casos al uso intensivo del territorio.

Las actividades permitidas y no permitidas en esta subzona se establecen en el siguiente cuadro:

Subzona de Recuperación Ladera Norte y Ladera Oriente	
**Actividades permitidas**	**Actividades no permitidas**
1. Investigación científica y monitoreo ambiental<br>2.Colecta científica[1]<br>3. Colecta científica[2]<br>4. Educación ambiental<br>5. Obras de conservación y recuperación de suelos[3]	1. Alimentar, tocar o hacer ruidos intensos que alteren el comportamiento natural de los ejemplares de la vida silvestre<br>2. Agricultura<br>3. Alterar o destruir por cualquier medio o acción los sitios de alimentación, anidación, refugio o reproducción de las especies silvestres<br>4. Abrir senderos, brechas o caminos<br>5. Aprovechamiento forestal<br>6. Arrojar, verter o descargar cualquier tipo de desechos orgánicos, residuos sólidos o líquidos o cualquier otro tipo de contaminante, tales como insecticidas, fungicidas y pesticidas, entre otros, al suelo o a los cuerpos de agua<br>7. Cambiar el uso de suelo, salvo para la infraestructura de apoyo al turismo, a la investigación científica, el monitoreo del ambiente y la educación ambiental<br>8. Construcción de obra pública o privada, salvo para la infraestructura de apoyo al turismo, a la investigación científica, al monitoreo del ambiente y la educación ambiental<br>9. Dañar, cortar y marcar árboles<br>10. Encender fogatas<br>11. Establecimiento de UMA<br>12. Ganadería<br>13. Hacer uso de explosivos<br>14. Interrumpir, rellenar, desecar o desviar flujos hidráulicos<br>15. Introducir ejemplares o poblaciones silvestres exóticas[4]<br>16. Introducir organismos genéticamente modificados<br>17. Introducir plantas, semillas y animales domésticos<br>18. Modificar cuencas hidrológicas, cauces naturales, manantiales

Subzona de Recuperación Ladera Norte y Ladera Oriente	
**Actividades permitidas**	**Actividades no permitidas**
	19. Molestar, capturar, remover, extraer, retener o apropiarse de vida silvestre o sus productos<br>20. Realizar actividades cinegéticas o de explotación y aprovechamiento de especies de flora y fauna silvestres y extracción de tierra de monte y su cubierta vegetal<br>21. Apertura de bancos de materiales<br>22. Trasladar especímenes de poblaciones nativas de una comunidad biológica a otra<br>23. Turismo y turismo de bajo impacto ambiental<br>19. Usar altavoces, radios o cualquier aparato de sonido que altere el comportamiento de las poblaciones o ejemplares de las especies silvestres<br>20. Utilizar lámparas o cualquier fuente de luz para aprovechamiento u observación de ejemplares de la vida silvestre, salvo para actividades de investigación científica y monitoreo científico, sin extracción de flora o fauna<br>21. Confinar materiales y sustancias peligrosas y no peligrosas<br>22. Verter o descargar desechos o cualquier tipo de material nocivo en el suelo, subsuelo y en cualquier clase de cauce, vaso o acuífero

[1] Conforme a lo previsto por el Artículo 2o., fracción VI, del Reglamento de la Ley General de Vida Silvestre.
[2] Conforme a lo previsto por el Artículo 2o., fracción VII, del Reglamento de la Ley General de Desarrollo Forestal Sustentable.
[3] Utilizando ecotecnias y materiales tradicionales de construcción propios de la región.
[4] Conforme a lo establecido en las fracciones XIII y XVII del Artículo 3o., de la Ley General de Vida Silvestre.

Zona de Influencia

Es la superficie aledaña a la poligonal del Área Natural Protegida, abarcando una superficie de 61 mil 243 hectáreas, delimitada a partir de las Unidades de Gestión Ambiental (UGA) del Modelo de Ordenamiento Ecológico Regional adyacentes al territorio del Área Natural Protegida, según el Ordenamiento Ecológico de la Región cuenca del Río Tepalcatepec.

Es importante mencionar la amenaza que constituyen las cabeceras de los municipios de Tancítaro, Peribán de Ramos y San Juan Nuevo, los cuales ejercen una fuerte presión en la Zona de Influencia y al interior del Área Natural Protegida derivada del crecimiento demográfico y cambio de uso de suelo por las actividades económicas del sector primario, principalmente de la producción de aguacate.

Por otro lado, las actividades agrícolas y urbanas que se desarrollan en la Zona de Influencia demandan cada vez más volúmenes de agua provenientes del Área Natural Protegida.

Asimismo, el poblado de Angahuan, del municipio de Uruapan, realiza actividades recreativas y forestales que se extienden a los límites del Área Natural Protegida.

La delimitación de esta Zona de Influencia abarca sitios como cañadas, que constituyen un corredor biológico hacia zonas como el Parque Nacional Barranca del Cupatitzio.

Plano de Localización y Subzonificación del Área de Protección de Flora y Fauna Pico de Tancítaro

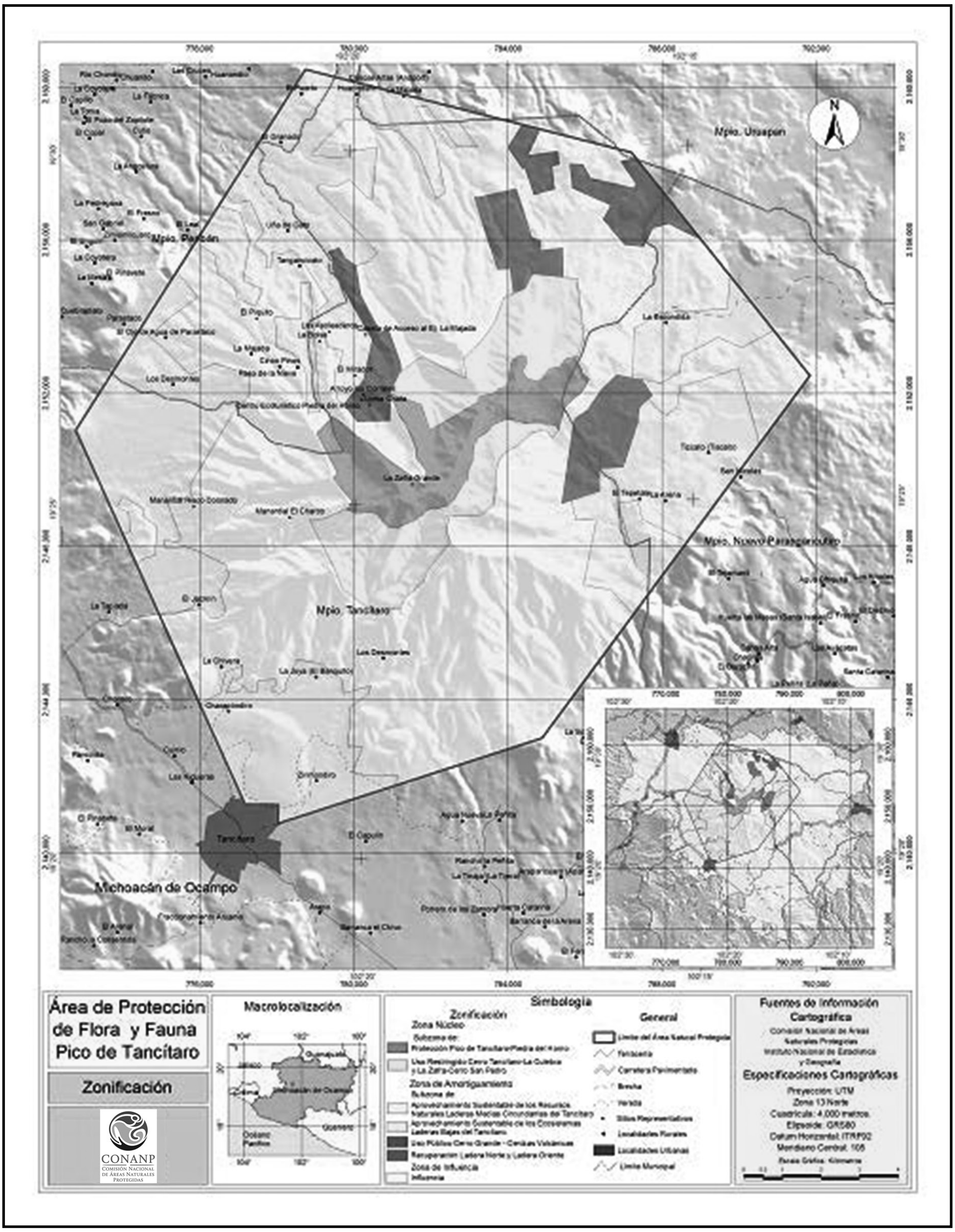

8. REGLAS ADMINISTRATIVAS

Capítulo I

Disposiciones generales

Regla 1. Las presentes Reglas Administrativas son de observancia general y obligatoria para todas las personas físicas o morales que realicen actividades dentro del Área Natural Protegida, con el carácter de Área de Protección de Flora y Fauna Pico de Tancítaro, ubicado en los municipios de Tancítaro, Peribán de Ramos, Nuevo Parangaricutiro y Uruapan, en el estado de Michoacán, con una superficie de 23 mil 405-92-09.55 hectáreas.

Regla 2. La aplicación de las presentes Reglas corresponde a la Secretaría de Medio Ambiente y Recursos Naturales, sin perjuicio de las atribuciones que les correspondan a otras dependencias del Gobierno Federal.

Regla 3. Para los efectos de lo previsto en las presentes Reglas, además de las definiciones contenidas en la Ley General del Equilibrio Ecológico y la Protección al Ambiente y su Reglamento en Materia de Áreas Naturales Protegidas, la Ley General de Vida Silvestre y su Reglamento, se entenderá por:

I. **APFFPT:** El Área Natural Protegida comprendida dentro de la poligonal que establece el Decreto Presidencial publicado en el *Diario Oficial de la Federación* el 19 de agosto de 2009, por el que se declara el Área de Protección de Flora y Fauna Pico de Tancítaro, ubicada en los municipios de Tancítaro, Peribán de Ramos, Nuevo Parangaricutiro y Uruapan, en el estado de Michoacán.

II. **Conanp:** La Comisión Nacional de Áreas Naturales Protegidas, órgano administrativo desconcentrado de la Secretaría de Medio Ambiente y Recursos Naturales.

III. **DIRECCIÓN:** La Unidad Administrativa encargada de la administración del Área de

Protección de Flora y Fauna Pico de Tancítaro, además de coordinar la planeación, ejecución y evaluación del presente Programa de Manejo.

IV. **ECOTECNIA:** El conjunto de técnicas para la producción de vivienda, alimentos y energía, así como para crear nuevas formas de industrialización de los recursos renovables que garantizan una operación limpia, económica y ecológica que puede conseguirse mediante acciones participativas, comunitarias y a través de la armonización de objetivos económicos, sociales y ecológicos.

V. **LAN:** Ley de Aguas Nacionales.

VI. **LGDFS:** Ley General de Desarrollo Forestal Sustentable.

VII. **LGEEPA:** Ley General del Equilibrio Ecológico y la Protección al Ambiente.

VIII. **LGVS:** Ley General de Vida Silvestre.

IX. **Profepa:** Procuraduría Federal de Protección al Ambiente, órgano administrativo desconcentrado de la Secretaría de Medio Ambiente y Recursos Naturales.

X. **REGLAS:** Las presentes Reglas Administrativas.

XI. **Semarnat:** Secretaría de Medio Ambiente y Recursos Naturales.

XII. **TURISMO DE BAJO IMPACTO AMBIENTAL:** Aquella modalidad turística ambientalmente responsable, consistente en viajar o visitar espacios naturales, relativamente sin perturbar, con el fin de disfrutar, apreciar y estudiar los atractivos naturales de dichos espacios, así como cualquier manifestación cultural del presente y del pasado que puedan encontrarse ahí, a través de un proceso que promueve la conservación, tiene bajo impacto ambiental y cultural e induce un involucramiento activo y socioeconómicamente benéfico de las poblaciones locales; dichas actividades son:

a) Ciclismo de montaña: Es la actividad deportiva que se realiza sobre una bicicleta de montaña en terrenos montañosos.

b) Caminatas en senderos interpretativos: Es la actividad que realiza una o un visitante o turista, sobre senderos bordeados de un entorno natural y/o rural.

c) Campismo: Actividad que se realiza al aire libre y que consiste en acampar en tienda de campaña o a la intemperie, durante un periodo de tiempo.

d) Observación de flora y fauna silvestres: Actividad que se realiza al aire libre y que consiste en caminar ciertas distancias para la observación de flora y fauna.

e) Rappel: Actividad que consiste en el descenso por cuerda utilizado en superficies verticales.

XIII. **SENDERO INTERPRETATIVO:** Son pequeños caminos o huellas que permiten recorrer con facilidad un área determinada. Los senderos cumplen varias funciones: servir de acceso y paseo para las y los visitantes, y ser un medio para el desarrollo de actividades educativas y para servir a los propósitos del Área de Protección de Flora y Fauna Pico de Tancítaro.

XIV. **UMA:** Unidades de Manejo para la Conservación de la Vida Silvestre.

XV. **USUARIO:** Persona física o moral que en forma directa o indirecta utiliza o se beneficia de los recursos naturales existentes en el Área de Protección de Flora y Fauna Pico de Tancítaro.

XVI. **VISITANTE:** Persona que se desplaza temporalmente fuera de su lugar de residencia para uso y disfrute del Área de Protección de Flora y Fauna Pico de Tancítaro, durante uno o más días utilizando los servicios de prestadores de servicios turísticos o realizando sus actividades de manera independiente.

Regla 4. Todas las y los usuarios, prestadores de servicios turísticos y visitantes del APFFPT deberán recoger y llevar consigo los residuos sólidos generados durante el desarrollo de sus actividades y depositarlos fuera del APFFPT, en los sitios destinados por la autoridad competente.

Regla 5. Cualquier persona que para el desarrollo de sus actividades dentro del APFFPT requiera concesión, autorización o permiso estará obligada a presentarla cuantas veces le sea requerida ante la Dirección del Área Natural Protegida y la Profepa.

Regla 6. La Dirección podrá solicitar a las y los visitantes o prestadores de servicios turísticos la información que a continuación se describe, con la finalidad de realizar las recomendaciones necesarias en materia de manejo de residuos sólidos, prevención de incendios forestales y protección de los elementos naturales presentes en el área; así como en materia de protección civil y protección al turista:

a. Descripción de las actividades a realizar.

b. Tiempo de estancia.

c. Lugar a visitar.

d. Origen del visitante.

Regla 7. Las y los visitantes del APFFPT deberán cumplir, además de lo previsto en las Reglas Administrativas correspondientes, con las siguientes obligaciones:

I. Cubrir, en su caso, las cuotas establecidas en la Ley Federal de Derechos;

II. Hacer uso exclusivamente de las rutas y senderos establecidos para recorrer el APFFPT;

III. Respetar la señalización, la zonificación y la subzonificación del APFFPT;

IV. Atender las observaciones y recomendaciones formuladas por la Dirección del Área Natural Protegida, relativas a la protección de los ecosistemas del mismo;

V. Brindar el apoyo y las facilidades necesarias para que el personal de la CONANP, la PROFEPA y demás autoridades competentes realicen labores de inspección, vigilancia, protección y control, así como en situaciones de emergencia o contingencia; y,

VI. Hacer del conocimiento del personal de la Dirección del Área Natural Protegida o de la PROFEPA las irregularidades que hubieran observado durante su estancia en el ANP.

CAPÍTULO II

De las autorizaciones, concesiones y avisos

Regla 8. Se requerirá autorización de la SEMARNAT, por conducto de la CONANP, para realizar dentro del APFFPT, atendiendo a las subzonas establecidas, las siguientes actividades:

I. Autorización para realizar actividades turístico-recreativas dentro de Áreas Naturales Protegidas, en todas sus modalidades.

II. Filmaciones, actividades de fotografía, captura de imágenes o sonidos por cualquier medio, con fines comerciales y que requieran equipos compuestos por más de un técnico especializado, como apoyo a la persona que opera el equipo principal.

III. Autorización para realizar actividades comerciales dentro de Áreas Naturales Protegidas.

Regla 9. La vigencia de las autorizaciones a que se refiere la regla anterior será:

I. Por dos años, para la prestación de servicios turísticos;

II. Por el periodo que dure el trabajo, para filmaciones o captura de imágenes o sonidos por cualquier medio, con fines comerciales que requieran más de un técnico especializado; y,

III. Por un año, para actividades comerciales (venta de alimentos y artesanías).

Regla 10. Las autorizaciones emitidas por la SEMARNAT, por conducto de la CONANP, para la realización de actividades turísticas recreativas o para la venta de alimentos y artesanías dentro de APFFPT podrán ser prorrogadas por el mismo periodo por el que fueron otorgadas, conforme a las disposiciones jurídicas aplicables.

Regla 11. Con la finalidad de proteger los recursos naturales del APFFPT y brindar el apoyo necesario, previamente la o el interesado deberá presentar a la Dirección del Área Natural Protegida un aviso, para realizar las siguientes actividades:

I. Investigación sin colecta o manipulación de ejemplares de especies no consideradas en riesgo.

II. Educación ambiental que no implica ninguna actividad extractiva dentro del Área Natural Protegida.

III. Monitoreo sin colecta o manipulación de especímenes de especies no consideradas en riesgo.

IV. Filmaciones, actividades de fotografía, la captura de imágenes o sonidos por cualquier medio con fines científicos, culturales o educativos, que requieran equipos compuestos por más de un técnico especializado como apoyo a la persona que opera el equipo principal.

V. Aviso para realizar actividades de investigación con colecta o manipulación de ejemplares de flora y fauna silvestres. Independientemente del aviso a que se refiere esta fracción, el interesado o interesada deberá contar con la autorización correspondiente en términos de la LGVS y su Reglamento.

Regla 12. Se requerirá la autorización emitida por la SEMARNAT, a través de sus distintas Unidades Administrativas, para la realización de las siguientes actividades:

I. Aprovechamiento extractivo de ejemplares, partes y derivados de la vida silvestre;

II. Aprovechamiento de recursos forestales maderables en terrenos forestales o preferentemente forestales;

III. Aprovechamiento de recursos forestales no maderables;

IV. Colecta de recursos biológicos forestales, con fines científicos;

V. Colecta de ejemplares, partes y derivados de vida silvestre con fines de investigación científica y propósitos de enseñanza, en todas sus modalidades;

VI. Manejo, control y remediación de problemas asociados a ejemplares y poblaciones que se tornen perjudiciales;

VII. Obras y actividades en Áreas Naturales Protegidas de competencia de la Federación: que requieren una Evaluación de Impacto Ambiental, y

VIII. Registro y renovación de Unidades de Manejo para la Conservación de la Vida Silvestre (UMA).

Regla 13. Se requerirá la concesión del Ejecutivo Federal, a través de la Comisión Nacional del Agua, para la realización de las siguientes actividades:

I. Aprovechamiento de aguas superficiales, y

II. Aprovechamiento de aguas subterráneas, conforme a lo previsto por los artículos 18, primer párrafo y 42, fracción I de la LAN.

Regla 14. Para la obtención de las concesiones, autorizaciones, permisos y prórrogas a que se refiere en el presente capítulo, el interesado o interesada deberá cumplir con los términos y requisitos establecidos en las disposiciones legales y reglamentarias aplicables.

Capítulo III

De los prestadores de servicios turísticos

Regla 15. Las y los prestadores de servicios turísticos que pretendan desarrollar actividades turísticas dentro del APFFPT deberán cerciorarse de que su personal y las y los visitantes que contraten sus servicios cumplan con lo establecido en la presentes Reglas.

La Dirección del Área Natural Protegida no se hará responsable por los daños que sufran los visitantes o usuarios en sus bienes, equipos o integridad física, ni de aquellos causados a terceros durante la realización de sus actividades dentro del APFFPT.

Regla 16. Las y los prestadores de servicios turísticos se obligan a informar a las y los usuarios que están ingresando a un Área Natural Protegida, en la cual se desarrollan acciones para la conservación de los recursos naturales y la preservación del entorno natural; asimismo, deberán hacer de su conocimiento la importancia de su conservación y la normatividad que deberán acatar durante su estancia, pudiendo apoyar esa información con material gráfico y escrito.

Regla 17. Las y los prestadores de servicios deberán contar con un seguro de responsabilidad civil y de daños a terceros, con la finalidad de responder de cualquier daño o perjuicio que sufran en su persona o en sus bienes las y los visitantes, así como de los que sufran los vehículos y equipo, o aquellos causados a terceros durante su estancia y desarrollo de actividades en el APFFPT.

Regla 18. El uso turístico y recreativo dentro del APFFPT se llevará a cabo bajo los criterios establecidos en el instrumento y siempre que:

I. De acuerdo al concepto básico de turismo de bajo impacto ambiental, no se provoque una alteración significativa a los ecosistemas;

II. Preferentemente tengan un beneficio directo para las y los pobladores locales, y

III. Promueva la educación ambiental.

Regla 19. Los grupos de visitantes podrán contratar a una o un guía, preferentemente local, quien será responsable del grupo. La o el guía deberá cumplir, según corresponda, con lo establecido en las siguientes Normas Oficiales Mexicanas:

- Norma Oficial Mexicana NOM-08-TUR-2002, Que establece los elementos a que deben sujetarse los guías generales y especializados en temas o localidades específicas de carácter cultural.

- Norma Oficial Mexicana NOM-09-TUR-2002, Que establece los elementos a que deben sujetarse los

guías especializados en actividades específicas.

- Norma Oficial Mexicana NOM-011-TUR-2001, Requisitos de seguridad, información y operación que deben cumplir los prestadores de servicios turísticos de Turismo de Aventura.

Capítulo IV

De los visitantes

Regla 20. Las actividades de campismo dentro del APFFPT se podrán realizar únicamente dentro de las Subzonas: de Uso Restringido, Aprovechamiento Sustentable de Recursos Naturales, Aprovechamiento Sustentable de los Ecosistemas y Uso Público, y estarán sujetas a las siguientes restricciones:

I. Excavar, nivelar, cortar o desmontar la vegetación del terreno donde se acampe, y

II. Erigir instalaciones permanentes de campamento.

Regla 21. Las fogatas podrán realizarse únicamente en la Subzona de Uso Público, siempre y cuando se realicen de conformidad con la Norma Oficial Mexicana Nom-015-Semarnat/SAGARPA-2007, Que establece las especificaciones técnicas de métodos de uso del fuego en los terrenos forestales y en los terrenos de uso agropecuario.

Regla 22. Para la disposición de residuos de origen orgánico, tales como aguas grises y materia fecal, los visitantes deberán utilizar las técnicas apropiadas, tales como “hoyo de gato” para enterrarlos, evitando en todo momento enterrar el papel, así como el fecalismo al aire libre.

Capítulo V

De la investigación científica

Regla 23. Todo investigador o investigadora que ingrese al APFFPT con el propósito de realizar colecta con fines científicos deberá notificar a la Dirección del Área Natural Protegida sobre el inicio de sus actividades, de conformidad con lo establecido en la fracción V de la Regla 11, adjuntando una copia de la autorización con la que se cuente; de igual manera, deberá informar al mismo del término de sus actividades y hacer llegar a la Dirección del Área Natural Protegida una copia de los informes exigidos en dicha autorización.

Regla 24. Con el objeto de garantizar la correcta realización de las actividades de investigación científica y salvaguardar la integridad de los ecosistemas y de los investigadores, estos últimos deberán sujetarse a los lineamientos y condicionantes establecidos en la autorización respectiva, y observar lo dispuesto en el Decreto de establecimiento del APFFPT, el presente Programa de Manejo, la Norma Oficial Mexicana Nom-126-Semarnat-2000, Por la que se establecen las especificaciones para la realización de actividades de colecta científica de material biológico de especies de flora y fauna silvestres y de otros recursos biológicos en el territorio nacional, las presentes Reglas y demás disposiciones jurídicas aplicables.

Regla 25. Los investigadores no podrán extraer partes del acervo cultural e histórico del APFFPT, así como ejemplares de flora, fauna, suelo, fósiles, rocas o minerales, salvo que cuenten con la autorización por parte de las autoridades correspondientes.

Regla 26. Las actividades de colecta científica se llevarán a cabo con el consentimiento previo, expreso e informado de la o el propietario o poseedor legítimo del predio en donde ésta pretenda realizarse.

Regla 27. Las actividades de colecta científica estarán restringidas a los sitios y especies especificadas en la autorización correspondiente y con apego a la subzonificación establecida en el presente instrumento; en caso de organismos capturados incidentalmente, deberán ser liberados en el sitio de la captura.

Regla 28. Quienes realicen actividades de colecta científica de vida silvestre dentro del APFFPT deberán destinar al menos un duplicado del material biológico colectado a instituciones o colecciones científicas mexicanas, en términos de lo establecido por la LGVS.

Regla 29. Las autorizaciones de colecta no amparan el aprovechamiento para fines comerciales ni de utilización en biotecnología; en caso contrario, se regirán por las disposiciones aplicables en la Ley General de Vida Silvestre, la Ley General de Desarrollo Forestal Sustentable y demás instrumentos legales aplicables.

Regla 30. El desarrollo de actividades relacionadas con la investigación científica y el monitoreo de los ecosistemas del Área se llevará a cabo para la evaluación, recuperación y conservación de los recursos existentes en ésta.

Regla 31. El establecimiento de campamentos para actividades de investigación quedará sujeto a los términos especificados en la autorización, así como al cumplimiento de lo previsto por la Regla 20.

Capítulo VI

De los usos y aprovechamientos

Regla 32. El aprovechamiento de leña para uso doméstico deberá provenir de arbolado muerto. Asimismo, esta actividad deberá sujetarse a lo establecido por la LGDFS y su Reglamento, así como lo previsto en la Norma Oficial Mexicana Nom-012-Semarnat-1996, Que establece los procedimientos, criterios y especificaciones para realizar el aprovechamiento, transporte y almacenamiento de leña para uso doméstico.

Regla 33. La reforestación de áreas degradadas o aquellas cuyo uso del suelo esté destinado al aprovechamiento forestal se realizará preferentemente con especies nativas.

Regla 34. Las actividades de aprovechamiento de flora y fauna silvestres, así como el establecimiento y funcionamiento de la UMA, deberán realizarse conforme a las disposiciones legales establecidas en la LGEEPA, la LGVS, la LGFDS y sus reglamentos,

así como a las Normas Oficiales Mexicanas aplicables, garantizando así la permanencia y reproducción de las especies aprovechadas.

Regla 35. La construcción de infraestructura, en las subzonas donde se permita, será acorde con el entorno natural del APFFPT, empleando preferentemente ecotecnias, materiales tradicionales de construcción propios de la región, así como diseños que no destruyan ni modifiquen el paisaje ni los recursos naturales, evitando la dispersión de residuos, y deberá cumplir las disposiciones legales aplicables.

Regla 36. El mantenimiento de caminos ya existentes podrá llevarse a cabo, siempre que éstos no se amplíen.

Regla 37. El aprovechamiento de los recursos forestales se realizará de manera integral y sustentable, garantizando la preservación y reforestación de los mismos.

Reglas 38. El desarrollo de actividades de turismo será de bajo impacto ambiental, de tal manera que se respete la integridad del ecosistema.

Reglas 39. El desarrollo de actividades de agricultura, agroforestería y silvopastoriles se realizará de manera tal que sea compatible con la conservación del ecosistema.

Regla 40. En el APFFPT solo se permitirán actividades con Organismos Genéticamente Modificados (OGM) para fines de biorremediación, en los casos en que aparezcan plagas o contaminantes que pudieran poner en peligro la existencia de especies animales, vegetales o acuícolas, y los OGM hayan sido creados para evitar o combatir dicha situación siempre que se cuente con los elementos científicos y técnicos necesarios que soporten el beneficio ambiental que se pretende obtener, y dichas actividades sean permitidas por la Semarnat en los términos de la Ley de Bioseguridad.

Capítulo VII

De la subzonificación

Regla 41. Con la finalidad de conservar los ecosistemas y la biodiversidad del APFFPT, así como delimitar y ordenar territorialmente la realización de actividades dentro del mismo, se establecen las siguientes subzonas:

I. Zona Núcleo. Conformadas por las subzonas:

   a) **De Protección Pico de Tancítaro-Piedra del Horno**, con una extensión de mil 080.563357 hectáreas, conformada por un polígono, y

   b) **De Uso Restringido Cerro Tancítaro-La Culebra y La Zafra-Cerro San Pedro**, con una superficie de mil 709.738968 hectáreas, integrada por dos polígonos.

II. **Zona de Amortiguamiento**. Conformadas por las subzonas:

   a) **De Aprovechamiento Sustentable de los Recursos Naturales Laderas Medias**

Circundantes del Tancítaro, abarca una superficie de 12 mil 286.690369 hectáreas, constituida por cuatro polígonos;

b) **De Aprovechamiento Sustentable de los Ecosistemas Laderas Bajas del Tancítaro**, abarca una superficie de seis mil 889.181620 hectáreas, comprendida en siete polígonos;

c) **De Uso Público Cerro Grande-Cenizas Volcánicas**, abarca una superficie de 730.600124 hectáreas, constituida por tres polígonos, y

d) **De Recuperación Ladera Norte y Ladera Oriente**, abarca una superficie de 709.146517 hectáreas, constituida por dos polígonos.

Regla 42. El desarrollo de las actividades permitidas y no permitidas dentro de las subzonas mencionadas en la Regla anterior, se sujetará a lo previsto en el apartado denominado Zonificación y Subzonificación, del presente instrumento.

Capítulo VIII

De las actividades prohibidas

Regla 43. En la Zona Núcleo del Área de Protección de Flora y Fauna Pico de Tancítaro queda prohibido:

I. Verter o descargar contaminantes en el suelo, subsuelo y cualquier clase de cause, vaso o acuífero, así como desarrollar cualquier actividad contaminante;

II. Interrumpir, rellenar, desecar o desviar flujos hidráulicos;

III. Realizar actividades cinegéticas o de explotación y aprovechamiento de especies de flora y fauna silvestres y extracción de tierra de monte y su cubierta vegetal;

IV. Introducir organismos genéticamente modificados;

V. Cambiar el uso de suelo y,

VI. Hacer uso de explosivos.

Regla 44. Dentro de la Zona de Amortiguamiento del Área de Protección de Flora y Fauna Pico de Tancítaro queda prohibido:

I. Verter o descargar contaminantes, deshechos o cualquier tipo de material nocivo en el suelo, el subsuelo y cualquier clase de cauce, vaso o acuífero;

II. Introducir especies exóticas;

III. Tirar o abandonar residuos; y,

IV. Construir confinamientos para materiales y sustancias peligrosas.

Capítulo IX

De la inspección y vigilancia

Regla 45. La inspección y vigilancia del cumplimiento de las presentes

Reglas, corresponde a la Semarnat por conducto de la Profepa, sin perjuicio de las atribuciones que correspondan a otras dependencias del Ejecutivo Federal.

Regla 46. Toda persona que tenga conocimiento de alguna infracción o ilícito que pudiera ocasionar algún daño a los ecosistemas del APFFPT deberá notificar a las autoridades competentes de dicha situación, por conducto de la Profepa o la Dirección del Área Natural Protegida, con el objeto de realizar las gestiones correspondientes.

Capítulo X

De las sanciones y recursos

Regla 47. Las violaciones al presente instrumento serán sancionadas de conformidad con lo dispuesto en la LGEEPA y sus reglamentos, así como en el Título Vigésimo Quinto del Código Penal Federal y demás disposiciones legales y reglamentarias aplicables.

9. PROGRAMA OPERATIVO ANUAL

El Programa Operativo Anual (POA) es un instrumento de planeación a través del cual se expresan los objetivos y las metas a alcanzar en un periodo anual.

A través del POA es posible organizar las actividades a realizar en el Área Natural Protegida durante el periodo seleccionado, considerando para ello el presupuesto a ejercer en su operación.

Este instrumento constituye también la base sobre la cual la Conanp podrá negociar el presupuesto para cada ciclo, considerando las necesidades y expectativas de cada una de las áreas.

Con la planeación de las actividades será posible llevar a cabo el seguimiento y la evaluación de acciones, lo que a su vez permite hacer ajustes y tomar medidas orientadas a propiciar la mejora continua de la institución.

Metodología

Para la elaboración del POA, la Dirección del Área Natural Protegida deberá observar las acciones contenidas en los componentes del Programa de Manejo (PM), las cuales se encuentran temporalizadas en corto, mediano y largo plazos, para seleccionar las acciones que habrán de ser iniciadas y cumplidas en el periodo de un año. Se deberá considerar que, aun cuando haya acciones a mediano o largo plazos, algunas de ellas deberán tener inicio desde el corto plazo.

Para definir prioridades en cuanto a las acciones a ejecutar se propone la utilización de la metodología de "Planificación de Proyectos Orientada a Objetivos" (Ziel Orientierte Projekt Plannung-ZOPP).

La planificación toma forma a través de un "marco lógico", en el que se presentan objetivos, resultados y actividades, al mismo tiempo que los indicadores que permitirán medir el avance del resultado estratégico. Desde esta perspectiva, los componentes que darán consistencia al POA serán acordes al presente Programa de Manejo.

Características del POA

El POA consta de siete apartados que deberán respetar lo dispuesto en el PCM, utilizando para ello los formatos que al efecto elabore la Dirección de Evaluación y Seguimiento (DES) de la Conanp y que atiendan a los siguientes criterios:

- Datos generales del Área Natural Protegida, en los que se describen las características generales del área.
- Antecedentes, en los que se enumeran los principales resultados obtenidos dentro del área.
- Diagnóstico, consistente en la identificación de fortalezas, oportunidades, debilidades y amenazas que enfrenta el Área Natural Protegida.
- La matriz de planeación, o marco lógico, en la que se plasman los objetivos, estrategias y metas a alcanzar a lo largo del periodo de un año.
- La descripción de actividades, que permitirán la concreción de los objetivos.
- Los proyectos que conforman el POA, desglosando las actividades de cada uno. Es importante mencionar que los títulos de los proyectos se definirán en función del anexo temático incluido en el formato.
- La matriz de fuente de recursos por actividad y/o acción, que permitirá identificar las aportaciones de cada una de las instituciones u organizaciones involucradas en el desarrollo del POA, así como el costo total de cada una de las actividades.

Proceso de definición y calendarización

Como se mencionó anteriormente, el POA constituye no solo una herramienta de planeación, sino también de negociación del presupuesto, por lo que será necesario que se elabore por parte del cuerpo técnico del Área Natural Protegida y de la Dirección Regional respectiva durante los meses establecidos en la tabla de calendarización.

Una vez elaborado, cada POA será analizado por la DES, así como por las áreas técnicas de Oficinas Centrales, quienes emitirán su opinión respecto de las actividades propuestas. Los resultados del análisis serán remitidos al área generadora para su actualización.

Con la elaboración y entrega de los POA en forma oportuna será posible alinear los objetivos, las actividades y las unidades de medida hacia los objetivos y metas institucionales.

En virtud de que en el proceso de análisis intervienen las distintas áreas de Oficinas Centrales, con el propósito de evitar retrasos en la integración de la información, se definió el siguiente calendario, atendiendo a los criterios de regionalización con los que opera la Conanp.

La Dirección del Área Natural Protegida o la Dirección Regional			
**Entregará a la Dirección Regional la propuesta de POA**	**Entregará a las Oficinas Centrales la propuesta de POA Regional**	**Recibirá observaciones de Oficinas Centrales**	**Entregará el POA en forma definitiva**
1ª semana de octubre	3ª semana de octubre	1ª semana de enero	1ª quincena de febrero

Seguimiento y evaluación del Programa Operativo Anual

A fin de constatar los avances en el desarrollo del Programa Operativo Anual se han establecido fechas para la elaboración de los reportes de avances de las acciones programadas, que deberán ser requisitados en los formatos que al efecto elabore la DES y remitidos para su integración al Sistema de Información, Monitoreo y Evaluación para la Conservación (SIMEC), con un periodicidad trimestral (con excepción del 4º trimestre), de conformidad con el siguiente calendario:

Trimestre	Fechas de entrega	
	**Anp**	**Región**
Enero-marzo	Primeros 10 días hábiles de abril	Primeros 20 días hábiles después de terminado cada trimestre
Abril-junio	Primeros 10 días hábiles de julio	
Julio-septiembre	Primeros 10 días hábiles de octubre	
Octubre-diciembre	Primeros 10 días hábiles de enero	

Los informes deberán reflejar las actividades, unidades de medida y metas planteadas para el periodo en cuestión; toda vez que se trata de reportes oficiales, deberán ser firmados por el responsable de información o titular del área.

La información proporcionada trimestralmente permitirá elaborar, entre otros, los informes de gestión y desempeño institucional que en forma periódica son requeridos por la Subsecretaría de Planeación y Política Ambiental, por la Contraloría Interna de Semarnat y por la Dirección General de Programación y Presupuesto (DGPP), entre otros.

10. EVALUACIÓN DE LA EFECTIVIDAD DEL MANEJO

Proceso de evaluación

La evaluación se realizará en dos vertientes:

1. Programa de Manejo.

2. Programa Operativo Anual.

La evaluación del Programa de Manejo del Área de Protección de Flora y Fauna Pico de Tancítaro es fundamental, ya que al constituirse como el documento rector que incluye las líneas estratégicas y de planeación que deben ser realizadas en un periodo determinado, es importante evaluar su aplicación, atendiendo a cada uno de los subprogramas y componentes desarrollados en este instrumento, así como a las metas e indicadores correspondientes.

Conforme a lo previsto en el Artículo 77 y demás correlativos del Reglamento de la Ley General del Equilibrio Ecológico y la Protección al Ambiente en Materia de Áreas Naturales Protegidas, el Programa de Manejo del Área de Protección de Flora y Fauna Pico de Tancítaro será revisado por lo menos cada cinco años con el objeto de evaluar su efectividad y proponer posibles modificaciones. Para ello, la Dirección del Área Natural Protegida, deberá atender el procedimiento previsto en los Lineamientos Internos para la Formulación, Revisión y Modificación de Programas de Manejo de las Áreas Naturales Protegidas competencia de la Federación, establecidos por la Conanp.

El Programa de Manejo podrá ser modificado en todo o en parte, cuando resulte inoperante para el cumplimiento de los objetivos del Área de Protección de Flora y Fauna Pico de Tancítaro, para lo cual la Dirección del Área Natural Protegida, deberá solicitar la opinión del Consejo Asesor del Área.

Previo análisis y opinión del Consejo Asesor, se podrá modificar el presente Programa de Manejo cuando:

I. Las condiciones naturales y originales del área hayan cambiado debido a la presencia de fenómenos naturales y se requiera el planteamiento de estrategias y acciones distintas a las establecidas en el Programa vigente;

II. Técnicamente se demuestre que no pueden cumplirse estrategias o acciones establecidas en el Programa vigente, o

III. Técnicamente se demuestre la necesidad de adecuar la delimitación, extensión o ubicación de las subzonas delimitadas.

Las modificaciones al Programa de Manejo que resulten necesarias deberán seguir el mismo procedimiento establecido para su elaboración y un resumen de las mismas se publicará en el *Diario Oficial de la Federación*.

La ejecución del Programa de Manejo se realizará a través de los Programas Operativos Anuales (POA) que defina la Dirección del Área Natural Protegida. Esto es, que año con año la propia Dirección deberá establecer las líneas a abordar y los resultados que espera obtener durante el periodo.

Anualmente, se contrastarán los avances logrados en la operación del Área Natural Protegida contra las metas propuestas en el Programa de Manejo; al término del primer quinquenio de operación se revisarán la totalidad de los subprogramas a fin de determinar los aspectos que por razones políticas, sociales, económicas y/o administrativas pudiesen haber quedado pendientes de realización. Mediante este tipo de evaluación se construirán las series históricas de avances, lo que permitirá la proyección de las acciones a desarrollar en los siguientes cinco años.

Con base en la información proporcionada trimestralmente sobre el cumplimiento de metas del POA, se realizarán las evaluaciones relativas al desempeño institucional (cumplimiento o incumplimiento de metas, calidad en la realización de acciones) y la gestión (aplicación del gasto).

11. BIBLIOGRAFÍA

Aguilera Montañez, J. L. y S. Salazar García, *The Avocado industry in Michoacan Mexico*, Ybrk, South African Avocado Growers Association, 1991, 14, 94-97.

Anguiano, C. J.; R. J. J. Alcántar; C. Y. Rodríguez; C. J. A. Ruíz; V. L. M. Tapia; B. R. Toledo, *Caracterización edafo-climática del área productora de aguacate de Michoacán*, México, INIFAP, CIRPAC, Campo Experimental Uruapan, México, 2006 (Libro Técnico No. 4).

Alvarado Díaz, J. y J. A. Campbell, *A new montane ratlesnake (Viperidae) from Michoacán, México*, en *Herpetológica,* 2004, 60(2), 281-286.

AOU, *American Ornithologist's Union. Check-list of North American Birds,* 7a ed., 1998, 829.

AOU, *Forty-second supplement to the American Ornithologist's Union Check-list of North American Birds*, en *Auk,* 2000, 117, 847-858.

Arriaga, L., C. Aguilar, J. M. Espinoza, L. Gómez, E. Loa y E. Martínez, coordinadores, *Regiones terrestres prioritarias de México,* México, Comisión Nacional para el Conocimiento y uso de la Biodiversidad. 2002, 609.

Arizmendi, M. C. y L. Márquez Valdelamar, *Áreas de importancia para la conservación de las aves en México,* México, CIPAMEX, 2000.

Banks, R. C.; C. Cicero; J. L. Dunn; A. W. Kratter; P. C. Rasmussen *et al., Forty-third supplement to the American Ornithologist's Union. Check-list of North American Birds*, en *Auk,* 2002, 119(3), 897-906.

Banks, R. C.; C. Cicero; J. L. Dunn, A. W. Kratter, P. C. Rasmussen *et al., Forty-fourth supplement to the American Ornithologist's Union. Check-list of North American Birds*, en *Auk,* 2003, 120(3), 923-931.

Banks, R. C.; C. Cicero; J. L. Dunn; A. W. Kratter; P. C. Rasmussen *et al., Forty-fifth supplement to the American Ornithologist's Union. Check-list of North American Birds*, en *Auk,* 2004, 121(3), 985-995.

Banks, R. C.; C. Cicero; J. L. Dunn; A. W. Kratter; P. C. Rasmussen *et al., Forty-sixth supplement to the American Ornithologist's Union. Check-list of North American Birds*, en *Auk,* 2005, 122(3), 1026-1031.

Banks, R. C.; C. Cicero; J. L. Dunn; A. W. Kratter; P. C. Rasmussen *et al., Forty-seventh supplement to the American Ornithologist's Union. Check-list of North American Birds*, en *Auk,* 2006, 123(3), 926-936.

Banks, R. C.; R. T. Chesser; C. Cicero; J. L. Dunn; A. W. Kratter *et al., Forty-eighth supplement to the American Ornithologist's Union. Check-list of North American Birds*, en *Auk,* 2007, 124(3), 1109-1115.

Banks, *R. C.; R. T. Chesser; C. Cicero; J. L. Dunn; A. W.* Kratter *et al., Forty-ninth supplement to the American Ornithologist's Union. Check-list of North American Birds*, en *Auk,* 2008, 125(3), 758-768.

Barsimantov, J. y A. J. Navia Antezana, *Land use and land tenure change in Mexico's avocado production region: can community forestry reduce incentives to deforest for high value crops?*, en *Twelfth biennial conference of the International Association for the Study of the Commons*, Cheltenham, United Kingdom, 2008.

Blake, E. R. y H. Hanson, *Notes on a collection of birds from Michoacán, Mexico*, en *Field Mus Nat Hist,* 1942, XXII 22(9), 513-551.

Bocco, G. y C. Garibay, *Legislación Ambiental y Áreas Naturales Protegidas*. El caso de la microrregión del Pico de Tancítaro, Mich., Informe Técnico Final, Morelia, Profepa-SEMARNAP, 2000.

Bocco, G.; J. Fuentes y C. Garibay, *2º Informe Técnico*, Presentado a Profepa-CONACyT, 1999.

Bocco, G. y J. F. Mass, Proyecto: *Geomorfología, paisaje y recursos naturales en el Parque Nacional Pico de Tancítaro*, Michoacán, 2001-2003, PAPIIIT-DGPA-UNAM.

Bocco, G. y M. Mendoza, *Análisis del cambio del uso del suelo en el estado de Michoacán*, Instituto de Ecología-UNAM, SIMORELOS (CONACYT-Regional), 1999, disponible en www.oikos.unam.mx/laboratorios/geoecologia/ES/PROYECTOS/CAMBIO_USO_MICH.HTML.

Bocco, G.; Mendoza, M. y A. Velásquez, *Remote sensing and GIS-based regional geomorphologic mapping. A tool for land use planning in developing countries*, en *Geomorphology,* 2001, 39, 211-219.

Ceballos, G. y D. Navarro, *Diversity and conservation of Mexican mammals*, en Mares M. A. y D. J. Schmidly, editores, *Topics in Latin American mammalogy: history, biodiversity, and education,* Oklahoma, University of Oklahoma Press, 1991, 167-198.

Ceballos, G. y A. Miranda, *Guía de campo de los mamíferos de la costa de Jalisco, México*, México, Fundación Ecológica Cuixmala, 2000.

Ceballos, G. y G. Oliva, *Los mamíferos silvestres de México,* 1a ed., Fondo de Cultura Económica, Comisión para el Conocimiento y Uso de la Biodiversidad, 2005, 986.

Chávez, L. G.; J. J. Alcántar Rocillo; J. Anguiano Contreras y R. Toledo Bustos, *Expansión del cultivo del aguacate y deforestación en Michoacán*, en Conferencia de Usuarios de SIGSA ESRI, México, 27 a 29 de agosto de 2008.

Chesser, R. T.; R. C. Banks; F. K. Barker; C. Cicero; J. L. Dunn *et al., Fiftieth supplement to the American Ornithologist's Union. Check-list of North American Birds*, en *Auk,* 2009, 126(3), 705-714.

Chesser, R. T.; R. C. Banks; F. K. Barker; C. Cicero; J. L. Dunn *et al., Fifty-first supplement to the American Ornithologist's Union. Check-list of North American Birds*, en *Auk,* 2010, 127(3), 726-744.

Chesser, R. T.; R. C. Banks; F. K. Barker; C. Cicero; J. L. Dunn *et al., Fifty-second supplement to the American Ornithologist's Union. Check-list of North American Birds*, en *Auk,* 2011, 128(3), 600-613.

Chiappy, C., *Modificaciones ecológico-paisajísticas; perspectivas en el planeamiento territorial y la preservación de la Biodiversidad,* Tesis de Maestría, México, Facultad de Ciencias, Universidad Nacional Autónoma de México, 1996, 132.

Cincotta, R. P.; R. Engelman y J. Winsnewski, *Human population in the biodiversity hotspots*, en *Nature,* 2000, 404, 990-991.

Conabio, *La diversidad biológica de México: Estudio de País*, 1998, México, Comisión Nacional para el Conocimiento y Uso de la Biodiversidad. 1998, 293.

Conabio, *Regiones Prioritarias Terrestres. Escala 1:1´000,000*, México, 1999.

Conanp-Comisión Nacional de Áreas Naturales Protegidas, *Protocolo para la evaluación del uso del suelo y vegetación en Áreas Naturales Protegidas Federales de México*, México, Dirección de evaluación y seguimiento. Subdirección de análisis e información espacial, 2007.

CONANP-Comisión Nacional de Áreas Naturales Protegidas, *Estudio Previo Justificativo para la recategorización del Parque Nacional Pico de Tancítaro como Área de Protección de Flora y Fauna, Michoacán*, México, Comisión Nacional de Áreas Naturales Protegidas, 2008, 88 (más 3 anexos).

CONANP-Comisión Nacional de Áreas Naturales Protegidas, *Programa Nacional de Áreas Naturales Protegidas, 2007-2012*.

Diario Oficial de la Federación, NORMA OFICIAL MEXICANA NOM-059-SEMARNAT-2010, Protección ambiental-Especies nativas de México de flora y fauna silvestres-Categorías de riesgo y especificaciones para su inclusión, exclusión o cambio-Lista de especies en riesgo, diciembre de 2010.

Diario Oficial de la Federación, Decreto por el que se declara Área Natural Protegida, con el carácter de Área de Protección de Flora y Fauna, la región denominada Pico de Tancítaro, ubicada en los municipios de Tancítaro, Peribán de Ramos, Nuevo Parangaricutiro y Uruapan, en el estado de Michoacán, 19 de agosto de 2009.

Echánove, H., *Abriendo fronteras: el auge exportador del aguacate mexicano a Estados Unidos*, en *Anales de Geografía,* 2008, 28(1), 9-28.

Enciclopedia de los municipios de México, Michoacán, Instituto Nacional para el Federalismo y el Desarrollo Municipal Gobierno del estado de Michoacán, 2009.

Escalante P. P.; J. Robles Gil y A. M. Sada, *Listado de nombres comunes de las aves de México,* CONABIO y Sierra Madre, 1996, 32.

Estrada Virgen, A., *Catálogo de la diversidad de anfibios y reptiles del municipio de Tancítaro, Michoacán, México,* Tesis de Licenciatura, Facultad de Biología, Universidad Michoacana de San Nicolás de Hidalgo, 2001.

Flores, V. O. y P. Gerez, *Biodiversidad y conservación en México: vertebrados, vegetación y uso del suelo,* México, CONABIO-UNAM, 1994, 439.

Fragoso, P. I. L., *Estimación del contenido y captura de carbono en biomasa aérea del predio "Cerro Grande" municipio de Tancítaro Michoacán México,* Tesis de Licenciatura, México, Facultad de Agrobiología "Presidente Juárez", UMSNH, 2003.

Fuentes, J. J. A., *Evaluación del deterioro en Áreas Naturales Protegidas. Un enfoque geomorfológico. El caso del Parque Nacional Pico de Tancítaro, Michoacán,* Tesis para obtener el grado Académico de maestro en geografía, México, Facultad de Filosofía y Letras, UNAM, 2000.

Fuentes, J. J. A. y G. Bocco, *El agua: dinámica y análisis regional*, en Velásquez A., A. Torres y G. Bocco G., *com. pers., Las enseñanzas de San Juan. Investigación participativa para el manejo integral de recursos naturales,* México, Instituto Nacional de Ecologia-SEMARNAP, Gobierno del estado de Michoacán, 2003, 603.

Fuentes, J. J. A. y L. F. Alvarado, *Propuesta de manejo sustentable de ecosistemas de montaña,* Comisión Nacional Forestal, Gerencia Regional, Montaña Prioritaria Pico de Tancítaro, Semarnat-Conafor, 2006.

Fuentes, J. J. A., resp., *Ordenamiento Ecológico del Territorio Municipio de Tancítaro, Mich.*, Fase de diagnóstico, Informe final, Centro de Investigaciones en Ecosistemas, Universidad Nacional Autónoma de México, Campus Morelia, 2009, 124.

García, E., *Apuntes de climatología,* México, Facultad de Ciencias, UNAM, 1984, 86.

García, I., *Informe Final del proyecto H304, Flora del Parque Nacional Pico de Tancítaro, Michoacán*, Comisión Nacional para el Conocimiento y Uso de la Biodiversidad, 1998, disponible en www.conabio.gob.mx.

García, R. I.; B. Cházaro; R. Flores; N. Machuca; V. J. Nava y N. del Río, *Flora del Parque Nacional Pico de Tancítaro, Michoacán*, Informe Técnico Final, CIDIR-IPN- Conabio, 1998, 99.

García Ruiz, I., *Flora del Parque Nacional Pico de Tancítaro, Michoacán*, México, Instituto Politécnico Nacional, Centro Interdisciplinario de Investigación para el Desarrollo Integral Regional-Michoacán, Bases de datos SNIB-Conabio proyecto No. H304, 1999.

Garduño Monroy, V. H.; E. Arreygye; B. Bigioggero; S. Chiesa; C. P. Corona *et al., Carta geológica del estado de Michoacán, Escala 1:250,000,* Morelia, Universidad Michoacana de San Nicolás Hidalgo, Instituto de Investigación Metalúrgicas, 1999, 111.

Garibay C. y G. Bocco, *Algunos casos de control y manejo de recursos en el Parque Nacional Pico de Tancítaro*, Avances de investigación presentados ante la XII Mesa de Trabajo, Región, localidad y transformación del paisaje mexicano, Estudios etnográficos, Colegio de Michoacán, 23 y 24 de septiembre de 1999, 249.

Garibay C. y G. Bocco, *Legislación ambiental, áreas protegidas y manejo de recursos en zonas indígenas forestales*. El Caso de la Microregión del Pico de Tancítaro, Michoacán, Fondo Mexicano para la Conservación de la Naturaleza, Profepa-CONACYT, 2000.

García, C.; I. García; M. A. Chávez Carbajal, F. Guevara Fefer, M. A. Martínez; P. Silva Sáenz, *Estudio florístico en el área de la comunidad indígena de Nuevo San Juan Parangaricutiro, Michoacán, México*, en *Acta Botánica Mexicana,* 2000, 52, 5-41.

García, R. I.; B. Chazado; R. Flores; N. Machuca; V. J. Nava y N. del Río, Flora del Parque nacional Pico de Tancítaro, Michoacán, Informe Técnico Final, CIIDIR-IPN- Conabio, 1998, 99.

Goldman, E. A., *Biological investigations in Mexico*, en *Smithsonian Misc Coll Smithsonian Institution,* 1951, 115, 475.

González García, F. y H. Gómez de Silva, *Especies endémicas: riqueza, patrones de distribución y retos para su conservación*, en Gómez, H. y A. Oliveras, editores, *Conservación de aves. Experiencias en México,* México, Sección Mexicana del Consejo Internacional para la Preservación de las Aves, (CIPAMEX), 2003.

Gutiérrez Contreras; A. T. Chávez Bárcenas; H. Guillén-Andrade, y M. B. Lara Chávez, *Agroecología de la franja aguacatera en Michoacán, México*, *Interciencia,* 2010, 35(9), 647-653.

Hernández Zamudio, M. A., *Caracterización y diagnóstico de la avifauna del municipio de Tancítaro en el marco del Ordenamiento Ecológico del Territorio: propuesta para la conservación,* Tesis de Licenciatura, Morelia, Facultad de Biología, Universidad Michoacana de San Nicolás de Hidalgo, 2011.

INEGI, X Censo General de Población y Vivienda. Integración Territorial, 2000.

INEGI, Información de los municipios, 2005.

INEGI, Anuario Estadístico de Michoacán, 2008.

Méndez, G. F.; J. F. Rodríguez S. y L. E. Villaseñor G., *Aves de verano en Michoacán*, en *Boletín No. 10 de la Coordinación de la Investigación Científica de la UMSNH,* 1987, 145-169.

Navia, J., y J. Barsimantov, *Cambio de uso del suelo: entre la legalidad y la impunidad*, en VIII Congreso Mexicano de Recursos Forestales Morelia Michoacán, México, 2007.

Noble, I. R. y Dirzo, *Forest as human dominated ecosystem*, en *Science,* 1997, 277(5325), 522-525.

Norma Oficial Mexicana Nom-059-Semarnat-2010, Protección ambiental-Especies nativas de México de flora y fauna silvestres-Categorías de riesgo y especificaciones para su inclusión, exclusión o cambio-Lista de especies en riesgo, en *Diario Oficial de la Federación,* diciembre de 2010.

Núñez Garduño, A., *Mamíferos*, en Villaseñor G. L. E., editora, *La biodiversidad en Michoacán: Estudio de Estado,* México, Comisión Nacional y Uso de la Biodiversidad, Secretaría de Urbanismo y Medio Ambiente, Universidad Michoacana de San Nicolás de Hidalgo, 2005.

Lugo, H. J., *Elementos de geomorfología aplicada (métodos cartográficos)*, Instituto de Geografía, UNAM, 1988.

OET, *Informe final del Ordenamiento Ecológico Territorial (OET) del municipio Nuevo Parangaricutiro, Michoacán*, Morelia, Universidad Michoacana de San Nicolás de Hidalgo, Secretaría de Urbanismo y Medio Ambiente, 2009, 288.

Ordoñez, D., y O. Flores Villela, *Áreas Naturales Protegidas,* PRONATURA y Conservación Internacional México, 1995, 43 (Serie Cuadernos de Conservación No. 4).

Palacio, P., *Cartografía geomorfológica a escala 1:50,000,* Instituto de Geografía, UNAM, 1986.

Paré L., M. A. González y D. Robinson, coords., *Gestión de cuencas y servicios ambientales perspectivas comunitarias y ciudadanas,* México, Secretaría de Medio Ambiente y Recursos Naturales, Instituto Nacional de Ecología, Itaca, Raises, Sendas, WWF, 2008.

Programa de Ordenamiento Ecológico Regional de la Cuenca del Río Tepalcatepec, del Estado de Michoacán de Ocampo, "Catálogo Electrónico de la legislación del Estado de Michoacán", en *Periódico Oficial del Estado,* 5 de junio de 2007.

Rzedowski, J., *La vegetación de México*, México, Limusa, 1978, 432.

Salas, P. M. A., *Aves de la Sierra Purépecha, estado de Michoacán,* Tesis de Licenciatura, México, Facultad de Ciencias de la UNAM, 1986, 79.

Scattolin M., *Studio geologico e morfometrico del settore centro occidentale Della MesetaTarasca, Michoacan, Messico,* Tesi di Laurea, Milán, Università degli Studi di Milano, Universidad Michoacana de San Nicolás Hidalgo, 1996, 136.

SIAP-Servicio de Información y Estadística Agroalimentaria y Pesquera, SIAP SAGARPA, *Avance siembras y cosechas perennes 2006,* SIAP-SAGARPA, 2006.

Torres, A. y G. Bocco, *Cambio de uso del suelo por cultivo de aguacate en la Meseta Tarasca, Michoacán, para los años de 1970 y 1990/92*, en Bocco, G. y M. Mendoza, *Evaluación de los cambios de la cobertura vegetal y uso del suelo en Michoacán (1975-1995). Los lineamientos para la ordenación ecológica de su territorio,* Informe Técnico, Morelia, Programa SIMORELOS-CONACYT, Departamento de Ecología de los Recursos Naturales, Instituto de Ecología, UNAM, 1999, 50 (más anexos).

Universidad Michoacana de San Nicolás de Hidalgo, Secretaría de Urbanismo y Medio Ambiente, *Informe final del Ordenamiento Ecológico Territorial del Municipio Nuevo Parangaricutiro, Michoacán,* Morelia, UMSNH, SUMA, 2009, 288.

Universidad Michoacana de San Nicolás de Hidalgo, Secretaría de Urbanismo y Medio Ambiente, *Informe final del Ordenamiento Ecológico Territorial del Municipio de Uruapan del Progreso, Michoacán,* Morelia, UMSNH, SUMA, 2010, 463.

Velázquez, A.; G. Bocco y A. Torres, *Turning scientific approaches into practical conservation actions: the case of Comunidad Indigena de Nuevo San Juan Parangaricutiro, México*, en *Environmental Management,* 2001, 5, 216-231.

Velázquez, A.; G. Bocco y A. Torres, *Las enseñanzas de San Juan. Investigación participativa para el manejo integral de recursos naturales,* México, INE-Semarnat, 2003.

Velázquez, A., *Biodiversidad, hábitat y manejo en el Parque Nacional Pico de Tancítaro, Michoacán,* Informe Final del proyecto R092, México, Conabio, Instituto de Geografía, UNAM, 2004.

Villa, B. y F. A. Cervantes, *Los mamíferos de México,* México, Grupo Editorial Iberoamérica, 2003.

Villalón, C. R. M.; H. Benítez, J. F. Villaseñor G.; L. E. Villaseñor G. y G. Chávez L., en Arizmendi, M. C. y L. Márquez Valdelamar, *Áreas de importancia para la conservación de las aves,* México, Conabio, 2000, 440.

Villalón, C. R. M., *Análisis altitudinal de la avifauna del transecto Tancítaro-Parácuaro, México,* Tesis de Licenciatura, Morelia, Escuela de Biología, UMSNH, 1990, 41 (más cuadros y figuras).

Villaseñor, G. L. E. y J. F. Villaseñor G., *Informe final del proyecto Análisis del estado actual de la avifauna del Parque Nacional Pico de Tancítaro, Michoacán*, Clave Convenio UMSNH/ SEP-DGICSA C90-01-0477 1991, 1992.

Villaseñor, G. L. E., editora, *La biodiversidad de Michoacán: Estudio de Estado,* México, Conabio, SUMA, UMSNH, 2005, 266.

Villaseñor, G. L. E., *Recuadro 6.2. La riqueza de vertebrados terrestres en las Áreas Naturales Protegidas federales. Anexo 6.3. Listado de las especies de anfibios registradas para las Áreas Naturales Protegidas federales de Michoacán*, en Villaseñor, G. L. E. *La biodiversidad de Michoacán: Estudio de Estado,* México, Conabio, SUMA, UMSNH, Morevallado Editores, 2005, 186 (disco compacto).

Villaseñor, G. L. E. y J. F. Villaseñor G., *Base de datos de las aves de Michoacán,* Laboratorio de Investigación en Ornitología, Facultad de Biología, Universidad Michoacana de San Nicolás de Hidalgo, 2011 (Formato Biotica 4.5).

Vitousek, P. M.; A. Harold y H. A. Mooney, *Human domination of Earth's ecosystems*, en *Science,* 1997, 277(5325), 494-499.

Zamora, E. V., *Inventario de hongos silvestres comestibles de la comunidad. "El Aguacate Sur" municipio de Tancítaro, Michoacán,* Tesis de Licenciatura, México, Facultad de Biología,UMSNH, 2006.

11. ANEXOS

Anexo I

Listados florísticos y faunísticos

Listado de flora

Orden	Familia	Nombre científico	Nombre común	**Categoría de riesgo
**Apiales**	Apiaceae	*Apium leptophyllum*		
	Apiaceae	*Arracacia tolucensis*		
	Apiaceae	*Arracacia atropurpurea*		
	Apiaceae	*Arracacia aegopodioides*		
	Apiaceae	*Arracacia rigida*		
	Apiaceae	*Daucus montanus*		
	Araliaceae	*Dendropanax arboreus*		
	Apiaceae	*Donnellsmithia mexicana*		
	Apiaceae	*Eryngium carlinae*		
	Apiaceae	*Eryngium ghiesbreghtii*		
	Apiaceae	*Eryngium beecheyanum*		
	Apiaceae	*Eryngium alternatum*		
	Apiaceae	*Eryngium mexiae*		
	Apiaceae	*Oreomyrrhis tolucana*		
	Araliaceae	Oreopanax echinops		
	Araliaceae	*Oreopanax xalapensis*		
	Apiaceae	*Rhodosciadium tolucense*		
	Apiaceae	*Rhodosciadium purpureum*		
	Apiaceae	*Tauschia decumbens*		
**Asterales**	Asteraceae	*Ageratum corymbosum*		
	Asteraceae	*Archibaccharis schiedeana*		

Orden	Familia	Nombre científico	Nombre común	**Categoría de riesgo
	Asteraceae	*Archibaccharis hieraciifolia*		
	Asteraceae	*Archibaccharis asperifolia*		
	Asteraceae	*Aster moranensis*		
	Asteraceae	Astranthium orthopodum		
	Asteraceae	*Baccharis heterophylla*		
	Asteraceae	*Baccharis multiflora herbacea*		
	Asteraceae	*Baccharis pteronioides*		
	Asteraceae	*Bidens aequisquama*		
	Asteraceae	*Bidens ostruthioides*		
	Asteraceae	*Bidens triplinervia*		
	Asteraceae	*Bidens pilosa*		
	Asteraceae	*Brickellia secundiflora monticola*		
	Asteraceae	*Brickellia pedunculosa*		
	Asteraceae	Brickellia squarrosa oligadena		
	Asteraceae	*Calea scabra*		
	Asteraceae	*Calea colimensis*		
	Asteraceae	*Calea integrifolia*		
	Asteraceae	*Cirsium nivale*		
	Asteraceae	*Cirsium anartiolepis*		
	Asteraceae	*Cirsium tolucanum*		
	Asteraceae	*Cirsium ehrenbergii*		
	Asteraceae	*Conyza canadensis*		
	Asteraceae	*Conyza coronopifolia*		
	Asteraceae	*Dahlia scapigera*	dalia	Pr
	Asteraceae	*Erigeron galeottii*		

Orden	Familia	Nombre científico	Nombre común	**Categoría de riesgo
	Asteraceae	*Erigeron polycephalus*		
	Asteraceae	*Erigeron karvinskianus*		
	Asteraceae	*Erigeron velutipes*		
	Asteraceae	*Eupatorium glabratum*		
	Asteraceae	*Eupatorium rivale*		
	Asteraceae	*Eupatorium oresbium*		
	Asteraceae	*Eupatorium dolichobasis*		
	Asteraceae	*Eupatorium mairetianum*		
	Asteraceae	Eupatorium patzcuarense		
	Asteraceae	*Eupatorium pycnocephalum*		
	Asteraceae	*Gnaphalium americanum*		
	Asteraceae	*Gnaphalium liebmannii monticola*		
	Asteraceae	*Gnaphalium oxyphyllum*		
	Asteraceae	*Gnaphalium bourgovii*		
	Asteraceae	*Gnaphalium roseum*		
	Asteraceae	*Gnaphalium attenuatum*		
	Asteraceae	*Gnaphalium salicifolium*		
	Asteraceae	*Gnaphalium viscosum*		
	Asteraceae	*Heterotheca inuloides rosei*		
	Asteraceae	*Hieracium abscissum*		
	Asteraceae	*Hieracium schultzii*		
	Asteraceae	*Jaegeria macrocephala*		
	Asteraceae	*Lactuca serriola*		
	Asteraceae	*Montanoa frutescens*		
	Asteraceae	*Pericalia michoacana*		

Orden	Familia	Nombre científico	Nombre común	**Categoría de riesgo
	Asteraceae	*Perymenium alticola*		
	Asteraceae	*Perymenium buphtalmoides flexuosum*		
	Asteraceae	*Pinaropappus roseus*		
	Asteraceae	*Piqueria triflora*		
	Asteraceae	*Piqueria pilosa*		
	Asteraceae	*Piqueria trinervia*		
	Asteraceae	*Podachaenium eminens*		
	Asteraceae	*Psacalium megaphyllum*		
	Asteraceae	*Rumfordia floribunda*		
	Asteraceae	*Sabazia humilis*		
	Asteraceae	*Sabazia liebmanii*		
	Asteraceae	*Senecio sanguisorbae*		
	Asteraceae	*Senecio callosus*		
	Asteraceae	*Senecio stoechadiformis*		
	Asteraceae	*Senecio toluccanus*		
	Asteraceae	*Senecio salignus*		
	Asteraceae	*Senecio calcarius*		
	Asteraceae	*Senecio albonervius*		
	Asteraceae	*Senecio angulifolius*		
	Asteraceae	*Senecio barba-johannis*		
	Asteraceae	*Sigesbeckia jorullensis*		
	Asteraceae	*Sonchus oleraceus*		
	Asteraceae	*Stevia monardifolia*		
	Asteraceae	Stevia nelsonii		
	Asteraceae	*Stevia lucida*		

Orden	Familia	Nombre científico	Nombre común	**Categoría de riesgo
	Asteraceae	*Tagetes remotiflora*		
	Asteraceae	*Tagetes filifolia*	pericón	
	Asteraceae	*Taraxacum officinale*		
	Asteraceae	*Tithonia tubaeformis*		
	Asteraceae	*Trigonospermum melampodioides*		
	Asteraceae	*Verbesina discoidea*		
	Asteraceae	*Verbesina oncophora oncophora*		
	Asteraceae	*Verbesina klattii*		
	Asteraceae	*Verbesina greenmanii*		
	Asteraceae	*Vernonia alamanii dictyophlebia*		
**Brassicales**	Brassicaceae	*Rorippa* sp.		
**Bromeliales**	Bromeliaceae	*Pitcairnia karwinskiana*		
	Bromeliaceae	*Tillandsia prodigiosa*		
	Bromeliaceae	*Tillandsia macdougalli*		
**Capparales**	Brassicaceae	*Brassica campestris*		
	Brassicaceae	*Cardamine flaccida*		
	Brassicaceae	*Draba jorullensis*		
	Brassicaceae	*Halimolobos berlandieri*		
	Brassicaceae	*Lepidium virginicum*		
	Brassicaceae	*Raphanus raphanistrum*		
	Brassicaceae	*Romanschulzia arabiformis*		
**Caryophyllales**	Amaranthaceae	*Amaranthus hybridus*		
	Caryophyllaceae	*Arenaria reptans*		
	Caryophyllaceae	*Arenaria lanuginosa*		
	Caryophyllaceae	*Arenaria bourgaei*		

Orden	Familia	Nombre científico	Nombre común	**Categoría de riesgo
	Caryophyllaceae	*Arenaria oresbia*		
	Caryophyllaceae	*Cerastium molle*		
	Caryophyllaceae	*Cerastium nutans*		
	Chenopodiaceae	*Chenopodium ambrosioides*		
	Chenopodiaceae	*Chenopodium album*		
	Portulacaceae	*Claytonia perfoliata*		
	Caryophyllaceae	*Drymaria cordata*		
	Cactaceae	*Heliocereus elegantissimus*		
	Amaranthaceae	*Iresine diffusa*		
	Phytolaccaceae	*Phytolacca icosandra*		
	Caryophyllaceae	*Stellaria media*		
	Caryophyllaceae	*Stellaria cuspidata*		
**Celastrales**	Celastraceae	*Celastrus pringlei*		
	Aquifoliaceae	*Ilex tolucana*		
**Commelinales**	Commelinaceae	*Commelina tuberosa*		
	Commelinaceae	*Gibasis pulchella*		
	Commelinaceae	*Tinantia violacea*		
	Commelinaceae	*Tinantia erecta*		
	Pontederiaceae	*Heteranthera limosa*		
**Coniferales**	Pinaceae	*Abies religiosa*		
	Cupressaceae	*Cupressus lusitanica*	cedro blanco	Pr
	Cupressaceae	*Juniperus monticola*	enebro azul	Pr
	Pinaceae	*Pinus hartwegii*		
	Pinaceae	*Pinus teocote*		
	Pinaceae	*Pinus leiophylla*		

**Orden**	**Familia**	**Nombre científico**	**Nombre común**	****Categoría de riesgo**
	Pinaceae	*Pinus douglasiana*		
	Pinaceae	*Pinus maximinoi*		
	Pinaceae	*Pinus devoniana*		
	Pinaceae	*Pinus pseudostrobus pseudostrobus*		
	Pinaceae	*Pinus montezumae*		
**Cornales**	Cornaceae	*Cornus disciflora*		
	Cornaceae	*Cornus excelsa*		
	Garryaceae	*Garrya longifolia*		
	Garryaceae	*Garrya laurifolia*		
**Cyperales**	Poaceae	*Aegopogon cenchroides*	zacate barbón	
	Poaceae	*Avena fatua*		
	Poaceae	*Bouteloua purpurea*		
	Poaceae	*Brachypodium mexicanum*		
	Poaceae	*Bromus exaltatus*	zacate	
	Poaceae	*Calamagrostis valida*		
	Cyperaceae	*Carex turbinata*		
	Cyperaceae	*Carex peucophila*		
	Cyperaceae	*Carex* aff. *Tuberculata*		
	Poaceae	*Chusquea* aff. *Circinata*		
	Poaceae	*Cynodon dactylon*		
	Cyperaceae	*Cyperus hermaphroditus*		
	Cyperaceae	*Cyperus arsenei*		
	Cyperaceae	*Cyperus spectabilis*		
	Cyperaceae	*Cyperus michoacanensis*		
	Cyperaceae	*Cyperus manimae*		

**Orden**	**Familia**	**Nombre científico**	**Nombre común**	****Categoría de riesgo**
	Poaceae	*Festuca amplissima*	pasto	
	Poaceae	*Festuca breviglumis*	pasto	
	Cyperaceae	*Kyllinga odorata*		
	Poaceae	*Muhlenbergia macroura*		
	Poaceae	*Muhlenbergia quadridentata*		
	Poaceae	*Piptochaetium virescens*		
	Poaceae	*Poa annua*	zacate azul	
	Poaceae	*Sporobolus indicus*		
	Poaceae	*Trisetum virletii*	zacate	
	Poaceae	*Zeugites americana pringlei*		
**Dipsacales**	Caprifoliaceae	*Lonicera japonica*		
	Caprifoliaceae	*Sambucus mexicana*		
	Caprifoliaceae	*Symphoricarpos microphyllus*		
	Valerianaceae	*Valeriana robertianifolia*		
	Valerianaceae	*Valeriana urticaefolia*		
	Valerianaceae	*Valeriana clematitis*		
	Caprifoliaceae	*Viburnum lautum*		
	Caprifoliaceae	*Viburnum elatum*		
	Caprifoliaceae	*Viburnum hartwegii*		
	Caprifoliaceae	*Viburnum discolor*		
**Ebenales**	Styracaceae	*Styrax argenteus ramirezii*		
	Symplocaceae	*Symplocos citrea*		
**Equisetales**	Equisetaceae	*Equisetum hyemale affine*		
**Ericales**	Ericaceae	*Arbutus occidentalis*	madroño	Pr
	Ericaceae	*Arbutus xalapensis*		

Orden	Familia	Nombre científico	Nombre común	**Categoría de riesgo
	Clethraceae	*Clethra hartwegii*		
	Clethraceae	*Clethra mexicana*	jaboncillo,	
	Ericaceae	*Comarostaphylis discolor*	madroño borracho	Pr
	Ericaceae	*Gaultheria lancifolia*		
	Monotropaceae	*Monotropa uniflora*		
	Monotropaceae	*Monotropa hypopitys*	pipa de indio	Pr
	Ericaceae	*Pernettya ciliata*		
	Ericaceae	*Vaccinium geminiflorum*		
**Euphorbiales**	Euphorbiaceae	*Acalypha subviscida*		
	Euphorbiaceae	*Acalypha mollis*		
	Euphorbiaceae	*Euphorbia sphaerorhiza*		
	Euphorbiaceae	*Euphorbia radians stormiae*		
	Euphorbiaceae	*Euphorbia postrata*		
	Euphorbiaceae	*Euphorbia hirta nocens*		
	Euphorbiaceae	*Euphorbia macropus*		
	Euphorbiaceae	*Euphorbia ocymoides*		
	Euphorbiaceae	*Ricinus communis*		
**Fabales**	Mimosaceae	*Acacia angustissima*		
	Mimosaceae	*Acacia farnesiana*		
	Fabaceae	*Astragalus guatemalensis brevidentatus*		
	Mimosaceae	*Calliandra anomala*		
	Mimosaceae	*Calliandra grandiflora*		
	Fabaceae	*Calopogonium mucunoides*		
	Fabaceae	*Cologania broussonetii*		
	Fabaceae	*Cologania* aff. *Ovobata*		

Orden	Familia	Nombre científico	Nombre común	**Categoría de riesgo
	Fabaceae	*Crotalaria longirostrata*		
	Fabaceae	*Crotalaria rotundifolia vulgaris*		
	Fabaceae	*Crotalaria polyphylla*		
	Fabaceae	*Dalea leucostachys eysenhardtioides*		
	Fabaceae	*Dalea obreniformis*		
	Fabaceae	*Dalea dipsacea*		
	Fabaceae	*Desmodium sumichrastii*		
	Fabaceae	*Desmodium grahamii*		
	Fabaceae	*Desmodium novogalicianum*		
	Fabaceae	*Desmodium cordistipulum cryptopodum*		
	Fabaceae	*Desmodium densiflorum*		
	Fabaceae	*Erythrina flabelliformis*		
	Fabaceae	*Lotus repens*		
	Fabaceae	*Lupinus elegans*		
	Fabaceae	*Lupinus montanus*		
	Fabaceae	*Lupinus exaltatus*		
	Fabaceae	*Lupinus aschenbornii*		
	Fabaceae	*Lupinus reflexus*		
	Fabaceae	*Lupinus campestris*		
	Fabaceae	*Macroptilium atropurpureum*		
	Fabaceae	*Macroptilium gibbosifolium*		
	Mimosaceae	*Mimosa galeottii*		
	Fabaceae	*Phaseolus coccineus*		
	Fabaceae	*Phaseolus polymorphus*		
	Fabaceae	*Rhynchosia discolor*		

Orden	Familia	Nombre científico	Nombre común	**Categoría de riesgo
	Caesalpiniaceae	*Senna multiglandulosa*		
	Caesalpiniaceae	*Senna pallida pallida*		
	Fabaceae	*Trifolium amabile*		
	Fabaceae	*Vicia humilis*		
	Fabaceae	*Zornia reticulata*		
**Fagales**	Betulaceae	*Alnus acuminata arguta*		
	Betulaceae	*Alnus jorullensis jorullensis*		
	Betulaceae	*Carpinus tropicalis*		
	Fagaceae	*Quercus laurina*		
	Fagaceae	*Quercus rugosa*		
	Fagaceae	*Quercus crassipes*		
	Fagaceae	*Quercus martinezii*		
	Fagaceae	*Quercus candicans*		
	Fagaceae	*Quercus obtusata*		
**Filicales**	Pteridaceae	*Adiantum andicola*		
	Aspleaniaceae	*Asplenium monanthes*		
	Aspleaniaceae	*Asplenium munchii*		
	Blechnaceae	*Blechnum polypodioides*		
	Polypodiaceae	*Campyloneurum angustifolium*		
	Woodsiaceae	*Cystopteris fragilis*		
	Dryopteridaceae	*Dryopteris wallichiana*		
	Lomariopsidaceae	*Elaphoglossum erinaceum*		
	Lomariopsidaceae	*Elaphoglossum petiolatum*		
	Lomariopsidaceae	*Elaphoglossum glaucum*		
	Dryopteridaceae	*Plecosorus speciosissimum*		

Orden	Familia	Nombre científico	Nombre común	**Categoría de riesgo
	Polypodiaceae	*Pleopeltis mexicana*		
	Polypodiaceae	*Polypodium madrense*		
	Polypodiaceae	*Polypodium subpetiolatum*		
	Polypodiaceae	*Polypodium platylepis*		
	Pteridaceae	*Pteris cretica*		
	Blechnaceae	*Woodwardia spinulosa*		
**Gentianales**	Asclepiadaceae	*Asclepias notha*		
	Loganiaceae	*Buddleja parviflora*		
	Loganiaceae	*Buddleja cordata*		
	Loganiaceae	*Buddleja sessiliflora*		
	Gentianaceae	*Gentiana spathacea*	flor de hielo	Pr
	Gentianaceae	*Halenia plantaginea*		
	Gentianaceae	*Halenia crassuiscula*		
	Gentianaceae	*Halenia brevicornis*		
**Geraniales**	Geraniaceae	*Geranium seemannii*		
	Oxalidaceae	*Oxalis alpina*		
	Oxalidaceae	*Oxalis albicans*		
	Oxalidaceae	*Oxalis corniculata*		
	Oxalidaceae	*Oxalis latifolia*		
	Oxalidaceae	*Oxalis hernandesii*		
**Juncales**	Juncaceae	*Luzula racemosa*		
	Juncaceae	*Luzula gigantea*		
	Juncaceae	*Luzula caricina*		
**Lamiales**	Verbenaceae	*Citharexylum affine*		
	Boraginaceae	*Hackelia mexicana*		

Orden	Familia	Nombre científico	Nombre común	**Categoría de riesgo
	Lamiaceae	*Lepechinia caulescens*		
	Verbenaceae	*Lippia umbellata*		
	Boraginaceae	*Macromeria longiflora*		
	Lamiaceae	*Prunella vulgaris*		
	Lamiaceae	*Salvia elegans*		
	Lamiaceae	*Salvia fulgens*		
	Lamiaceae	*Salvia vasquezii*		
	Lamiaceae	*Salvia mexicana*		
	Lamiaceae	*Salvia iodantha*		
	Lamiaceae	*Salvia clinopodioides*		
	Lamiaceae	*Salvia mocinnoi*		
	Lamiaceae	*Salvia thyrsiflora*		
	Lamiaceae	*Salvia lavanduloides*		
	Lamiaceae	*Salvia microphylla*		
	Lamiaceae	*Salvia gracilis*		
	Lamiaceae	*Salvia albocaerulea*		
	Lamiaceae	*Satureja macrostema*	nurite	
	Lamiaceae	*Scutellaria caerulea*		
	Lamiaceae	*Stachys coccinea*		
	Lamiaceae	*Stachys radicans*		
	Lamiaceae	*Stachys eriantha*		
	Lamiaceae	*Stachys nepetifolia*		
	Lamiaceae	*Stachys keerlii*		
	Boraginaceae	*Tournefortia glabra*		
	Verbenaceae	*Verbena carolina*		

Orden	Familia	Nombre científico	Nombre común	**Categoría de riesgo
	Verbenaceae	*Verbena teucriifolia*		
**Laurales**	Lauraceae	*Cinnamomum hartmanii*		
**Liliales**	Agavaceae	*Agave inaequidens*		
	Liliaceae	*Bomárea hirtella*		
	Dioscoreaceae	*Dioscorea minima*		
	Dioscoreaceae	*Dioscorea* aff. *Galeottiana*		
	Liliaceae	*Echeandia mexicana*		
	Liliaceae	*Echeandia durangensis*		
	Agavaceae	*Furcraea bedinghausii*	palmita	A*
	Liliaceae	*Hypoxis mexicana*		
	Liliaceae	*Milla biflora*		
	Iridaceae	*Sisyrinchium convolutum*		
	Iridaceae	*Sisyrinchium palmeri*		
	Iridaceae	*Sisyrinchium schaffneri*		
	Iridaceae	*Sisyrinchium pringlei*		
	Smilacaceae	*Smilax moranensis*		
	Smilacaceae	*Smilax pringlei*		
	Liliaceae	*Stenanthium frigidum*		
	Iridaceae	*Tigridia alpestris obtusa*		
	Liliaceae	*Zephyranthes fosteri*		
**Lycopodiales**	Lycopodiaceae	*Lycopodium pringlei*		
**Magnoliales**	Annonaceae	*Annona cherimola*		
**Malvales**	Malvaceae	*Abutilon ellipticum*		
	Malvaceae	*Anoda cristata*		
	Malvaceae	*Kearnemalvastrum subtriflorum*		

Orden	Familia	Nombre científico	Nombre común	**Categoría de riesgo
	Malvaceae	*Malva parviflora*		
	Malvaceae	*Malvaviscus arboreus*		
	Malvaceae	*Neobrittonia acerifolia*		
	Malvaceae	*Phymosia rosea*	vara de San Juan	Pr
	Malvaceae	*Sida rhombifolia*		
	Tiliaceae	*Tilia mexicana*	flor de tila	
**Myrtales**	Lythraceae	*Cuphea bustamanta*		
	Onagraceae	*Epilobium ciliatum*		
	Onagraceae	*Fuchsia microphylla microphylla*		
	Onagraceae	*Fuchsia thymifolia thymifolia*		
	Onagraceae	*Fuchsia arborescens*		
	Onagraceae	*Fuchsia fulgensi*		
	Onagraceae	*Fuchsia obconica*		
	Onagraceae	*Gaura hexandra*		
	Onagraceae	*Lopezia racemosa racemosa*		
	Onagraceae	*Lopezia miniata*		
	Onagraceae	*Lopezia trichota*		
	Onagraceae	*Oenothera rosea*		
	Onagraceae	*Oenothera pubescens*		
**Najadales**	Potamogetonaceae	*Potamogeton diversifolius*		
**Ophioglossales**	Ophioglossaceae	*Ophioglossum crotalophoroides*		
**Orchidales**	Orchidaceae	*Cymbiglossum cervantesii*		
	Orchidaceae	*Encyclia venosa*		
	Orchidaceae	*Epidendrum anisatum*		
	Orchidaceae	*Epidendrum longipetalum*		

Orden	Familia	Nombre científico	Nombre común	**Categoría de riesgo
	Orchidaceae	*Erycina diaphana*		
	Orchidaceae	*Govenia superba*		
	Orchidaceae	*Govenia purpusii*		
	Orchidaceae	*Homalopetalum pachyphyllum*		
	Orchidaceae	*Isochilus amparoanus*		
	Orchidaceae	*Liparis draculoides*		
	Orchidaceae	*Malaxis ehrenbergii*		
	Orchidaceae	*Maxillaria* aff. *Variabilis*		
	Orchidaceae	*Oncidium reichenheimii*		
	Orchidaceae	*Pleurothallis* aff. *Quadrifida*		
	Orchidaceae	*Pleurothallis hirsuta*		
	Orchidaceae	*Pleurothallis longispicata*		
	Orchidaceae	*Pleurothallis hieroglyphica*		
	Orchidaceae	*Spiranthes hyemalis*		
	Orchidaceae	*Spiranthes eriophora*		
	Orchidaceae	*Spiranthes rubrocalosa*		
	Orchidaceae	*Spiranthes pyramidalis*		
**Papaverales**	Papaveraceae	*Argemone platyceras*		
	Papaveraceae	*Argemone ochroleuca ochroleuca*		
	Papaveraceae	*Bocconia arborea*		
	Papaveraceae	*Bocconia frutescens*		
**Piperales**	Piperaceae	*Peperomia galioides*		
	Piperaceae	*Peperomia hintonii*		
	Piperaceae	*Peperomia campylotropa*		
	Piperaceae	*Peperomia hispidula*		

Orden	Familia	Nombre científico	Nombre común	**Categoría de riesgo
	Piperaceae	*Peperomia quadrifolia*		
	Piperaceae	*Piper amalago*		
**Plantaginales**	Plantaginaceae	*Plantago linearis villosa*		
	Plantaginaceae	*Plantago australis*		
**Polygalales**	Malpighiaceae	*Galphimia glauca*		
	Malpighiaceae	*Gaudichaudia albida*		
	Polygalaceae	*Monnina xalapensis*		
	Polygalaceae	*Monnina xalapensis*		
	Polygalaceae	*Polygala aparinoides*		
	Polygonaceae	*Polygonum hydropiperoides*		
	Polygonaceae	*Rumex conglomeratus*		
**Primulales**	Primulaceae	*Anagallis arvensis*		
	Myrsinaceae	*Parathesis villosa*		
	Myrsinaceae	*Rapanea juergensenii*		
**Ranunculales**	Berberidaceae	*Berberis moranensis*		
	Ranunculaceae	*Clematis dioica*		
	Coriariaceae	*Coriaria ruscifolia microphylla*	helecho de tierra, borrego, hierba de borrego	
	Sabiaceae	*Meliosma dentata*		
	Ranunculaceae	*Ranunculus petiolaris trahens*		
	Ranunculaceae	*Ranunculus donianus*		
	Ranunculaceae	*Thalictrum pubigerum*		
**Rhamnales**	Rhamnaceae	*Ceanothus coeruleus*		
	Vitaceae	*Vitis tilifolia*		
**Rosales**	Rosaceae	*Acaena elongata*	abrojo, pega ropa	

Orden	Familia	Nombre científico	Nombre común	**Categoría de riesgo
	Rosaceae	*Alchemilla*		
	Rosaceae	*Alchemilla pringlei*		
	Rosaceae	*Alchemilla vulcanica*		
	Rosaceae	*Alchemilla pringlei*		
	Rosaceae	*Alchemilla pectinata*		
	Rosaceae	*Crataegus pubescens*	tejocote	
	Crassulaceae	*Echeveria fulgens*		
	Saxifragaceae	*Heuchera orizabensis*		
	Rosaceae	*Holodiscus fissus*		
	Hydrangeaceae	*Philadelphus mexicanus*		
	Rosaceae	*Prunus brachybotrya*		
	Rosaceae	*Prunus serotina capuli*		
	Grossulariaceae	*Ribes ciliatum*		
	Rosaceae	*Rubus liebmannii*		
	Crassulaceae	*Sedum tortuosum*		
	Crassulaceae	*Sedum neovolcanicum*		
	Crassulaceae	*Villadia batesii*		
**Rubiales**	Rubiaceae	*Borreria laevis*		
	Rubiaceae	*Borreria verticillata*		
	Rubiaceae	*Crusea coccinea*		
	Rubiaceae	*Crusea longiflora*		
	Rubiaceae	*Didymaea alsinioides*		
	Rubiaceae	*Didymaea floribunda*		
	Rubiaceae	*Galium mexicanum*		
	Rubiaceae	*Galium aschenbornii*		

Orden	Familia	Nombre científico	Nombre común	**Categoría de riesgo
	Rubiaceae	*Hedyotis cervantesii*		
**Salicales**	Salicaceae	*Salix paradoxa*		
**Santalales**	Viscaceae	*Arceuthobium globosum*		
	Loranthaceae	*Cladocolea microphylla*		
	Loranthaceae	*Cladocolea pedicellata*		
	Loranthaceae	*Cladocolea loniceroides*		
	Viscaceae	*Phoradendron falcatum*		
	Viscaceae	*Phoradendron velutinum*		
	Loranthaceae	*Psittacanthus macrantherus*		
	Loranthaceae	*Struthanthus microphyllus*		
	Loranthaceae	*Struthanthus condensatus*		
**Sapindales**	Anacardiaceae	*Rhus radicans*		
**Scrophulariales**	Scrophulariaceae	*Agalinis peduncularis*		
	Scrophulariaceae	*Calceolaria mexicana*		
	Scrophulariaceae	*Castilleja scarzoneraefolia*		
	Scrophulariaceae	*Castilleja arvensis*		
	Scrophulariaceae	*Castilleja tenuiflora*		
	Orobanchaceae	*Conopholis alpina*		
	Oleaceae	*Fraxinus uhdei*		
	Scrophulariaceae	*Lamourouxia multifida*		
	Scrophulariaceae	*Mimulus glabratus*		
	Scrophulariaceae	*Penstemon roseus*		
	Scrophulariaceae	*Penstemon campanulatus*		
	Lentibulariaceae	*Pinguicula oblongiloba*		
	Lentibulariaceae	*Pinguicula moranensis*		

Orden	Familia	Nombre científico	Nombre común	**Categoría de riesgo
	Scrophulariaceae	*Russelia sarmentosa*		
	Scrophulariaceae	*Sibthorpia repens*		
	Scrophulariaceae	*Veronica peregrina xalapensis*		
**Solanales**	Polemoniaceae	*Bonplandia geminiflora*		
	Solanaceae	*Cestrum nitidum*		
	Solanaceae	*Cestrum anagyris*		
	Solanaceae	*Cestrum thyrsoideum*		
	Solanaceae	*Cestrum lanatum*		
	Convolvulaceae	*Cuscuta obtusiflora*		
	Convolvulaceae	*Cuscuta mitraeformis*		
	Convolvulaceae	*Cuscuta rugosiceps grandiflora*		
	Convolvulaceae	*Cuscuta corymbosa*		
	Solanaceae	*Datura stramonium*		
	Convolvulaceae	*Ipomoea orizabensis*		
	Convolvulaceae	*Ipomoea tancitarense*		
	Solanaceae	*Jaltomata procumbens*		
	Polemoniaceae	*Loeselia mexicana*		
	Polemoniaceae	*Loeselia glandulosa*		
	Hydrophyllaceae	*Nama dichotomum*		
	Hydrophyllaceae	*Phacelia platycarpa*		
	Solanaceae	*Physalis orizabae*		
	Solanaceae	*Physalis stapelioides*		
	Solanaceae	*Physalis volubilis*		
	Solanaceae	*Physalis coztomatl*		
	Solanaceae	*Solandra nitida*		

Orden	Familia	Nombre científico	Nombre común	**Categoría de riesgo
	Solanaceae	*Solanum verrucosum*		
	Solanaceae	*Solanum appendiculatum*		
	Solanaceae	*Solanum stoloniferum*		
	Solanaceae	*Solanum brachystachys*		
	Solanaceae	*Solanum cervantesii*		
	Solanaceae	*Solanum nigrescens*		
	Solanaceae	*Solanum mozinianum*		
	Solanaceae	*Solanum lanceolatum*		
**Theales**	Theaceae	*Cleyera integrifolia*	aceituno	
	Clusiaceae	*Hypericum philonotis*		
	Actinidiaceae	*Saurauia serrata*	almasante, mameyito	Pr
	Theaceae	*Ternstroemia lineata*	tila roja	
**Urticales**	Moraceae	*Ficus goldmanii*		
	Urticaceae	*Parietaria pensylvanica*		
	Urticaceae	*Urtica urens*		
**Violales**	Begoniaceae	*Begonia gracilis*		
	Begoniaceae	*Begonia asteroides*		
	Cucurbitaceae	*Cyclanthera langaei*		
	Cucurbitaceae	*Cyclanthera integrifolia*		
	Cistaceae	*Helianthemum glomeratum*		
	Cistaceae	*Lechea tripetala*		
	Cucurbitaceae	*Microsechium helleri*		
	Cucurbitaceae	*Parasicyos* aff. *Tuberculata*		
	Passifloraceae	*Passiflora exudans*		
	Passifloraceae	*Passiflora pavonis*		

Orden	Familia	Nombre científico	Nombre común	**Categoría de riesgo
	Violaceae	*Viola ciliata*		
	Flacourtiaceae	*Xylosma flexuosum*		
**Campanulales**	Campanulaceae	*Diastatea micrantha*		
	Campanulaceae	*Lobelia laxiflora*		
	Campanulaceae	*Lobelia nana*		
	Campanulaceae	*Lobelia plebeia*		

Basado en: García Ruíz, I., *Flora del Parque Nacional Pico de Tancítaro, Michoacán,* México, Instituto Politécnico Nacional, Centro Interdisciplinario de Investigación para el Desarrollo Integral Regional-Michoacán, Bases de datos SNIB2010-Conabio proyecto No. H304, 1999, disponible en http://www.conabio.gob.mx.
**Categorías de riesgo de acuerdo a la Norma Oficial Mexicana Nom-059-Semarnat-2010, Protección ambiental-Especies nativas de México de flora y fauna silvestres-Categorías de riesgo y especificaciones para su inclusión, exclusión o cambio-Lista de especies en riesgo.
* Endémica (e).
A: amenazada; Pr: sujeta a protección especial.

Hongos

Familia	Nombre científico	Nombre común	Categoría de riesgo**
**Geoglossaceae**	*Trichoglossum hirsutum*		
**Discinaceae**	*Gyromitra esculenta*		
**Helvellaceae**	*Helvella acetabulum*		
	Helvella crispa	soldadito blanco o gachupín	
	Helvella cupuliformis		
	Helvella lacunosa		
	Helvella macropus		
**Morchellaceae**	*Morchella angusticeps*		A
	Morchella esculenta	mazorca, mazorquita	A
**Pezizaceae**	*Peziza badia*		
	Peziza badiofusca		

Familia	Nombre científico	Nombre común	Categoría de riesgo**
**Pyronemataceae**	*Aleuria aurantia*		
	Humaria hemisphaerica		
	Otidea alutacea		
	Otidea onotica		
	Scutellinia scutellata		
**Hypocreaceae**	*Hypomyces lactifluorum*	San Pedro, orejas de San Pedro, San Pedro blanco, San Pedro café, trompa de puerco, trompas	
**Xylariaceae**	*Daldinia concentrica*		
	Xylaria hypoxylon		
**Agaricaceae**	*Agaricus augustus*		A
	Agaricus placomyces		
	Agaricus xanthodermus		
	Macrolepiota procera	hongo de codorniz, hongo de culebra	
**Coprinaceae**	*Coprinus comatus*		
	Psathyrella candolleana		
**Cortinariaceae**	*Cortinarius collinitus*		
	Gymnopilus penetrans		
	Inocybe calamistrata		
	Inocybe geophylla var. *geophylla*		
	Inocybe geophylla var. *lilacina*		
**Entolomataceae**	*Entoloma clypeatum*		
**Hydnangiaceae**	*Laccaria amethystina*		
	Laccaria bicolor		
	Laccaria laccata	agritos, clavitos, moraditos, hongo de lima	
**Lycoperdaceae**	*Calvatia cyathiformis*		

Familia	Nombre científico	Nombre común	Categoría de riesgo**
	Lycoperdon perlatum		
	Lycoperdon umbrinum		
**Marasmiaceae**	*Armillaria mellea*		
	Marasmius cohaerens		
	Marasmius rotula		
	Marasmius spegazzinii		
**Pluteaceae**	*Amanita caesárea*		
	Amanita chlorinosma		
	Amanita calyptratoides	amarillo	
	Amanita fulva		
	Amanita umbrinolutea		
	Amanita gemmata		
	Amanita muscaria	hongo tecomate de moscas	A
	Amanita pantherina		
	Amanita rubescens		
	Amanita tuza		
	Amanita vaginata	plateados	
	Amanita virosa		
	Pluteus ephebeus		
**Schizophyllaceae**	*Schizophyllum commune*		
**Strophariaceae**	*Hypholoma fasciculare*		
	Pholiota squarrosa		
**Tricholomataceae**	*Clitocybe gibba*		
	Collybia polyphylla		
	Gymnopus acervatus		

Familia	Nombre científico	Nombre común	Categoría de riesgo**
	Gymnopus alkalivirens		
	Gymnopus confluens		
	Gymnopus dryophilus		
	Hygrocybe cantharellus		
	Hygrocybe conica		
	Hygrophorus russula	ririchaka (raramuri)	A
	Melanoleuca melaleuca		
	Mycena epipterygia		
	Mycena pura		
	Rhodocollybia butyracea		
	Tricholomopsis rutilans		
	Xeromphalina campanella		
**Boletaceae**	*Boletellus ananas*		
	Boletus chrysenteron		
	Boletus pinophilus		
	Buchwaldoboletus hemichrysus		
	Strobilomyces strobilaceus		
	Tylopilus felleus		
**Gomphidiaceae**	*Chroogomphus rutilus*		
**Gyroporaceae**	*Gyroporus castaneus*		
**Hygrophoropsidaceae**	*Hygrophoropsis aurantiaca*	Santa María, tejamanilero	
**Paxillaceae**	*Paxillus atrotomentosus*		
	Tapinella panuoides		
**Sclerodermataceae**	*Astraeus hygrometricus*		
	Scleroderma areolatum		

Familia	Nombre científico	Nombre común	Categoría de riesgo**
	Scleroderma polyrhizum		
**Suillaceae**	*Suillus cothurnatus*		
	Suillus tomentosus	pancitas	
**Cantharellaceae**	*Cantharellus cibarius*	rebozuelo	
**Clavulinaceae**	*Clavulina coralloides*		
**Dacrymycetaceae**	*Calocera cornea*		
	Dacrymyces palmatus		
**Hymenochaetaceae**	*Coltricia perennis*		
	Inonotus hispidus		
	Phellinus gilvus		
	Phellinus igniarius		
**Geastraceae**	*Geastrum triplex*		
**Gomphaceae**	*Gomphus floccosus*		
**Phallaceae**	*Clathrus ruber*		
**Ramariaceae**	*Ramaria stricta*		
**Fomitopsidaceae**	*Fomitopsis pinicola*		
**Ganodermataceae**	*Ganoderma curtisii*		
	Ganoderma tsugae		
**Gloeophyllaceae**	*Gloeophyllum sepiarium*		
**Meripilaceae**	*Hydnopolyporus fimbriatus*		
**Polyporaceae**	*Hexagonia tenuis*		
	Polyporus brumalis		
	Polyporus tricholoma		
	Trametes versicolor		
	Trametes villosa		

Familia	Nombre científico	Nombre común	Categoría de riesgo**
	Trichaptum abietinum		
	Trichaptum biforme		
**Auriscalpiaceae**	*Auriscalpium vulgare*		
	Clavicorona pyxidata		
**Russulaceae**	*Lactarius chrysorrheus*		
	Lactarius deliciosus		
	Lactarius indigo	azules	
	Lactarius piperatus		
	Lactarius salmonicolor		
	Lactarius scrobiculatus		
	Russula alutacea		
	Russula brevipes	hermanitos de San Pedro	
	Russula emetica		
	Russula foetentula		
	Russula nigricans		
**Stereaceae**	*Stereum ostrea*		
**Bankeraceae**	*Phellodon niger*		
**Auriculariaceae**	*Auricularia polytricha*		

Basado en: Zamora, E. V., *Inventario de hongos silvestres comestibles de la comunidad El Aguacate sur, municipio de Tancítaro, Michoacán,* Tesis de Licenciatura, México, Facultad de Biología, UMSNH, México, 2006.
Revisado y modificado por el Dr. Víctor Manuel Gómez Reyes, Facultad de Biología, Universidad Michoacana de San Nicolás de Hidalgo, en base a la lista pontencial de macrohongos para el Pico de Tancítaro.
**Categorías de riesgo de acuerdo a la Norma Oficial Mexicana Nom-059-Semarnat-2010, Protección ambiental-Especies nativas de México de flora y fauna silvestres-Categorías de riesgo y especificaciones para su inclusión, exclusión o cambio-Lista de especies en riesgo.
A: amenazada; P: en peligro de extinción; Pr: sujeta a protección especial; E: probablemente extinta en el medio silvestre.

Anexo Listado de fauna

Aves

Familia	Nombre científico	Nombre común	Categoría de riesgo	END
**Anatidae**	*Anas discors*	cerceta ala azul		
	Anas cyanoptera	cerceta canela		
	Anas crecca	cerceta ala verde		
	Aythya americana	pato cabeza roja		
**Cracidae**	*Penelope purpurascens*	pava cojolita	A	
**Odontophoridae**	*Dendrortyx macroura*	codorniz-coluda neovolcánica	A	E
	Cyrtonyx montezumae	codorniz moctezuma	Pr	
**Ardeidae**	*Ardea alba*	garza blanca		
	Bubulcus ibis	garza ganadera		
**Cathartidae**	*Coragyps atratus*	zopilote común		
	Cathartes aura	zopilote aura		
**Accipitridae**	*Elanus leucurus*	milano cola blanca		
	Accipiter striatus	gavilán pecho rufo	Pr	
	Accipiter cooperi	gavilán de Cooper	Pr	
	Parabuteo unicinctus	aguililla rojinegra	Pr	
	Buteo magnirostris	aguililla caminera		
	Buteo lineatus	aguililla pecho rojo	Pr	
	Buteo platypterus	aguililla ala ancha	Pr	
	Buteo nitidus	aguililla gris		
	Buteo brachyurus	aguililla cola corta		
	Buteo swainsoni	aguililla de Swainson	Pr	
	Buteo albicaudatus	aguililla cola blanca	Pr	

Familia	Nombre científico	Nombre común	Categoría de riesgo	END
	Buteo albonotatus	aguililla aura	Pr	
	Buteo jamaicensis	aguililla cola roja		
**Falconidae**	*Caracara cheriway*	caracara quebrantahuesos		
	Falco sparverius	cernícalo americano		
**Rallidae**	*Fulica americana*	gallareta americana		
**Scolopacidae**	*Actitis macularius*	playero alzacolita		
	Gallinago delicata	agachona común		
**Columbidae**	*Columba livia*	paloma doméstica		
	Patagioenas fasciata	paloma de collar		
	Zenaida asiática	paloma alas blancas		
	Zenaida macroura	paloma huilota		
	Columbina inca	tórtola cola larga		
	Columbina passerina	tórtola coquita		
	Leptotila verreauxi	paloma arroyera		
**Psittacidae**	*Rhynchopsitta pachyrhyncha*	cotorra-serrana occidental	P	E
**Cuculidae**	*Piaya cayana*	cuclillo canela		
	Geococcyx californicus	correcaminos norteño		
	Crotophaga sulcirostris	garrapatero pijuy		
**Tytonidae**	*Tyto alba*	lechuza de campanario		
**Strigidae**	*Megascops trichopsis*	tecolote rítmico		
	Bubo virginianus	búho cornudo		
	Glaucidium gnoma	tecolote serrano		
	Glaucidium brasilianum	tecolote bajeño		
	Ciccaba virgata	búho café		
	Strix varia	búho listado	Pr	

Familia	Nombre científico	Nombre común	Categoría de riesgo	END
	Asio otus	búho cara café		
	Aegolius acadicus	tecolote afilador		
**Caprimulgidae**	*Chordeiles acutipennis*	chotacabras menor		
	Nyctidromus albicollis	chotacabras pauraque		
	Caprimulgus ridgwayi	tapacamino tu-cuchillo		
	Caprimulgus arizonae	tapacamino cuerporruín-norteño		
**Apodidae**	*Cypseloides niger*	vencejo negro		
	Streptoprocne rutila	vencejo cuello castaño		
	Streptoprocne semicollaris	vencejo nuca blanca	Pr	E
	Chaetura vauxi	vencejo de Vaux		
	Aeronautes saxatalis	vencejo pecho blanco		
**Trochilidae**	*Colibri thalassinus*	colibrí oreja violeta		
	Cynanthus latirostris	colibrí pico ancho		SE
	Hylocharis leucotis	zafiro oreja blanca		
	Amazilia beryllina	colibrí berilo		
	Amazilia violiceps	colibrí corona violeta		SE
	Lampornis amethystinus	colibrí garganta amatista		
	Lampornis clemenciae	colibrí garganta azul		SE
	Eugenes fulgens	colibrí magnífico		
	Archilochus alexandri	colibrí barba negra		SE
	Stellula calliope	colibrí garganta rayada		SE
	Atthis heloisa	zumbador mexicano		E
	Selasphorus platycercus	zumbador cola ancha		SE
	Selasphorus rufus	zumbador rufo		
**Trogonidae**	*Trogon elegans*	trogón elegante		

Familia	Nombre científico	Nombre común	Categoría de riesgo	END
	Trogon mexicanus	trogón mexicano		
	Euptilotis neoxenus	trogón orejón	A	CE
**Momotidae**	*Momotus mexicanus*	momoto corona café		CE
**Alcedinidae**	*Chloroceryle amazona*	Martín-pescador amazónico		
	Chloroceryle americana	Martín-pescador verde		
**Picidae**	*Melanerpes formicivorus*	carpintero bellotero		
	Melanerpes chrysogenys	carpintero enmascarado		E
	Sphyrapicus varius	chupasavia maculado		
	Picoides scalaris	carpintero mexicano		
	Picoides villosus	carpintero velloso-mayor		
	Picoides arizonae	carpintero de Arizona		CE
	Colaptes auratus	carpintero de pechera		
**Furnariidae**	*Lepidocolaptes leucogaster*	trepatroncos escarchado		E
**Tyrannidae**	*Camptostoma imberbe*	mosquero lampiño		
	Mitrephanes phaeocercus	mosquero copetón		
	Contopus cooperi	pibí boreal		
	Contopus pertinax	pibí tengo frío		
	Contopus sordidulus	pibí occidental		
	Empidonax albigularis	mosquero garganta blanca		
	Empidonax minimus	mosquero mínimo		
	Empidonax hammondii	mosquero de Hammond		
	Empidonax oberholseri	mosquero oscuro		SE
	Empidonax affinis	mosquero pinero		CE
	Empidonax occidentalis	mosquero barranqueño		SE
	Empidonax fulvifrons	mosquero pequeño leonado		

Familia	Nombre científico	Nombre común	Categoría de riesgo	END
	Sayornis nigricans	papamoscas negro		
	Sayornis phoebe	papamoscas fibí		
	Pyrocephalus rubinus	mosquero cardenal		
	Attila spadiceus	atila		
	Myiarchus tuberculifer	papamoscas triste		
	Myiarchus cinerascens	papamoscas cenizo		
	Myiarchus nuttingi	papamoscas de Nutting		
	Myiarchus tyrannulus	papamoscas tirano		
	Pitangus sulphuratus	Luis bienteveo		
	Tyrannus melancholicus	tirano tropical		
	Tyrannus vociferans	tirano gritón		SE
	Tyrannus crassirostris	tirano pico grueso		SE
**Tityridae**	*Pachyramphus aglaiae*	mosquero-cabezón degollado		
**Laniidae**	*Lanius ludovicianus*	alcaudón verdugo		
**Vireonidae**	*Vireo bellii*	víreo de Bell		
	Vireo nelsoni	víreo enano	Pr	E
	Vireo plumbeus	víreo plomizo		
	Vireo cassinii	víreo de Cassin		SE
	Vireo solitarius	víreo anteojillo		
	Vireo huttoni	víreo reyezuelo		
	Vireo hypochryseus	víreo dorado		E
	Vireo gilvus	víreo gorjeador		
	Vireolanius melitophrys	vireón pecho castaño		CE
**Corvidae**	*Cyanocitta stelleri*	chara crestada		
	Aphelocoma ultramarina	chara pecho gris		

Familia	Nombre científico	Nombre común	Categoría de riesgo	END
	Corvus corax	cuervo común		
**Alaudidae**	*Eremophila alpestris*	alondra cornuda		
**Hirundinidae**	*Tachycineta albilinea*	golondrina manglera		
	Tachycineta thalassina	golondrina verde mar		
	Stelgidopteryx serripennis	golondrina ala aserrada		
	Petrochelidon pyrrhonota	golondrina risquera		
	Hirundo rustica	golondrina tijereta		
**Paridae**	*Poecile sclateri*	carbonero mexicano		CE
	Baeolophus wollweberi	carbonero embridado		
**Aegithalidae**	*Psaltriparus minimus*	sastrecillo		
**Sittidae**	*Sitta carolinensis*	sita pecho blanco		
	Sitta pygmaea	sita enana		
**Certhiidae**	*Certhia americana*	trepador americano		
**Troglodytidae**	*Campylorhynchus megalopterus*	matraca barrada		E
	Campylorhynchus gularis	matraca serrana		E
	Catherpes mexicanus	chivirín barranqueño		
	Thryothorus felix	chivirín feliz		E
	Thryomanes bewickii	chivirín cola oscura		
	Troglodytes aedon aedon	chivirín saltapared		
	Troglodytes aedon brunneicollis	chivirín saltapared		CE
	Henicorhina leucophrys	saltapared selvático		
**Polioptilidae**	*Polioptila caerulea*	perlita azulgris		
**Cinclidae**	*Cinclus mexicanus*	mirlo acuático norteamericano	Pr	
**Regulidae**	*Regulus satrapa*	reyezuelo de oro		
	Regulus calendula	reyezuelo de rojo		

Familia	Nombre científico	Nombre común	Categoría de riesgo	END
**Turdidae**	*Sialia sialis*	azulejo garganta canela		
	Sialia mexicana	azulejo garganta azul		
	Myadestes occidentalis	clarín jilguero	Pr	
	Catharus aurantiirostris	zorzal pico naranja		
	Catharus occidentalis	zorzal mexicano		E
	Catharus frantzii	zorzal de Frantzius	A	
	Catharus ustulatus	zorzal de Swainson		
	Catharus guttatus	zorzal cola rufa		
	Turdus assimilis	mirlo garganta blanca		
	Turdus rufopalliatus	mirlo dorso rufo		CE
	Turdus migratorius	mirlo primavera		
	Ridgwayia pinicola	mirlo pinto	Pr	E
**Mimidae**	*Mimus polyglottos*	cenzontle norteño		
	Toxostoma curvirostre	cuitlacoche pico curvo		
	Melanotis caerulescens	mulato azul		E
**Bombycillidae**	*Bombycilla cedrorum*	ampelis chinito		
**Ptilogonatidae**	*Ptilogonys cinereus*	capulinero gris		CE
**Peucedramidae**	*Peucedramus taeniatus*	ocotero enmascarado		
**Parulidae**	*Seiurus aurocapilla*	chipe suelero		
	Parkesia motacilla	chipe arroyero		
	Parkesia noveboracensis	chipe charquero		
	Mniotilta varia	chipe trepador		
	Oreothlypis superciliosa	parula ceja blanca		
	Oreothlypis celata	chipe corona naranja		
	Oreothlypis crissalis	chipe crisal	Pr	SE

Familia	Nombre científico	Nombre común	Categoría de riesgo	END
	Oreothlypis ruficapilla	chipe de coronilla		
	Geothlypis tolmiei	chipe de Tolmie		
	Setophaga ruticilla	chipe flameante		
	Setophaga petechia	chipe amarillo		
	Setophaga coronata	chipe coronado		
	Setophaga graciae	chipe ceja amarilla		
	Setophaga nigrescens	chipe negro-gris		SE
	Setophaga townsendi	chipe negroamarillo		
	Setophaga occidentalis	chipe cabeza amarilla		
	Basileuterus rufifrons	chipe gorra rufa		CE
	Basileuterus belli	chipe ceja dorada		
	Cardellina pusilla	chipe corona negra		
	Cardellina rubrifrons	chipe cara roja		SE
	Cardellina rubra	chipe rojo		E
	Myioborus pictus	chipe ala blanca		
	Myioborus miniatus	chipe de montaña		
**Emberizidae**	*Sporophila torqueola*	semillero de collar		
	Diglossa baritula	picaflor canela		
	Arremon virenticeps	atlapetes rayas verdes		E
	Atlapetes pileatus	atlapetes gorra rufa		E
	Pipilo ocai	toquí de collar		E
	Pipilo maculatus	toquí pinto		
	Aimophila rufescens	zacatonero rojizo		
	Melozone kieneri	rascador nuca rufa		E
	Melozone fusca	toquí pardo		

Familia	Nombre científico	Nombre común	Categoría de riesgo	END
	Peucaea cassinii	zacatonero de Cassini		
	Oriturus superciliosus	zacatonero rayado		E
	Spizella passerina	gorrión ceja blanca		
	Spizella pallida	gorrión pálido		SE
	Spizella breweri	gorrión de Brewer		
	Pooecetes gramineus	gorrión cola blanca		
	Chondestes grammacus	gorrión arlequín		
	Passerculus sandwichensis	gorrión sabanero		
	Ammodramus savannarum	gorrión chapulín		
	Melospiza melodia	gorrión cantor		
	Melospiza lincolnii	gorrión de Lincoln		
	Junco phaeonotus	junco ojo de lumbre		CE
**Cardinalidae**	*Piranga flava*	tángara encinera		
	Piranga rubra	tángara roja		
	Piranga ludoviciana	tángara capucha roja		
	Piranga bidentata	tángara dorso rayado		
	Piranga erythrocephala	tángara cabeza roja		E
	Pheucticus ludovicianus	picogordo pecho rosa		
	Pheucticus melanocephalus	picogordo tigrillo		SE
	Passerina caerulea	picogordo azul		
	Passerina amoena	colorín lázuli		SE
	Passerina cyanea	colorín azul		
	Passerina versicolor	colorín morado		SE
	Passerina ciris	colorín sietecolores	Pr	
	Spiza americana	arrocero americano		

Familia	Nombre científico	Nombre común	Categoría de riesgo	END
**Icteridae**	*Sturnella magna*	pradero tortilla-con-chile		
	Quiscalus mexicanus	zanate mexicano		
	Molothrus aeneus	tordo ojo rojo		
	Molothrus ater	tordo cabeza café		
	Icterus wagleri	bolsero de Wagler		
	Icterus spurius	bolsero castaño		
	Icterus cucullatus	bolsero encapuchado		SE
	Icterus pustulatus	bolsero dorso rayado		
	Icterus bullockii	bolsero calandria		SE
	Icterus galbula	bolsero de Baltimore		
	Icterus abeillei	bolsero calandria		E
**Fringillidae**	*Euphonia elegantissima*	eufonia capucha azul		
	Carpodacus mexicanus	pinzón mexicano		
	Loxia curvirostra	picotuerto rojo		
	Spinus pinus	jilguero pinero		
	Spinus notatus	jilguero encapuchado		
	Spinus psaltria	jilguero dominico		
	Coccothraustes abeillei	picogrueso encapuchado		CE
**Passeridae**	*Passer domesticus*	gorrión casero		

Orden taxonómico y nomenclatura: American Ornithologist's Union (AOU, 1998) y suplementos 42-52 (AOU, 2000; Banks, 2002, 2003, 2004, 2005, 2006, 2007, 2008 y 2009; Chesser, 2009, 2010 y 2011).
Revisado por la M. en C. Laura E. Villaseñor Gómez (*com. pers.*) de la Facultad de Biología de la Universidad Michoacana de San Nicolás de Hidalgo. **Nombres comunes:** Escalante *et al*., (1996).
**Categorías de riesgo de acuerdo a la Norma Oficial Mexicana Nom-059-Semarnat-2010, Protección ambiental-Especies nativas de México de flora y fauna silvestres-Categorías de riesgo y especificaciones para su inclusión, exclusión o cambio-Lista de especies en riesgo.
A: amenazada; P: en peligro de extinción; Pr: sujeta a protección especial.
END.: niveles de endemismo: E: endémica estricta de México; CE: cuasiendémica; SE: semiendémica.

Anfibios y reptiles

Familia	Nombre cientifico	Nombre común	Categoría de riesgo**
**Plethodontidae**	*Pseudoeurycea belli*	tlaconete pinto	A*
**Bufonidae**	*Incilius occidentalis*	sapo de pino	*
	Incilius marmoreus	sapo jaspeado	*
	Incilius perplexus	sapo confuso	*
	Rhinella marina	sapo verrugoso	
**Leptodactylidae**	*Eleutherodactylus angustidigitorum*	rana fisgona de Pátzcuaro	Pr*
	Craugastor occidentalis	rana de la Sierra Madre Occidental	*
	Leptodactylus melanonotus	rana de charco	
**Hylidae**	*Hyla arenicolor*	ranita de cañada	
	Plectrohyla bistincta	rana de pliegue	Pr*
	Tlacohyla smithi	ranita enana	*
**Microhylidae**	*Hypopachus variolosus*	rana ovejera	
**Ranidae**	*Lithobates forreri*	rana	Pr*
	Lithobates montezumae	rana de Moctezuma	Pr*
**Kinosternidae**	*Kinosternon integrum*	tortuga pecho quebrado mexicana	Pr*
**Gekonidae**	*Phylodactylus lanei*	pata de vaca	*
**Iguanidae**	*Ctenosaura pectinata*	iguana espinosa mexicana	A*
**Polychrotidae**	*Anolis nebulosus*	roño de paño	*
**Phrynosomatidae**	*Phrynosoma asio*	llora sangre	Pr*
	Sceloporus aeneus	llanerita	*
	Sceloporus dugesi	roño de suelo	*
	Sceloporus grammicus	lagartija escamosa de mezquite	Pr
	Sceloporus heterolepis	lagartija espinosa de dorso	*

Familia	Nombre cientifico	Nombre común	Categoría de riesgo**
	Sceloporus horridus	lagartija espinosa de dorso	*
	Sceloporus melanorhinus	roño de árbol	*
	Sceloporus scalaris	lagartija de montaña	Pr
	Sceloporus siniferus	lagartija escamosa castaña	
	Sceloporus torquatus	rápido barrado de meseta	
	Sceloporus utiformis	roño de suelo	*
**Scincidae**	*Plestiodon copei*	eslizón de Cope	Pr*
	Plestiodon dugesi	eslizón de Duges	Pr*
**Teiidae**	*Aspidocelis deppei*	huico	
**Boidae**	*Boa constrictor*	boa, alamacoa	A
**Anguidae**	*Barisia imbricata*	lagarto alicante	Pr*
**Colubridae**	*Conopsis biserialis*	culebra terrestre dos líneas	A*
	Conopsis nasus	culebra de tierra de la meseta mexicana	*
	Drymarchon melanurus	culebra índigo, tiricuate	
	Geophis tarascae	culebra minadora tarasca	Pr*
	Leptoderia maculata	culebra ojo de gato del suroeste	Pr*
	Leptodeira splendida	culebra ojo de gato	*
	Lampropeltis triangulum	falsa coralilla	A
	Pituophis lineaticollis	alicante, cincuate	
	Pituophis deppei	culebra sorda mexicana	A*
	Rhadinaea laureata	hojarasquera corona	*
	Rhadinaea taeniata	hojarasquera gigante	*
	Senticolis triapsis	culebra verde	
	Salvadora bairdi	culebra parchada de Baird	Pr*
	Storeria storerioides	culebra parda mexicana	*

Familia	Nombre cientifico	Nombre común	Categoría de riesgo**
	Tantilla calamarina	culebra ciempiés o encapuchada	
	Thamnophis cyrtopsis	culebra listinada cuello negro	A
	Thamnophis scalaris	culebra lineada de bosque	A*
**Viperidae**	*Crotalus tancitarensis*	cascabel de Tancítaro	*
	Crotalus basiliscus	cascabel	Pr*
	Crotalus pusillus	víbora de cascabel	A*
	Crotalus triseriatus	hocico de puerco	*
	Crotalus simus	cascabel	

Basado en:
Alvarado Díaz, J. y J. A. Campbell, *A new montane ratlesnake (Viperidae) from Michoacán, México*, en *Herpetológica,* 2004, 60(2), 281-286.
Estrada Virgen, A., *Catálogo de la diversidad de anfibios y reptiles del Municipio de Tancítaro, Michoacán, México,* Tesis de Licenciatura, Facultad de Biología, Universidad Michoacana de San Nicolás de Hidalgo, 2001.
Fuentes, J., Resp., *Ordenamiento Ecológico del Territorio Municipio de Tancítaro, Mich., Fase de diagnóstico,* Informe Final, Centro de Investigaciones en Ecosistemas, Universidad Nacional Autónoma de México, Campus Morelia, 2009, 124.
Villaseñor G., L. E. *Recuadro 6.2. La riqueza de vertebrados terrestres en las Áreas Naturales Protegidas federales*, en Villaseñor G., L. E., *La biodiversidad de Michoacán: estudio de estado,* México, Conabio, SUMA y UMSNH, Morevallado Editores, 2005, 186 (disco compacto).
Villaseñor G., L. E., *Anexo 6.4. Listado de las especies de anfibios y reptiles registrados para las Áreas Naturales Protegidas federales de Michoacán*, en Villaseñor G., L. E., *La biodiversidad de Michoacán: estudio de estado,* México, Conabio, SUMA y UMSNH, Morevallado Editores, 2005, 266 (disco compacto).
Revisado por el M. en C. Javier J. Alvarado Díaz (*com. pers.*) del Instituto de Recursos Naturales de la Universidad Michoacana de San Nicolás de Hidalgo.
**Categorías de riesgo de acuerdo a la Norma Oficial Mexicana Nom-059-Semarnat-2010, Protección ambiental-Especies nativas de México de flora y fauna silvestres-Categorías de riesgo y especificaciones para su inclusión, exclusión o cambio-Lista de especies en riesgo.
A: amenazada; Pr: sujeta a protección especial.
* Endémica (e).

Mamíferos

Familia	Especie	Nombre común	Categoría de riesgo**	DIST
**Marmosidae**	*Tlacuatzin canescens*	tlacuachín		MX
**Didelphidae**	*Didelphis virginiana*	tlacuache		AM
**Dasypodidae**	*Dasypus novencinctus*	armadillo de nueve bandas		AM
**Soricidae**	*Cryptotis goldmani*	musaraña orejillas de Goldman	Pr	MX
	Cryptotis parva soricina	musaraña orejillas mínima	Pr	AM
	Megasorex gigas	musaraña desértica sureña	A	MX
	Notiosorex crawfordi	musaraña desértica de Crawford	A	NA
	Sorex saussurei	musaraña de Saussure		MA
**Emballonuridae**	*Balantiopteryx plicata*	murciélago azulejo de sacos alares		SA
**Mormoopidae**	*Mormoops megalophylla*	murciélago		AM
	Pteronotus davyi	murciélago		SA
	Pteronotus parnelli	murciélago		SA
	Pteronotus personatus	murciélago		SA
**Phyllostomidae**	*Macrotus waterhousii*	murciélago		MA
	Micronycteris microtis	murciélago		SA
	Desmodus rotundus	vampiro		SA
	Anoura geoffroyi	murciélago		SA
	Choeroniscus godmani	murciélago		SA
	Choeronycteris mexicana	murciélago trompudo	A	NA
	Glossophaga soricina	murciélago		SA
	Hylonycteris underwoodi	murciélago		MA
	Leptonycteris curasoae	murciélago hocicudo	A	AM

Familia	Especie	Nombre común	Categoría de riesgo**	DIST
	Leptnonycteris nivalis	murciélago hocicudo mayor	A	NA
	Artibeus hirsutus	murciélago		MX
	Artibues intermedius	murciélago		SA
	Artibeus jamaicensis	murciélago		SA
	Chiroderma salvini	murciélago		SA
	Dermanura azteca	murciélago		MA
	Dermanura tolteca	murciélago		MA
	Sturnira lilium	murciélago		SA
	Sturnira ludovici	murciélago		SA
**Natalidae**	*Natalus stramineus*	murciélago		SA
**Vespertilionidae**	*Corynorhinus townsendii*	murciélago		NA
	Eptesicus furinalis	murciélago		SA
	Eptesicus fuscus	murciélago		AM
	Lasiurus cinereus	murciélago		AM
	Lasiurus intermedius	murciélago		NA
	Lasiurus xanthinus	murciélago		NA
	Myiotis californicus	murciélago		AM
	Myotis thysanodes	murciélago		NA
	Myotis velifer	murciélago		AM
	Pipistrellus hesperus	murciélago		NA
	Rhogeessa parvula	murciélago		MX
**Molossidae**	*Eumops glaucinus*	murciélago		AM
	Eumops underwoodi	murciélago		AM

Familia	Especie	Nombre común	Categoría de riesgo**	DIST
	Molossus rufus	murciélago		SA
	Molossus sinaloae	murciélago		SA
	Nyctinomops macrotis	murciélago		AM
	Tadarida brasiliensis	murciélago guanero		AM
**Canidae**	*Canis latrans cagottis*	coyote		NA
	Urocyon cinereoargenteus	zorra gris		AM
**Felidae**	*Lynx rufus*	lince, gato montés		NA
	Puma concolor azteca	puma		AM
**Mephitidae**	*Conepatus leuconotus*	zorrillo manchado		NA
	Mephitis macroura	zorrillo de dos bandas o listado		AM
**Procyonidae**	*Bassariscus astutus*	cacomixtle		N
	Nasua narica	tejón		AM
	Procyon lotor	mapache		AM
**Cervidae**	*Odocoileus virginianus*	venado cola blanca		AM
**Tayassuidae**	*Tayassu tajacu*	pecarí de collar		AM
**Sciuridae**	*Sciurus aureogaster*	ardilla gris		MA
	Notocitellus adocetus	cuinique		MX
	Otospermophilus variegatus	ardillón		NA
**Geomyidae**	*Cratogeomys gymnurus*	tuza llanera		MX
	Cratogeomys tylorhinus	tuza		MX
**Geomyidae**	*Zygogeomys trichopus*	tuza michoacana	P*	MX
**Heteromyidae**	*Liomys irroratus*	ratón espinoso		NA
	Liomys pictus	ratón espinoso		MA

Familia	Especie	Nombre común	Categoría de riesgo**	DIST
**Muridae**	*Microtus mexicanus*	meteorito		NA
	Baiomys musculus	ratón pigmeo		MA
	Nelsonia goldmani	rata cambalachera de Tancítaro	Pr	MX
	Nelsonia neotomodon	rata cambalachera diminuta	Pr	MX
	Neotoma mexicana	rata magueyera		NA
	Oryzomys couesi	rata arrocera		AM
	Osgoodomys banderanus	ratón		MX
	Peromyscus aztecus	ratón		MA
	Peromyscus difficilis	ratón		MX
	Peromyscus hylocetes	ratón		MX
	Peromyscus maniculatus	ratón		NA
	Peromyscus melanotis	ratón		NA
	Peromyscus perfulvus	ratón		MX
	Peromyscus spicilegus	ratón		MX
	Reithrodontomys chrysopsis	ratón		MX
	Reithrodontomys fulvescens	ratón		NA
	Reithrodontomys megalotis	ratón		NA
	Reithrodontomys sumichrasti	ratón		MA
	Reithrodontomys zacatecae	ratón		MX
	Sigmodon alleni	rata algodonera		MX
	Sigmodon fulviventer	rata algodonera		NA
	Sigmodon mascotensis	rata algodonera		MX
	Rattus rattus	rata gris		Int

Familia	Especie	Nombre común	Categoría de riesgo**	DIST
	Mus musculus	ratón casero		Int
**Leporidae**	*Lepus callotis*	liebre torda		NA
	Sylvilagus cunicularius	conejo de monte		MX
	Sylvilagus floridanus	conejo de monte		AM

Basado en:
Villaseñor G., L. E. *Recuadro 6.2. La riqueza de vertebrados terrestres en las Áreas Naturales Protegidas Federales*, en Villaseñor G., L. E., *La biodiversidad de Michoacán: estudio de estado,* México, Conabio, SUMA y UMSNH, Morevallado Editores, 2005, 186 (disco compacto).
Villaseñor G., L. E., *Anexo 6.4. Listado de las especies de anfibios y reptiles registrados para las Áreas Naturales Protegidas Federales de Michoacán*, en Villaseñor G., L. E., *La biodiversidad de Michoacán: estudio de estado,* México, Conabio, SUMA y UMSNH, Morevallado Editores, 2005, 266 (disco compacto).
Fuentes, J., resp., *Ordenamiento Ecológico del Territorio Municipio de Tancítaro, Mich., Fase de diagnóstico*, Informe Final, Centro de Investigaciones en Ecosistemas, Universidad Nacional Autónoma de México, Campus Morelia, 2009, 124.
Revisado por la M. en C. Ma. Concepción Apátiga Castelán, Laboratorio de Mastozoología de la Facultad de Biología de la Universidad Michoacana de San Nicolás de Hidalgo.
**Categorías de riesgo de acuerdo a la Norma Oficial Mexicana Nom-059-Semarnat-2010, Protección ambiental-Especies nativas de México de flora y fauna silvestres-Categorías de riesgo y especificaciones para su inclusión, exclusión o cambio-Lista de especies en riesgo.
A: amenazada; P: en Peligro de extinción; Pr: sujeta a protección especial.
* Endémica (e).

Anexo II

Estudios e investigaciones

El Centro de Investigaciones en Ecosistemas (CIECO-UNAM) ha desarrollado diversos estudios en el Área Natural Protegida, los cuales derivaron en el proceso de cambio de categoría de Parque Nacional a Área de Protección de Flora y Fauna. Son relevantes porque permitieron el consenso de las autoridades y las y los usuarios y pobladores del Pico de Tancítaro; son los siguientes:

1) Legislación Ambiental, Áreas Protegidas y Manejo de Recursos en Zonas Forestales Indígenas: el caso de la Microrregión del Pico de Tancítaro, Michoacán. Apoyado por Profepa, 1998-2000.

2) Paisaje y Biodiversidad en el Parque Nacional Pico de Tancítaro. Proyecto apoyado por el Fondo Mexicano para la Conservación de la Naturaleza, 1998- 2000.

3) Geomorfología, Paisaje y Recursos Naturales en el Parque Nacional Pico de Tancítaro. Proyecto PAPIIT-UNAM No. IN101900,2000-2003, 84.

4) Proyecto de Implementación de un SIG para el Manejo Forestal en una Comunidad Indígena: Comunidad de Nuevo San Juan Parangaricutiro. UNAM y fondos externos.

5) Listados taxonómicos de fauna y flora del Pico de Tancítaro. Apoyado por Conabio, 2000-2003.

Otros proyectos han sido realizados por el grupo de investigación denominado Grupo Interdisciplinario de Investigación Rural Apropiada, GIRA, A. C. en conjunto con la Comisión Nacional Forestal (Conafor) a través del PROCYMAF y son los siguientes:

1) Conafor/GIRA, A. C., Proyecto de Ordenamiento Territorial Comunitario en el Ejido La Majada, Municipio de Peribán de Ramos, Michoacán, 2004.

2) Proyecto de Captura de Carbono en el Ejido La Majada, Municipio de Peribán de Ramos.

3) Conafor/GIRA, A. C., Ordenamiento Territorial Comunitario en el Ejido La Majada, Municipio de Peribán de Ramos, Michoacán. Informe Final. En este Ordenamiento se mencionan las políticas generales para el uso del territorio de La Majada, 2004.

4) GIRA, A. C. *Evaluación rural participativa de la Comunidad Indígena de Santa Ana Zirosto, Municipio de Uruapan, Michoacán*. Grupo Interdisciplinario de Tecnología Rural Apropiada para PROCYMAF-Conafor, 2005.

Como acciones de cooperación para la conservación y de los movimientos sociales e institucionales que abogan por el orden en el cambio de uso de suelo, es importante mencionar el Foro-Taller de Desarrollo Forestal y Frutícola Sustentable realizado en junio de 2007 y coordinado por el Grupo Interdisciplinario de Investigación Rural Apropiada, GIRA, A.C., al cual asistieron instituciones (públicas, productores de aguacate,

sociales y privadas) y en el que algunos de los principales acuerdos fueron: 1. Que se aplique la ley para parar el cambio de uso de suelo, 2. Promover programas de compensación por servicios ambientales, 3. Impulsar la industria del aguacate sin menoscabar el ambiente, 4. Establecer una Norma Oficial Mexicana para regular el cultivo del aguacate, y 5. No subsidiar actividades agropecuarias en terrenos forestales, entre otros.

Por otra parte, la Comisión Nacional Forestal (Conafor) a través del Programa de Desarrollo Forestal Comunitario PROCYMAF y Serafo Consultores Ambientales, A.C. (SECOAM), realizaron en 2009 el Ordenamiento Territorial Comunitario de la Comunidad Indígena de San Salvador Combutzio Paricutín (Caltzontzin), Municipio de Uruapan, Michoacán, México.

En la Universidad Michoacana se ha realizado el estudio de aves a cargo del grupo de investigación liderado por la M. en C. Laura Villaseñor. Este estudio permitió establecer el Área de Importancia para las Aves del Tancítaro a través de la Conabio.

Asimismo, existen proyectos que inciden directamente en la planeación y el manejo del área impulsados por el INE-Semarnat y son los siguientes:

1) El papel del recurso hídrico en un Área Natural Protegida, INE/ADE-052-2003.

2) Proyecto Tepalcatepec. Colegio de Michoacán-UNAM.

3) Publicación: Velázquez, A., G. Bocco y A. Torres, Las enseñanzas de San Juan, Investigación participativa para el manejo integral de recursos naturales, México, INE-Semarnat, 2003.

Existen diversos estudios de tipo geomorfológico y geológico enfocados principalmente a la zona del Paricutín, como el trabajo de Williams, 1950; el de Segerstrom, 1950; el de Rees, 1970, y el de Bocco *et al.*, en 1997; así como las tesis de maestría sobre Geomorfología en 2000 y la tesis de doctorado sobre recurso hídrico mediante un SIG realizada en el Pico de Tancítaro en 2010 por parte del Dr. José de Jesús Fuentes Junco, quien actualmente labora como técnico académico en el Centro de Investigaciones en Ecosistemas de la UNAM, las cuales fueron realizadas en el área del Pico de Tancítaro.

Fuentes, J. de J. y L. F. Alvarado, a través de la Semarnat-Conafor, realizaron un Programa de Manejo Sustentable de Ecosistemas de Montaña en la Montaña Prioritaria Pico de Tancítaro, 2006-2011.

PARTICIPACIÓN

Este documento se realizó a través de una consulta pública, la Comisión Nacional de Áreas Naturales Protegidas reconoce la colaboración de todas y cada una de las personas, comunidades e instituciones que participaron con la aportación de su conocimiento para la elaboración de este Programa de Manejo del Área de Protección de Flora y Fauna Pico de Tancítaro.

Es posible que alguna o algunas personas que o participaron en los trabajos de investigación y en la elaboración y revisión de este Programa de Manejo pudieran haber sido omitidas por deficiencias involuntarias. Valga la presente mención como un reconocimiento a todos y cada uno de los colaboradores y colaboradoras, independientemente de su explícita mención en la siguiente relación.

Sector gubernamental

FEDERAL

Secretaría de Medio Ambiente y Recursos Naturales (Semarnat)

Instituto de Ecología y Cambio Climático (INECC)

Comisión Nacional para el Conocimiento y Uso de la Biodiversidad (Conabio)

Procuraduria Federal de Protección al Ambiente (Profepa)

Comisión Nacional Forestal (Conafor)

Secretaría de Desarrollo Agrario, Territorial y Urbano

Secretaría de Agricultura, Ganadería, Desarrollo Rural, Pesca y Alimentación (SAGARPA)

Comisión Nacional para el Desarrollo de los Pueblos Indígenas

ESTATAL

Gobierno del estado de Michoacán de Ocampo

Secretaría de Urbanismo y Medio Ambiente

Procuraduría de Protección al Ambiente de Michoacán

Consejo Estatal de Ecología

MUNICIPAL

H. Ayuntamiento de Nuevo Parangaricutiro

H. Ayuntamiento de Uruapan

H. Ayuntamiento de Tancítaro

H. Ayuntamiento de Peribán de Ramos

Sector social

Comunidad Indígena de San Salvador Combutzio Paricurtin, hoy Caltzontzin, Mpio. de Uruapan

Comunidad Indígena de Nuevo San Juan Parangaricutiro, Mpio. de Nuevo Parangaricutiro

Comunidad Indígena de Santa Ana Zirosto, Mpio. de Uruapan

Ejido La Majada, Mpio. de Peribán de Ramos

Ejido de Apo, Mpio. de Tancítaro

Ejido de Zirimondiro, Mpio. de Tancítaro

Localidad de Paraztaco, Mpio. de Peribán de Ramos

Localidad de Paso de la Nieve, Mpio. de Peribán de Ramos

Localidad de La Soledad, Mpio. de Tancítaro

Localidad de Agua Nueva, Mpio. de Tancítaro

Localidad de El Jazmín, Mpio. de Tancítaro

Localidad de La Peñita, Mpio. de Tancítaro

Localidad de Rancho Peñita San Nicolás, Mpio. de Nuevo Parangaricutiro

Sector académico

Centro de Investigaciones en Ecosistemas Campus Morelia-UNAM

Universidad Michoacana de San Nicolás de Hidalgo

Organizaciones de la sociedad civil

Grupo Interdisciplinario de Tecnología Rural Apropiada, A. C.

Integración, revisión y seguimiento a la elaboración y edición del programa de manejo

Comisión Nacional de Áreas Naturales Protegidas

Luis Fueyo Mac Donald

David Gutiérrez Carbonell

Humberto Gabriel Reyes Gómez

Ana Luisa Gallardo Santiago

José Antonio García López

Antonio Cruz Cruz

Ma. Rocío Gama Hernández

Pedro Jorge Mérida Melo

José Salvador Thomassiny Acosta

Mercedes Tapia Reyes

María de la Luz Rivero Vertiz

Mónica Flores Hidalgo

Ethel Arias Coyotl

Gabriela López Haro

María Fernanda Barrientos Carrasco

Irma Sonia Franco Martínez

Janneth del Rocío Noblecilla Maldonado

Isabel Monserrat Cid Rodríguez

Jesús Uriel Rodríguez Flores

María Esther Moreno Vázquez

FOTOGRAFÍAS

Archivo Conanp

Javier Alvarado Díaz

Laura Villaseñor Gómez

Archivo Ejido La Majada

Programa de Manejo Área de Protección de Flora y Fauna
Pico de Tancítaro
El tiraje consta de 500 ejemplares,
Se terminó de imprimir en el mes de octubre de 2014.
En los Talleres de Amelia Hernández Ugalde/SEPRIM HEUA730908AM1
3a cda. de técnicos y manuales 19-52 Lomas Estrella, Iztapalapa, D. F.

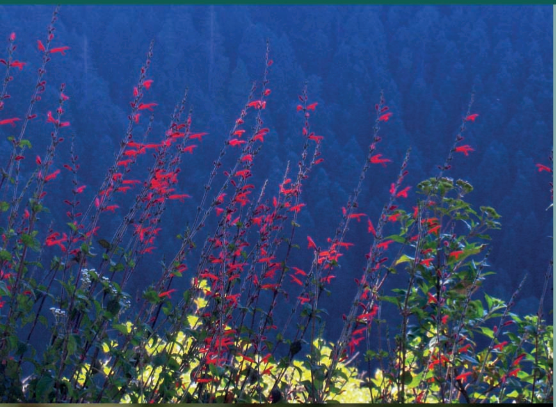

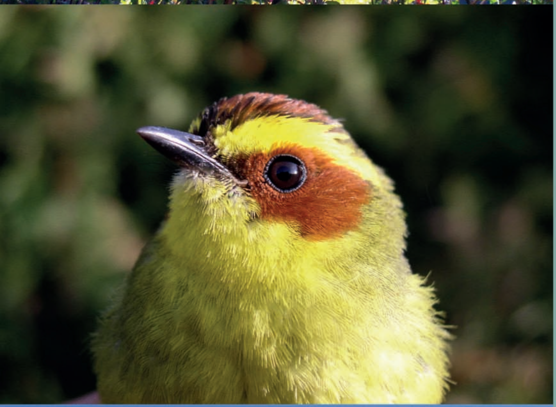

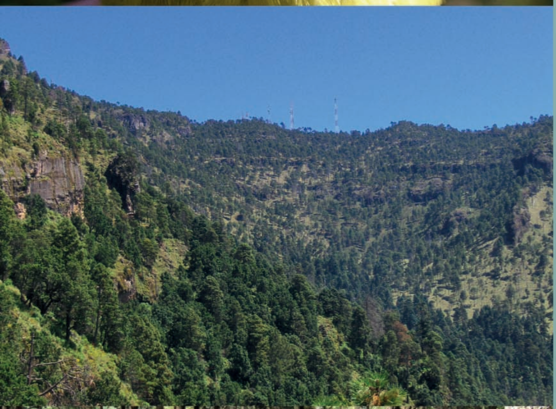

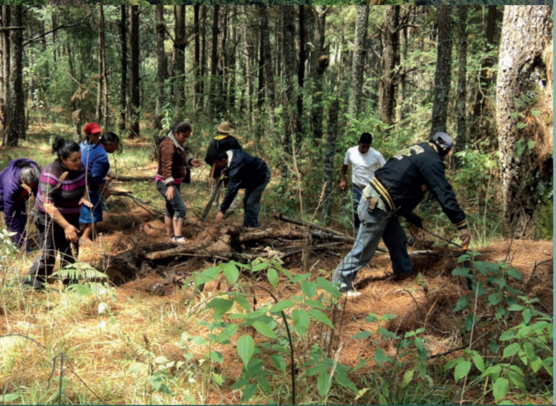

El Área de Protección de Flora y Fauna Pico de Tancítaro (APFF PT) presenta una variabilidad altitudinal que va de los 2,100 a los 3,860 metros, corresponde a la montaña más alta del estado de Michoacán. Se considera un territorio con alta importancia hidrológica, ya que cuenta con 16 cuencas, las cuales constituyen la base del desarrollo de al menos 81 poblaciones y comunidades.

El Pico de Tancítaro forma parte de las Áreas de Importancia para la Conservación de Aves (AICA) y es una de las 151 áreas consideradas como prioritarias para la conservación, ya que incluye especies únicas, tales como la tuza *Zygogeomys*, género pancrónico endémico de esta región es, la cual es muy poco conocida y la serpiente de cascabel *Crotalus tancitarensis*, considerada por algunos autores como una nueva especie.

Parte del territorio que conforma el Área Natural Protegida está inmerso en la región conocida como meseta purépecha, por lo que los aspectos culturales relacionados con la apropiación de los recursos naturales, están íntimamente ligados a la cosmovisión de las comunidades como son: Nuevo San Juan Parangaricutiro, San Salvador Combutzio Paricutin (hoy Caltzontzin) y Santa Ana Zirosto

Por ello, el Programa de Manejo del Área de Protección de Flora y Fauna es el instrumento de planeación y regulación, en el cual se reconoce la importancia ecosistémica del sitio y los aspectos socioeconómicos que soportan el uso de los recursos naturales. Su conocimiento, apropiación y uso por parte de las comunidades, ejidos y dueños de predios; abre espacios de oportunidad para el trabajo colaborativo y el diseño de mecanismos de innovación para el manejo, protección y conservación del área.

www.semarnat.gob.mx www.conanp.gob.mx
